# Integrated metabolomics and proteomics analysis reveals the accumulation mechanism of bioactive components in *Polygonatum odoratum*

**DOI:** 10.3389/fpls.2024.1487613

**Published:** 2024-12-20

**Authors:** Shuang Liang, Yang Zhao, Zhaohui Luo, Junchen Liu, Xicen Jiang, Bingxian Yang, Lin Zhang, Hongwei Fu, Zhuoheng Zhong

**Affiliations:** College of Life Sciences and Medicine, Zhejiang Sci-Tech University, Hangzhou, China

**Keywords:** *Polygonatum odoratum*, steroidal saponins, flavonoids, metabolomics, proteomics

## Abstract

*Polygonatum odoratum* (Mill.) Druce is rich in bioactive components with high medicinal value. To maximize the clinical benefits, it is of great significance to efficiently extract key bioactive components from appropriate growth stages in which they are most abundant. In this study, we analyzed the changes of metabolite accumulation and protein expression in *P. odoratum* rhizomes at different growth stages using targeted metabolomics combined with proteomics, and identified a total of 1,237 differentially abundant metabolites (DAMs). Flavonoids accumulated most in winter, and the biosynthesis pathways associated with flavonoids, isoflavonoids, flavones and flavonols exhibited significant differentially expressed proteins (DEPs). Among them, PGT, FLS, CYP75B1, HIDH, IF7MAT, and UFT73C6 were positively correlated with flavonoid accumulation. Steroid saponins accumulated most in spring, and the biosynthetic pathways of steroid and brassinosteroid biosynthesis exhibited DEPs. Among them, FDFT1, TM7SF2, DHCR7, CAS1, and 3BETAHSDD were positively correlated with steroidal saponin accumulation. In summary, these results revealed the accumulation of secondary metabolites *P. odoratum* in different growth stages, which can provide an effective reference for the extraction of specific bioactive components and the study of their regulatory mechanisms.

## Introduction

*Polygonatum odoratum* (Mill.) Druce is a perennial herb in the *Liliaceae* family that is traditionally used as both food and medicine (Liu et al., 2024). It has been included in all editions of the Chinese pharmacopoeia, with a history of use spanning centuries (Wang LL. et al., 2021). According to traditional Chinese medicine, the rhizomes of *P. odoratum* are effective in nourishing yin and moisturizing dryness, quenching thirst, benefiting the stomach and promoting the production of body fluids (Wang LL. et al., 2021; Liu et al., 2024). Because of the sweet flavor and tonic effect of the rhizomes of *P. odoratum*, the local inhabitants of Hunan and Guangxi provinces often use them to make stews, soups, wines, medicinal dishes, etc., which have gradually formed the local eating habits (Liu et al., 2021; Xu et al., 2023). Previous studies have shown that *P. odoratum* is rich in polysaccharides, flavonoids, saponins, and other potentially pharmaceutically active compounds, with anti-aging, hypoglycemic, anti-fatigue, immunoregulatory, antioxidant, antiviral, and other potential effects (Meng et al., 2020; Luo et al., 2022; Bi et al., 2023). Consequently, it is often processed into tablets, health food as well as cosmetic and skin care products (Li et al., 2015; Liu et al., 2021). Thus, its utilization in food, cosmetics and healthcare products has broad market prospects.

It is generally believed that the active ingredients of three-year-old *P. odoratum* can meet the standard or medicinal use, and considering the efficiency of land turnover and economic benefits, it is most appropriate to harvest *P. odoratum* when it has reached three years of growth (Wang et al., 2017). Numerous studies investigated the germplasm resources, ecological cultivation, chemical composition, processing and pharmacological effects of *P. odoratum*. Wang et al. studied the effect of different fertilizers on the photosynthetic characteristics and quality of *P. odoratum (* Wang et al., 2019). Wang et al. investigated the dynamic changes in the accumulation of active ingredients in *P. odoratum* over different growth years and harvesting periods (Wang et al., 2017). Zhou et al. isolated 25 different steroidal saponins from *P. odoratum*, 5 of which exhibited effective inhibitory effects on cancer cells (Guo et al., 2012). Xia et al. investigated the effects of different processing treatments on the antioxidant activity of *P. odoratum* flavonoids (Zhou et al., 2021). In a comparative metabolomic analysis of closely related *Polygonatum sibiricum* Red and *P. odoratum*, it was found that *P. odoratum* contained more abundant secondary metabolites than *P. sibiricum (* Xia et al., 2018 *).* Recently, flavonoids (especially homoisoflavanones) and steroidal saponins are increasingly being recognized for their pharmacological activities (Wang et al., 2024), but few studies focused on changes in the accumulation of flavonoids and steroids in *P. odoratum* at different stages of growth via multi-omics approaches. Metabolite stores in the plant body are dynamic, and the levels of bioactive compounds have been reported to vary with the growth stage, seasons and harvesting period (Zhang et al., 2020). Metabolomics allows the study of metabolite composition and changes, thus revealing the overall metabolic response and changes at different growth stages. As enzymes are expressed as a direct consequence of gene activation (Qi et al., 2023), proteomics technology can directly reflect the gene expression of *P. odoratum* at different growth stages. For example, the combination of metabolomics and proteomics has been utilized to elucidate the anabolic pathways and regulatory processes of flavonoids in *Ginkgo biloba* L. leaves, seeds and exotesta at different developmental stages, increasing our understanding of the regulation of active phytochemicals (Guo et al., 2022).

In this study, conducted integrated metabolomic and proteomic analyses to gain a comprehensive understanding of metabolite transformation and protein expression in *P. odoratum* at different growth stages. This study aimed to determine the synthesis and regulatory mechanisms of secondary metabolites at different growth stages of *P. odoratum*. In addition, it provides a reference for choosing the appropriate time to extract the main active components. To the best of our knowledge, this is the first time that a multi-omics approach has been utilized to systematically investigate the molecular mechanisms of secondary metabolite changes at different growth stages of *P. odoratum*.

## Materials and methods

### Plant materials

**Figure 1:**
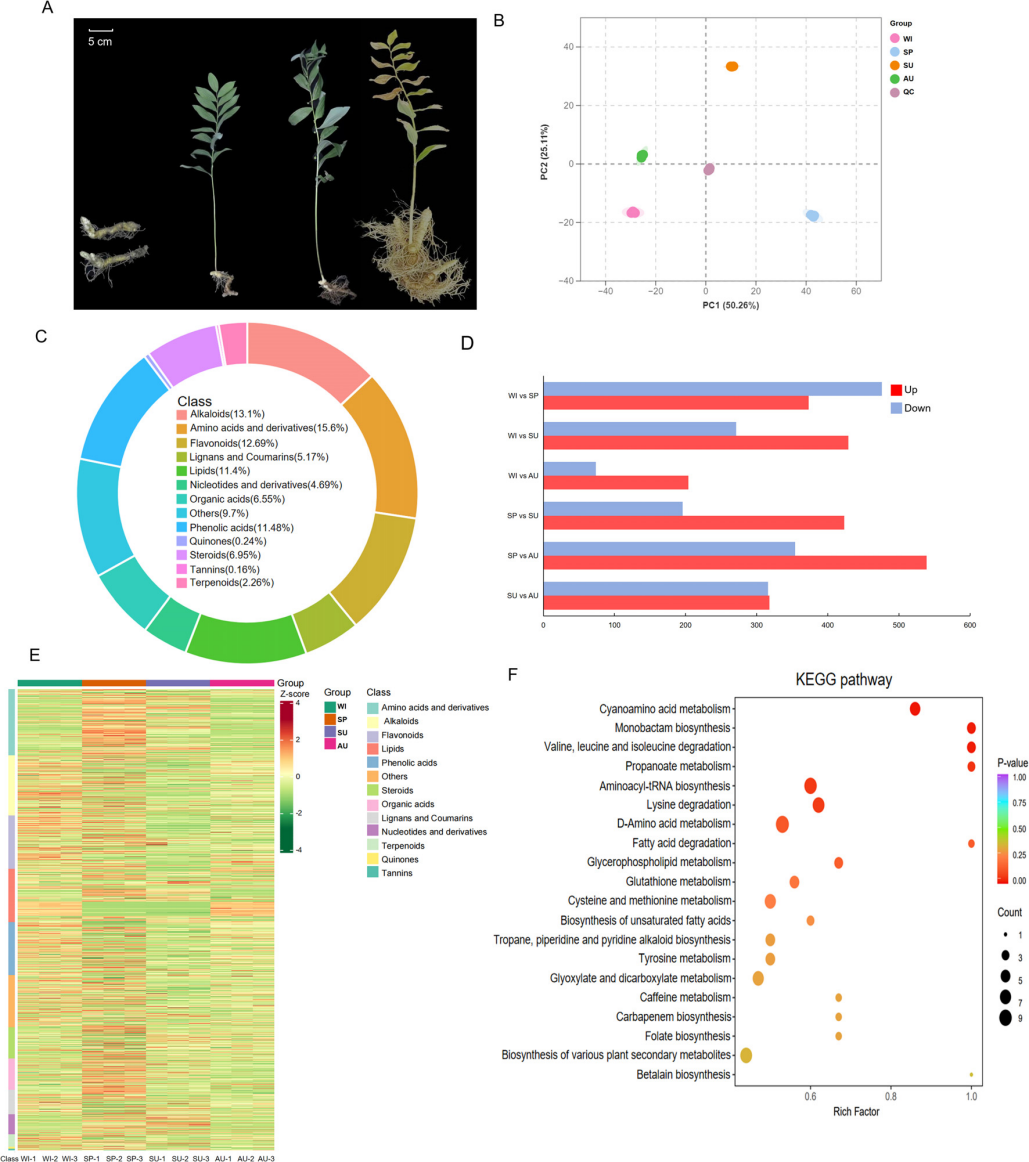
Metabolomic analysis of *P. odoratum* at different growth stages. **(A)** Growth status of *P. odoratum* at different stages, from left to right, winter (February), spring (May), summer (August), and autumn (November). **(B)** PCA analysis of metabolites from samples of *P. odoratum* rhizomes at different growth stages. **(C)** Classification of metabolites at different growth stages. **(D)** Numerical representations of metabolites at different growth stages. **(E)** Heatmap of the relative abundance of differential metabolites. **(F)** KEGG pathway enrichment analysis of differential metabolites.

The *P. odoratum* rhizomes used for the study were planted for 3 years in a regulated experimental field in Nanling County, Wuhu, Anhui, People’s Republic of China (31°33′N, 118°38′E). Fresh *P. odoratum* rhizomes were collected on February 2 (winter, abbreviated as WI), May 2 (spring, abbreviated as SP), August 2 (summer, abbreviated as SU), and November 2 (autumn, abbreviated as AU) 2022 (**Figure 1A**). They were identified by Prof. Hongwei Fu (Zhejiang Sci-Tech University, China) and all samples were kept at the College of Life Science and Medicine, Zhejiang Sci-Tech University. The *P. odoratum* was collected in the third year after planting in the same plot with uniform fertilizer, irrigation and insect spray management. At each sampling, fresh rhizomes from a total of 20 plants were dug from a total of five points (four corners and intersections of the same plot) and processed together. After washing with purified water, the rhizomes were cut into 0.5 cm pieces, flash-frozen in liquid nitrogen and stored at -80°C for subsequent experiments.

### Widely targeted metabolome analysis

Biological samples were dried using a freeze dryer (Scientz-100F) and then ground to a powder using an MM 400 grinder (Retsch, China). Then, 50 mg of the powder was weighed using an MS105DM electronic balance (Sartorius, Germany) and 1200 μL of 70% aqueous methanol with internal standard pre-cooled to -20°C was added. The samples were vortexed six times, centrifuged, and the filtered supernatant used as the sample for UPLC-MS/MS analysis.

Metabolomics analyses were performed by Met-ware Biotechnology Co., Ltd. (Wuhan, China), using peak area integral analysis of all metabolites of different samples by mass spectrometry and integral correction of metabolite peaks of different samples. For two-group analysis, differentially abundant metabolites (DAMs) were determined based on the *P*-value (*P* < 0.05) and absolute Log2FC (|Log2FC| ≥ 1.0). For multi-group analysis, DAMs were determined based on the *P*-value (*P* < 0.05, ANOVA). Metabolic pathways related to the DAMs were retrieved from the Kyoto Encyclopedia of Genes and Genomes (KEGG) database.

### Proteomic analysis

Proteins were extracted from the samples by acetone precipitation. Briefly, samples were ground to a fine powder in liquid nitrogen and homogenized, and 100 mM Tris HCl (pH 7.6), 1 mM phenylmethylsulfonyl fluoride (PMSF), and 2 mM ethylenediaminetetraacetic acid (EDTA) extraction solution were added. Then, the protein samples were boiled for 15 min, lysed by sonication on ice for 10 min, and centrifuged to obtain a clear protein solution. A 4-fold volume of frozen acetone was added to the protein solution, which was allowed to precipitate overnight at -20°C, and centrifuged at 4°C. The resulting precipitate was washed with cold acetone and dissolved in 8 M urea. Finally, the protein concentration was determined using a BCA assay kit according to the manufacturer’s instructions (Ross et al., 2004; Jing et al., 2010).

An equal amount of protein from each sample was used for trypsin digestion. To the supernatant, 8 M urea was added to 200 µl, followed by reduction with 10 mM DTT for 45 min at 37°C and alkylation with 50 mM iodoacetamide (IAM) for 15 min at room temperature in the dark. Then, a 4-fold volume of ice-cold acetone was added and the peptides were allowed to precipitate for 2 h at -20°C. After centrifugation, the protein precipitate was air-dried, re-suspended in 200 µl of 25 mM ammonium bicarbonate solution with 3 µl of 1:50 trypsin (trypsin: protein (m/m), Promega), and digested at 37°C overnight. After digestion, peptides were desalted using a C18 cartridge (IonOpticks, Australia), then dried in a vacuum concentrator, concentrated by vacuum centrifugation, and redissolved in 0.1% (v/v) aqueous formic acid (Jing et al., 2012; Florian et al., 2018).

Samples were separated using a nanoliter flow rate NanoElute UPLC system (Bruker, Germany) and then subjected to mass spectrometry data acquisition using the DDA-PASEF mode of the timsTOF Pro2 mass spectrometer (Bruker, Germany). The library search software used for sample DIA mass spectrometry data was DIA-NNN (v1.8.1), and spectral libraries were used to reanalyze the DIA data for protein quantification. The precursor ions and protein levels of the FDR were filtered at 1%, and the post-filtered data were ready for subsequent biosignature analysis (Yu et al., 2020). For proteomic analysis, three biological replicates were included for each group. Differences with *P*-values <0.05 were considered statistically significant.

### Bioinformatical analysis

*Cluster analysis*: K-means analysis was performed on differential metabolites to obtain clustering information of differential proteins based on their correlation with differential metabolites. K-Means analysis was performed using Metware Cloud, a free online platform for data analysis (https://cloud.metware.cn).

*Principal component analysis* (PCA): the prcomp function in R soft­ware (version R 4.2.0, https://www.R-project.org/) (R, 2022) was used for PCA analysis.

*Gene Ontology (GO) enrichment*: Gene Ontology enrichment of DEPs was analyzed using the topGO function in R software (version 4.2.0).

### qRT-PCR analysis

qRT-PCR was used to determine the expression of genes related to steroidal saponin and flavonoid synthesis. Three biological replicates were included in each group of samples. Primers were designed using Primer 5.0 software (**Supplementary Table S1**), and SYBR ^®^Green Pro Taq HS qPCR Kit (Accurate Biotechnology Co., Ltd, Hunan, China) was used for qRT-PCR on an ABI7500 fluorescence quantitative PCR instrument (Applied Biosystems, CA, USA). The relative expression levels were calculated using the 2^− △△Ct^ method (Wu et al., 2020).

## Results

### Metabolite profiles

The results of the principal component analysis showed that metabolic profiles could be clearly distinguished between the different sample groups, with biological samples clustered together (**Figure 1B**), indicating good repeatability and reliability of metabolomics analysis. In this study, a total of 1,460 known metabolites were detected in winter (February), spring (May), summer (August) and autumn (November) rhizome samples using a UPLC-MS/MS system. These included 1,237 differentially abundant metabolites (DAMs, **Figure 1C**), which were categorized as amino acids and derivatives, alkaloids, flavonoids, phenolic acids, lipids, steroids, organic acids, lignans and coumarins, nucleotides and derivatives, terpenoids, quinones, and tannins (**Figure 1C**; **Supplementary Table S2**).

### Changes of secondary metabolites at different growth stages

Pairwise comparisons revealed that spring-harvested rhizomes showed the highest number of upregulated metabolites, followed by those harvested in winter (**Figure 1D**). Based on the data of differential metabolite content and heatmap based on Z-score normalization, we found that metabolite accumulation showed different trends at different growth stages (**Figure 1E**). The differential metabolites were mainly enriched in amino acid metabolism, specifically valine, leucine and isoleucine degradation, as well as propanoate metabolism (**Figure 1F**).

### Hierarchical clustering of differentially accumulated metabolites

**Figure 2:**
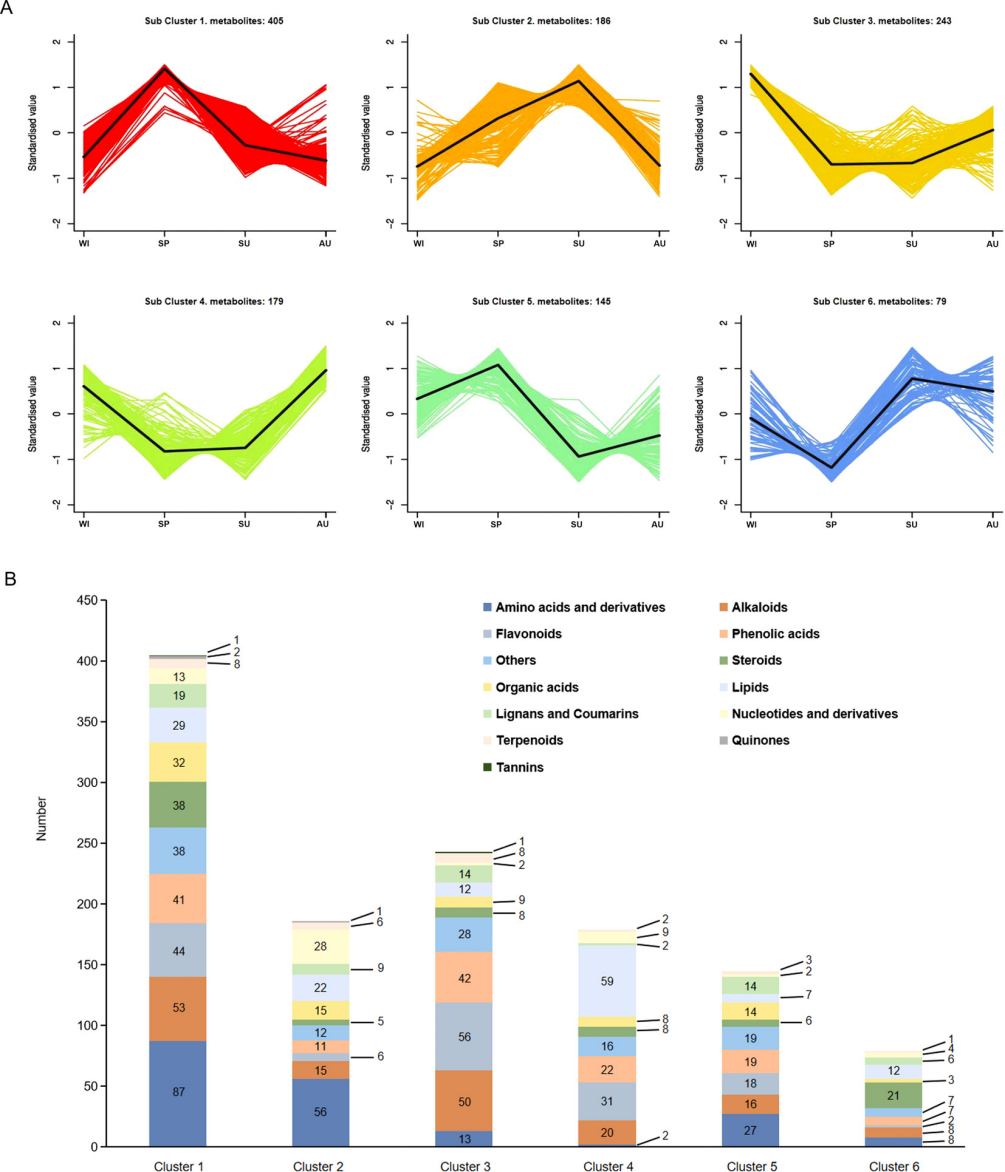
Changes in the accumulation of metabolites. **(A)** Cluster analysis of differential metabolites. The X-axis represents different growth stages, the y-axis represents the standardized value of metabolite content. In each cluster, the black line in the middle represents the average content. **(B)** Classification of metabolites in each subcluster.

In order to gain a deeper understanding of the changes in of secondary metabolites at different growth stages, six clusters with different expression trends across the growth stages were generated using the K-means algorithm (**Figure 2A**). Subcluster 1 was the largest and contained 405 metabolites, while subclusters 2-6 contained 186, 243, 179, 145 and 79 metabolites, respectively (**Figure 2B**). Subclusters 1 and 5 show the types and quantities of metabolites upregulated in spring, with the highest accumulation of amino acids and their derivatives. Compared with the other three seasons, alkaloids, phenolic acids, steroids, and organic acids also accumulated more abundantly in spring. Subclusters 2 and 6 show the types and quantities of metabolites upregulated in summer, with the highest accumulation observed for amino acids and their derivatives, followed by nucleotides and their derivatives. Subclusters 3 and 4 show the number and types of metabolites upregulated in winter, with flavonoids accumulating the most, followed by lipids. Subclusters 4 and 6 show the types and quantities of metabolites upregulated in autumn, with lipid accumulation being the highest, followed by flavonoids. Among the metabolites detected in the four seasons, steroids and flavonoids were the main bioactive compounds, and more and more pharmacological effects have been discovered, offering important guidance for the clinical utilization of herbal resources. By combining heatmap and sub-cluster analyses, we focused on steroidal compounds in subcluster 1 and flavonoid compounds in subcluster 3. Subcluster 1 had 38 steroidal compounds, including 12 spirostane saponins, 16 isospirostane saponins, 8 furostane saponins (**Supplementary Table S3**) and 2 steroids. Subcluster 3 had 56 flavonoids, including 19 other flavonoids (14 homoisoflavanones), 12 flavones, 8 flavanones, 7 flavonols, 5 chalcones, 2 flavanonols, 2 isoflavones and 1 flavanol (**Supplementary Table S4**). We conducted KEGG functional enrichment analysis on metabolites in clusters 1 and 3, and selected the top 20 based on the *P*-value ranking. Differential metabolites from cluster 1 were mainly enriched in amino acid biosynthesis and metabolism, ABC transporters, linoleic acid metabolism, nucleotide metabolism, glycerolipid metabolism, and biosynthesis of various plant secondary metabolites (**Supplementary Figure S1A**). Differential metabolites from cluster 3 were mainly enriched in the biosynthesis of flavonoids, isoflavonoid biosynthesis, amino acid biosynthesis and metabolism, plant hormone signal transduction, and biosynthesis of secondary metabolites (**Supplementary Figure S1B**).

### DEPs analysis

In order to elucidate the mechanisms underlying the changes in the content of steroid and flavonoid compounds, we performed proteomic analysis of rhizomes from different growth stages of *P. odoratum*. By using a protein analysis system, we identified 43,849 different peptides and 7,027 proteins in all 12 samples. Based on the *P* < 0.05 screening criterion, 3,864 differentially expressed proteins (DEPs) were obtained. To investigate their biological functions, we categorized the identified DEPs into 24 classes using the COG database. The largest category was general function prediction only (425 DEPs), followed by posttranslational modification, protein turnover, chaperones (369 DEPs), translation, ribosomal structure and biogenesis (236 DEPs), energy production and conversion (235 DEPs), carbohydrate transport and metabolism (216 DEPs), signal transduction mechanisms (198 DEPs), intracellular trafficking, secretion and vesicular transport (172 DEPs), as well as secondary metabolite biosynthesis, transport and catabolism (134 DEPs) (**Supplementary Figure S2**). Classification has shown that many DEPs are involved in the transport of substances and metabolic processes. Proteins with these functions may play an important role in the synthesis and transport of secondary metabolites.

### Hierarchical clustering of DEPs

**Figure 3:**
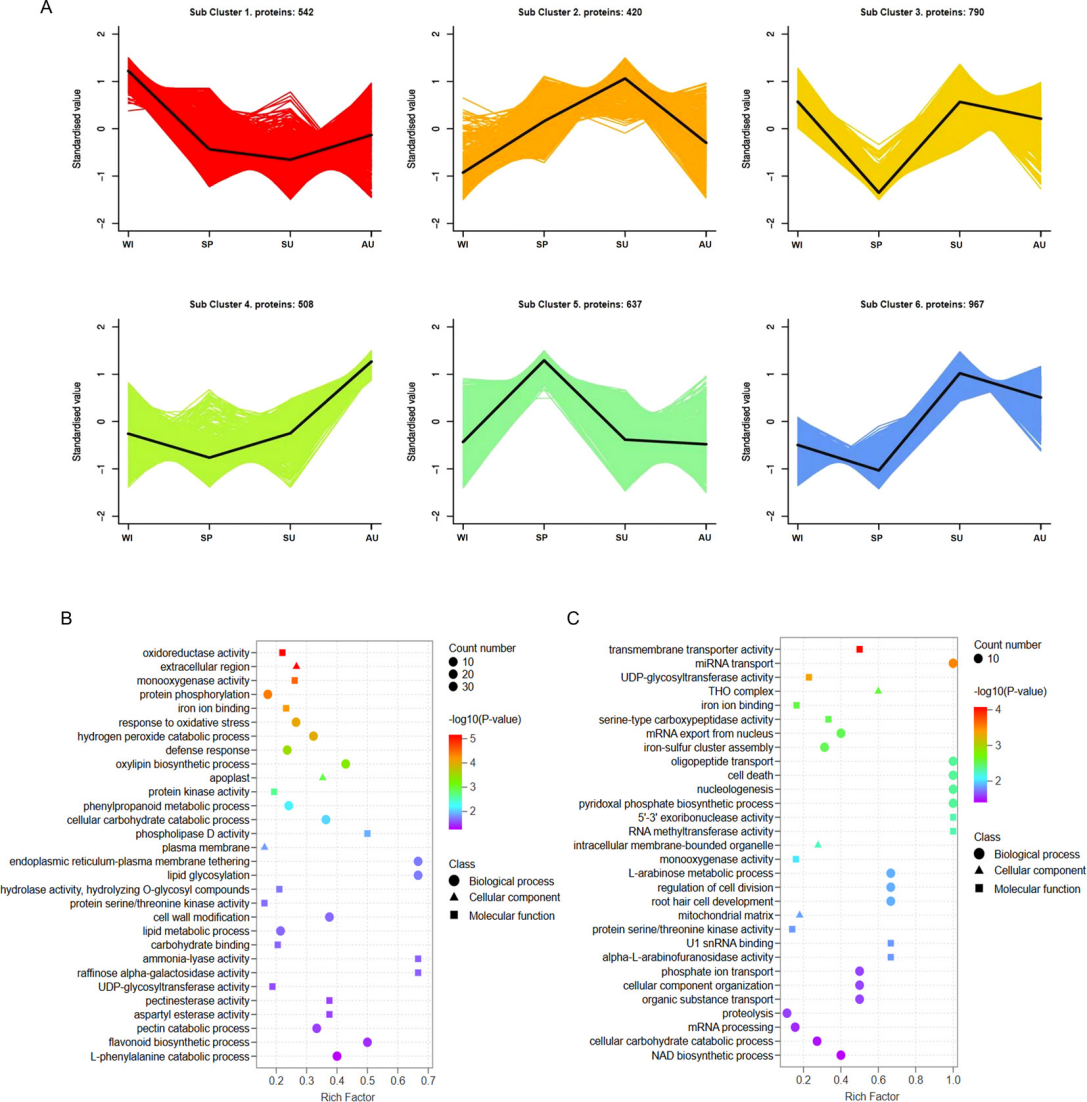
Changes of protein expression. **(A)** Cluster analysis of differentially expressed proteins. The X-axis represents different growth stages, the y-axis represents the standardized value of protein expression. In each cluster, the black line in the middle represents the average protein expression level. **(B)** GO enrichment analysis of subcluster 5 proteins. **(C)** GO enrichment analysis of subcluster 1 proteins.

We also analyzed the changes in the expression of DEPs at different growth stages, and categorized the DEPs at different growth stages into six clusters. Based on this, we focused on protein subcluster 5 and protein subcluster 1 (**Figure 3A**), which had the same trend as metabolic subcluster 1 and metabolic subcluster 3. To better analyze the functions of DEPs in subcluster 5 and subcluster 1, we performed GO analysis and selected the top 30 based on the *P*-value rankings of GO enrichment analysis. In the GO annotations of subcluster 5, the DEPs were assigned to 13 biological processes (mainly protein modification, response to stimuli and defense, metabolic processes, and bioregulation), 3 cellular components (plasma ectodomain, plasma membrane and endoplasmic reticulum membrane) and 14 molecular functions (mainly enzyme activity and binding activity)(**Figure 3B**). In the GO annotation of subcluster 1, the DEPs were assigned to 14 biological processes (mainly protein synthesis and hydrolysis, cell cycle, and metabolic processes), 3 cellular components (THO complex, intracellular membrane-bounded organelle, and mitochondrial matrix) and 13 molecular functions (mainly enzyme activities and binding activities) (**Figure 3C**). GO enrichment analysis indicated that the expression of proteins in the rhizomes of *P. odoratum* at different growth stages may be related to antioxidant activity, binding, stimulatory responses, catalytic activity, metabolic processes, bioregulation, enzyme activity and regulation of molecular functions.

### KEGG functional analysis of DEPs

**Figure 4:**
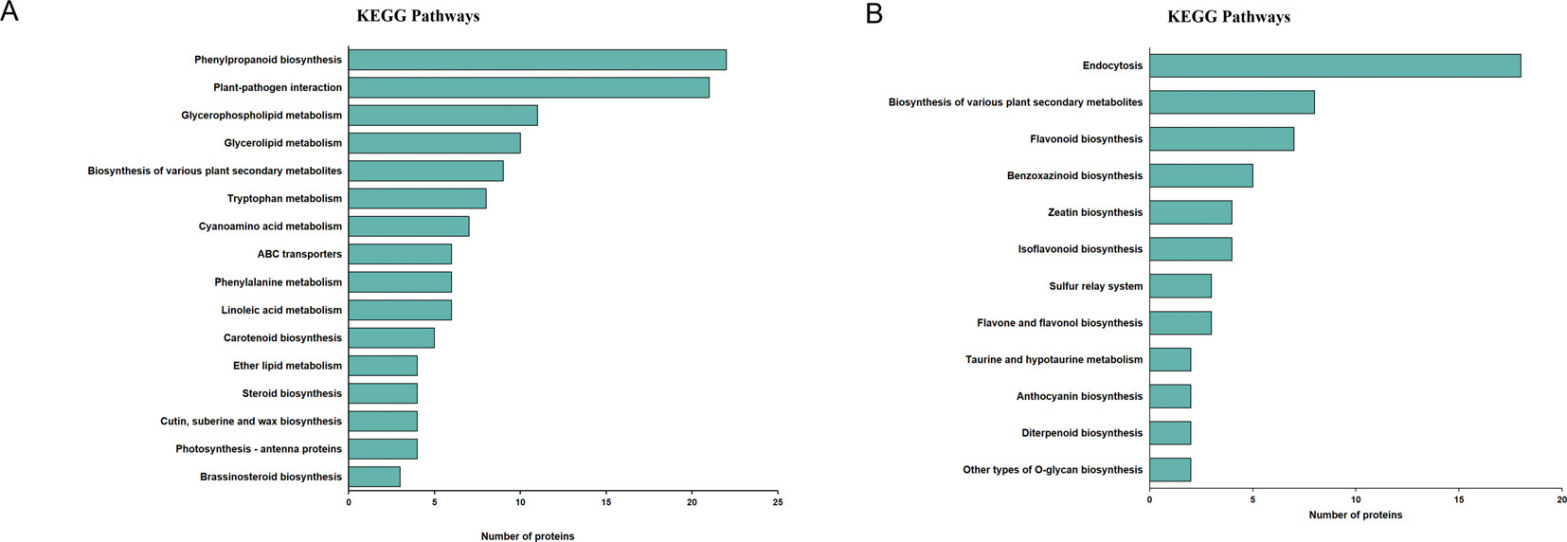
Changes of protein expression. **(A)** KEGG pathway enrichment analysis of subcluster 5 proteins. **(B)** KEGG pathway enrichment analysis of subcluster 1 proteins.

To assess the pathway that are likely involved in changes of secondary metabolites, DEPs from different subclusters were analyzed using the KEGG database. The DEPs in protein subcluster 5were found to be significantly enriched in 16 pathways (*P* < 0.05), mainly related to plant secondary metabolites, lipid metabolism and amino acid metabolism. Steroid biosynthesis (ko00100) and brassinosteroid biosynthesis (ko00905), which are associated with steroidal saponin synthesis, had significant DEPs (**Figure 4A**). The DEPs in protein subcluster 1 were found to be significantly enriched in 12 pathways (*P* < 0.05), mainly related to plant secondary metabolites and phytohormone metabolism. Flavonoid biosynthesis (ko00941), isoflavonoid biosynthesis (ko00943), as well as flavone and flavonol biosynthesis (ko00944) (**Figure 4B**), which are related to flavonoid synthesis, were found to have significant DEPs in subcluster 1. These results suggest that proteins from different physiological and metabolic pathways work together to regulate the accumulation of steroidal saponins and flavonoids.

### Analysis of steroid and flavonoid biosynthesis pathways

**Figure 5:**
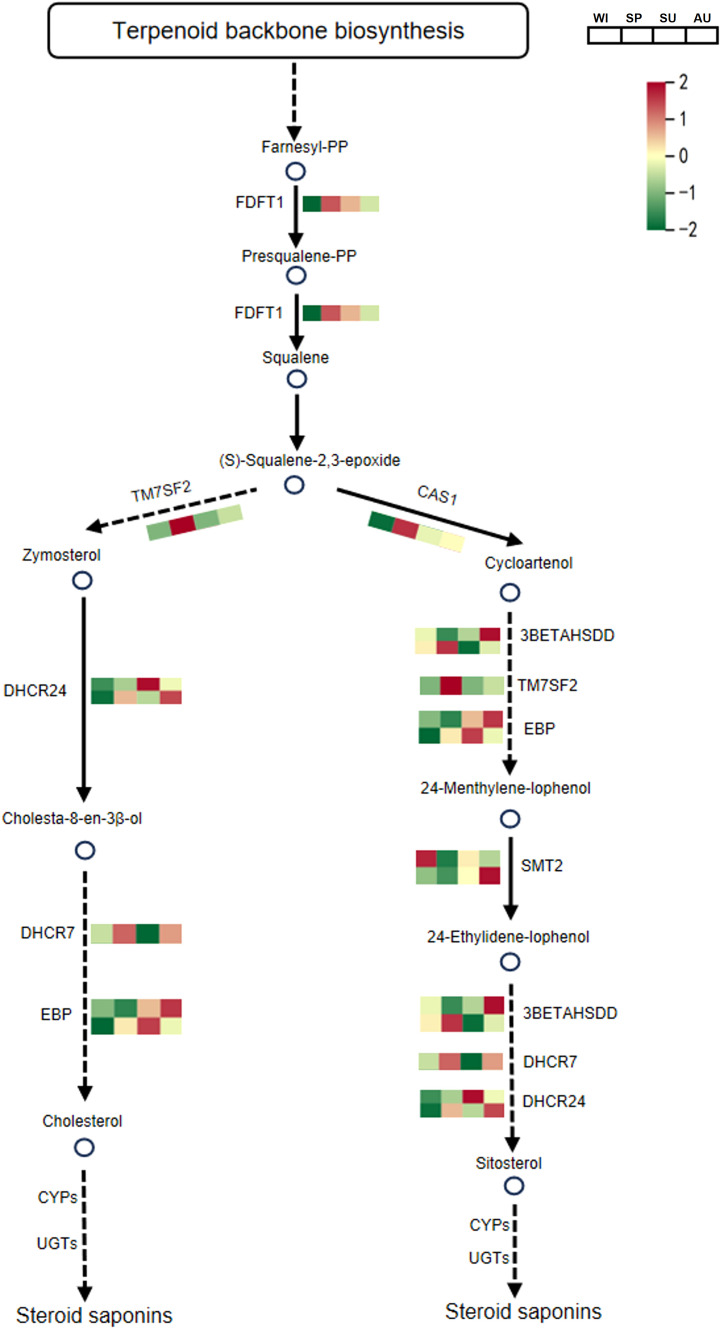
The biosynthetic pathway of steroidal saponins in *P. odoratum*. The average expression is shown in the heatmaps. One-step reactions are represented with solid lines while dashed lines represent multi-step reactions. Circles represent the metabolites in pathways and circles filled with red color represent the metabolites that were identified and significantly accumulated. FDFT1, farnesyl-diphosphate farnesyltransferase; TM7SF2, Δ14-sterol reductase; DHCR24, Δ24-sterol reductase; DHCR7, 7-dehydrocholesterol reductase; EBP, cholestenol Δ-isomerase; CAS1, cycloartenol synthase; 3BETAHSDD, plant 3β-hydroxysteroid-4α-carboxylate 3-dehydrogenase; TM7SF2, Δ14-sterol reductase; SMT2, 24-methylenesterol C-methyltransferase; CYPs, cytochromes P450s; UGTs, UDP-glycosyltransferases.

**Figure 6:**
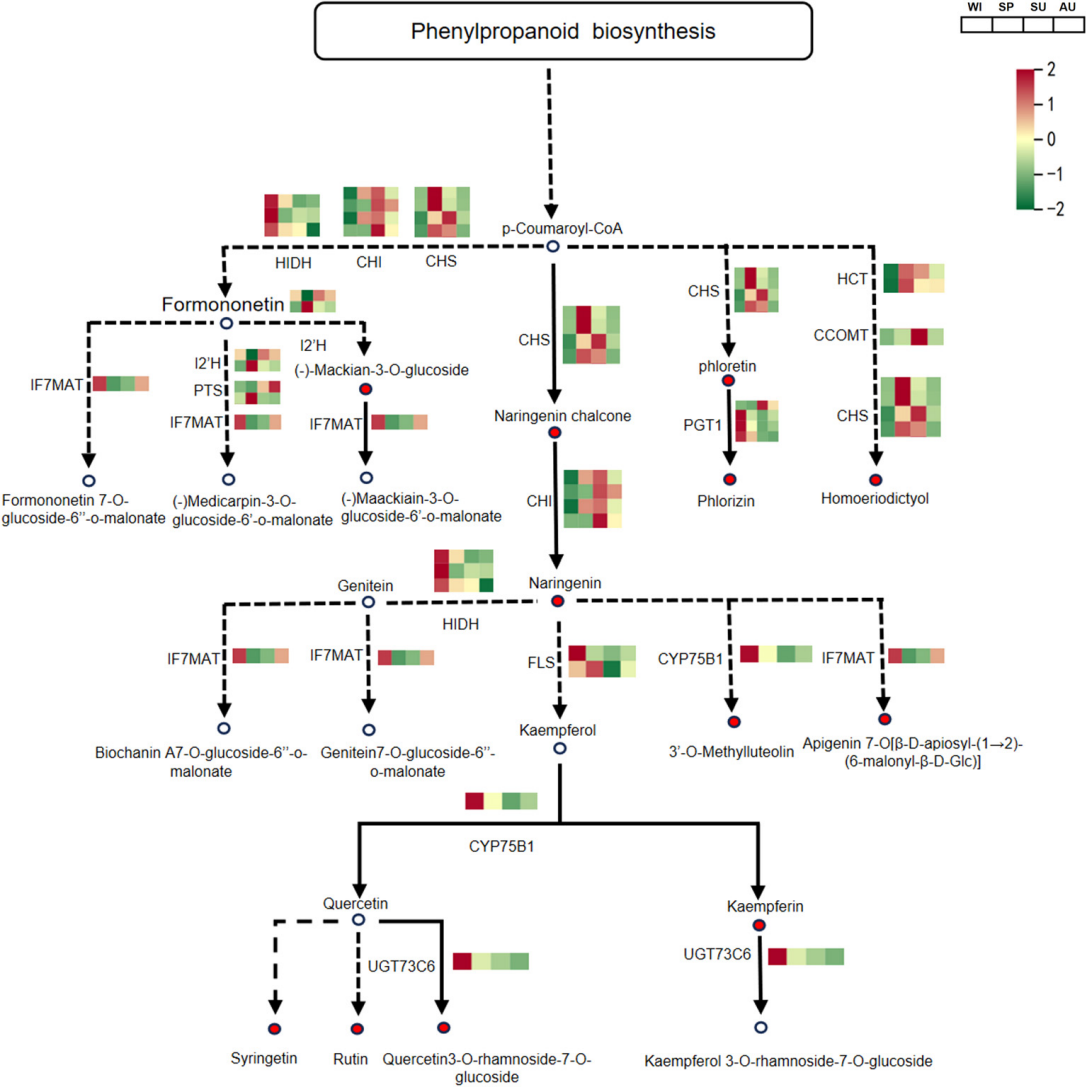
The biosynthetic pathway of flavonoids in *P. odoratum*. the average expression levels are shown in the heatmaps. One-step reactions are represented with solid lines while the dashed lines represented multi-step reactions. The circles represent metabolites in pathways and circles filled with red color represent metabolites that were identified and significantly accumulated. CHS, chalcone synthase; CHI, chalcone isomerase; HIDH, 2-hydroxyisoflavanone dehydratase; IF7MAT, isoflavone7-O-glucoside-6’’-O-malonyltransferase; I2’H, isoflavone/4’-methoxyisoflavone 2’-hydroxylase; PTS, pterocarpan synthase; FLS, flavonol synthase; CYP75B1, flavonoid 3’-monooxygenase; UGT73C6, flavonol-3-O-L-rhamnoside-7-O-glucosyltransferase; PGT1, phlorizin synthase; HCT, shikimate O-hydroxycinnamoyl transferase; CCOMT, caffeoyl-CoA O-methyltransferase.

In the steroid biosynthesis pathway, a total of 10 DEPs associated with steroid synthesis were identified. Among them, FDFT1, TM7SF2, CAS1, DHCR7, 3BETAHSDD and TM7SF2 were significantly upregulated in spring, and these proteins may contribute to the accumulation of steroidal saponins (**Figure 5**; **Supplementary Table S5**). In the flavonoid biosynthesis pathway, a total of 12 DEPs associated with flavonoid synthesis were detected. Among them, HIDH, IF7MAT, PGT1, FLS, CYP75B1 and UGT73C6 were significantly upregulated in winter, and these proteins may promote the accumulation of flavonoid compounds (**Figure 6**; **Supplementary Table S6**). The relative abundance of differential metabolites related to steroid and flavonoid metabolism differed significantly among groups. The significant differences in these DAMs at different times provide a basis for selecting the appropriate time to extract specific components.

### Correlation analysis of DAMs and DEPs

Based on metabolomic and proteomic data, KEGG pathway enrichment was conducted for comprehensive analysis. A bubble plot was drawn using the KEGG pathways enriched in both independent datasets, showing only the top 25 pathways based on *P*-value ranking (**Supplementary Figure S3**). The common pathways identified in both the proteome and metabolome included flavonoid biosynthesis, biosynthetic of various plant secondary metabolites, glycyl and dicarboxylate metabolism, alpha linolenic acid metabolism and stilbene biosynthesis. Integrated omics analysis showed that these metabolites and proteins mainly participate in primary and secondary metabolism.

In order to better understand the relationship between metabolomics and proteomics, we conducted Pearson correlation analysis on the biosynthetic pathways of steroidal saponins and flavonoids. Pearson correlation analysis of 38 steroidal saponins from metabolic subcluster 1 and the related DEPs in protein subcluster 5 showed that FDFT1, TM7SF2, DHCR7, CAS1 and 3BETAHSDD were positively correlated with the accumulation of steroidal saponins (*P* < 0.05), However, SMT2 was negatively correlated with steroidal saponins (**Supplementary Figure S4A**). Pearson correlation analysis of 56 flavonoid compounds in metabolic subcluster 3 and related DEPs in protein subcluster 1 showed that PGT, FLS, CYP75B1, HIDH, IF7MAT and UFT73C6 were positively correlated with flavonoid accumulation (*P* < 0.05). However, CHS, HCT, CHI were negatively correlated with flavonoids (**Supplementary Figure S4B**).

### qRT-PCR results

qRT-PCR was used to analyze the relative expression levels of genes encoding proteins involved in the biosynthesis of steroidal saponins and flavonoids (**Supplementary Figure S5**). The relative expression levels of the genes encoding farnesyl-diphosphate farnesyltransferase (*FDFT1*), Δ14-sterol reductase (*TM7SF2*), cholestenol Δ-isomerase (*EBP*) and cycloartenol synthase (*CAS1*) related to steroidal saponin biosynthesis showed a trend of first increasing and then decreasing across the four growth stages. The relative expression levels of the genes encoding flavonol synthase (*FLS*) and flavonoid 3’-monooxygenase (*CYP75B1*), which are closely related to flavonoid biosynthesis, showed a trend of first decreasing and then increasing across the four growth stages. While 2-hydroxyisoflavanone dehydratase (*HIDH*) gradually decreased, chalcone synthase (*CHS*) and chalcone isomerase (*CHI*) showed a trend of first increasing and then decreasing. The expression levels of these genes were consistent with the protein levels of corresponding enzymes (**Supplementary Tables S5**, **S6**).

## Discussion

Previous studies have shown that the contents of secondary metabolites varies considerably at different growth stages of *P. odoratum (* Wang and Yang, 2009; Wang and Xi, 2015). Steroids and flavonoids are among the key metabolites, as well as the main components of *P. odoratum* that exert pharmacological effects (Liu JR. et al., 2023; Liu XM. et al., 2023), so it is crucial to understand the accumulation of proteins and metabolites associated with a specific active components of *P. odoratum* extracts. In general, the accumulation metabolism of metabolites in plants is a complex process, and changes of metabolite abundance can be influenced by the plant growth state, genetic modifications and environmental conditions (Yang et al., 2018; Li YQ. et al., 2020; Xu et al., 2022). In this study, we synthesized the data of target metabolites in four growth stages of *P. odoratum* rhizomes and investigated the key metabolic pathways to reveal the metabolic activities related to the accumulation of steroidal saponins and flavonoids in *P. odoratum*.

The accumulation of metabolites in plants is characterized by spatiotemporal changes, which are closely related to plant growth, development and responses to external factors. In this study, a metabolomic analysis revealed 1,436 differential metabolites, which can be classified into 13 categories according to their structures. Primary metabolites, including amino acids, lipids, nucleic acids and carbohydrates, are essential for all life processes, providing both building blocks and energy for the growth and development of plants (Erb and Kliebenstein, 2020; Li SF. et al., 2020). By contrast, secondary metabolites are not directly involved in growth and development, but play an important role in improving the ability of plants to withstand various forms of stress and adapt to the changing environment (Bartwal et al., 2012; Faizal and Geelen, 2013; Divekar et al., 2022). Metabolomic heatmap and subcluster analyses showed that metabolites were accumulated in different modes in the rhizomes of *P. odoratum*, with the highest number of upregulated metabolites in spring, followed by winter. Among them, steroids were the most abundant in spring, including 14 spirostane saponins, 16 isospirostane saponins and 8 furostane saponins. In plants, steroidal saponins act as a line of defense against pathogen infection. When a plant is infected by a pathogen, the amount of precursor substances used for steroidal saponin synthesis will increase due to the upregulation of corresponding genes, resulting in an increase of the steroidal saponin content (Morrissey and Osbourn, 1999; Wittstock and Gershenzon, 2002). Steroidal saponins also protect plants from phytophagous animals or insects, as many of these compounds are cytotoxic, disrupting the cell membranes of blood cells and many other animal cells, as well as acting as toxins and digestive inhibitors that inhibit feeding by insects (Sparg et al., 2004; Massad and Hermy, 2012; Cavaiuolo et al., 2013). Flavonoids were the most abundant in winter, including 19 other flavonoids (14 homoisoflavanones), 12 flavones, 8 flavanones, 7 flavonols, 5 chalcones, 2 flavanonols, 2 isoflavones and 1 flavanol. Flavonoids act as antioxidants that are able to scavenge reactive oxygen species (ROS), thus protecting plants from damage caused by biotic and abiotic stresses, including cold stress, pathogen infection and insect feeding (Iwashina, 2003; Cavaiuolo et al., 2013). Flavonoids can also act as signaling molecules to attract pollinators and participate in growth hormone metabolism (Pourcel et al., 2007). Different growth stages significantly affected the metabolite composition of plants in this study, which was consistent with a previous study showing that the growth stage significantly affected the metabolite composition in *P. odoratum* rhizomes (Zhang et al., 2020). In order to clearly understand the mechanism of metabolite metabolism in *P. odoratum* during growth and development, it is necessary to monitor the activities of metabolite-related genes and enzymes during these periods.

As enzymes or regulatory factors, proteins are directly involved in metabolism and other cellular processes (Yang et al., 2014; Lecourieux et al., 2020). Therefore, proteomic analyses can reveal the regulatory mechanisms of important secondary metabolites in *P. odoratum*. Here, we identified 3,864 differentially expressed proteins (DEPs), which were categorized into different functional classes, such as general function prediction only, posttranslational modification, protein turnover, chaperones, translation, ribosomal structure and biogenesis, energy production and conversion, carbohydrate transport and metabolism, signal transduction mechanisms, intracellular trafficking, secretion, and vesicular transport, as well as secondary metabolites biosynthesis, transport and catabolism. These different functional classes of proteins may play important roles in plant growth, development and metabolic processes. KEGG pathway enrichment analysis of proteins in subcluster 5 showed that steroid and brassinosteroid biosynthesis, which are associated with steroidal saponins, had significant DEPs at different growth stages of *P. odoratum*. Steroidal saponins are spirostanol or furanosterol derivatives biosynthesized through a series of enzymatic modification starting from the molecular backbone of squalene. Firstly, 2,3-oxidosqualene is biosynthesized via the mevalonate (MVA) and 2-C-methyl-d-erythritol-4-phosphate (MEP) pathways. Then, cholesterol/β-sitosterol generated by a series of catalytic conversions of 2,3-oxidosqualene. Finally, steroidal saponins are formed through the hydroxylation, oxidation, and glycosylation of cholesterol or β-sitosterol at the C22, C16 and C26 positions (Liu et al., 2019; Cheng et al., 2023; Li et al., 2023), Proteins in the steroid biosynthesis pathway were highly expressed in spring, including FDFT1, TM7SF2, CAS1, DHCR7, 3BETAHSDD and TM7SF2. In addition, proteins related to plant-pathogen interactions were significantly enriched in subcluster 5, suggesting that pathogenic attack may also contribute to the increased synthesis of steroidal saponins in *P. odoratum*. KEGG pathway enrichment analysis of protein subcluster 1 revealed significant DEPs in pathways associated with the synthesis of flavonoids, isoflavonoids. flavones and flavonols. Flavonoids are mainly produced via the phenylalanine metabolic pathway, catalyzed by phenylalanine ammonia lyase (PAL), followed by cinnamic acid 4-hydroxylase (C4H) and 4-coumaroyl-CoA ligase (4CL), which respectively form *p*-coumaroyl-CoA, *p*-coumaroyl-CoA and malonyl-CoA. These compounds serve as starting substrates for the synthesis of naringenin chalcones by chalcone synthase (CHS). Then, naringenin chalcone is converted into naringenin by chalcone isomerase (CHI). Finally, naringenin is converted into isoflavones, flavonoids, flavonols, anthocyanins, etc., through the action of different enzymes (Wu et al., 2018; Wang J. et al., 2021). Proteins in the flavonoid biosynthesis pathway had high expression in winter, including HIDH, IF7MAT, PGT1, FLS, CYP75B1 and UGT73C6. This suggests that flavonoids may be accumulated in large quantities due to the high expression of the above proteins, whose expression corresponded to the observed differences in the accumulation of metabolites at different growth periods.

Multi-omics technology is a powerful tool offering a global overview of potential mechanisms that drive the synthesis of bioactive compounds in plants (Li et al., 2021; Wu et al., 2022). For example, Sun et al. combined metabolomic and proteomic analyses to study the changes of secondary metabolites in *Scutellaria baicalensis* at different growth stages (Sun et al., 2023). Liu et al. revealed the differences in aroma precursor synthesis in tobacco leaves at different fertility stages through metabolomics and proteomics (Liu et al., 2022). Correlation analysis of proteomic and metabolomic data showed that FDFT1, TM7SF2, DHCR7, CAS1 and 3BETAHSDD were positively correlated with the accumulation of steroidal saponins, while PGT, FLS, CYP75B1, HIDH, IF7MAT and UFT73C6 were positively correlated with the accumulation of flavonoids. Therefore, we concluded that these genes play important roles in the biosynthesis pathways of steroidal saponins and flavonoids, respectively. A number of enzymes related to the synthesis of steroidal saponins have been identified, but glycosyltransferases and CPY450s involved in the steroidal saponin biosynthesis pathway remain key areas requiring further research.

Significant differences in the abundance and composition of flavonoid and steroid-related metabolites were found in different growth stages of *P. odoratum*. In the future, the need for extraction of specific components can be met based on the selection of appropriate growth stages. Metabolite levels also demonstrated the importance of seasonal factors. Previous studies have shown that the content of steroidal saponins in *P. odoratum* rhizomes harvested at different times or in different geographic locations varied considerably, and that rational harvesting in spring and fall is conducive to obtaining a higher steroidal saponin content in *P. odoratum* (Liu et al., 2018). This was consistent with our metabolomic data. Overall, the integrated metabolomics and proteomic analysis provides us with an in-depth understanding of the mechanisms of metabolite transformation and protein expression in different growth stages of *P. odoratum*. By studying the correlation between metabolites and proteins, we can reveal important information about the growth, development and metabolic regulation of *P. odoratum*, thus providing a theoretical basis for further studies on the regulation of plant active ingredients. In addition, the different accumulation patterns of metabolites in *P. odoratum* rhizomes at different growth stages provide an important basis for the extraction of specific components.

## Conclusions

In this study, we used a combination of proteomics and metabolomics to thoroughly investigate the differences in protein expression and chemical composition of *P. odoratum* at different growth stages. In agreement with previous studies, steroids and flavonoids were identified as the main active components of *P. odoratum*, whereby metabolomic data showed that flavonoids showed the highest accumulation in winter, followed by spring. Consistently, the most significantly upregulated proteins were involved in the biosynthesis of flavonoids, isoflavonoids, flavones and flavonols. The accumulation of 56 flavonoid compounds was positively correlated with the protein expression of PGT, FLS, CYP75B1, HIDH, IF7MAT and UFT73C6. The highest accumulation of steroidal compounds was found in spring, followed by summer and autumn. The most significantly upregulated proteins were involved in steroid and brassinosteroid biosynthesis, while the accumulation of 38 steroidal saponins was positively correlated with the protein expression of FDFT1, TM7SF2, DHCR7, CAS1 and 3BETAHSDD. The upregulation of these key genes largely explains the significant increase in the accumulation of flavonoids and steroidal saponins. The present study provides theoretical guidance for future investigations of the anabolic pathways of steroidal compounds and flavonoids in *P. odoratum*. Moreover, it provides a useful reference for choosing the correct time to extract specific components from *P. odoratum*. This is of great significance for future research on raw material harvesting, processing and product development based on *P. odoratum*.

## Data Availability

The original contributions presented in the study are publicly available. This data can be found here: https://proteomecentral.proteomexchange.org/cgi/GetDataset?ID=PXD058887.
