# Supplementary material for: Integrated metabolomics and proteomics analysis reveals the accumulation mechanism of bioactive components in Polygonatum odoratum
Source: Front Plant Sci. 2024 Dec 20;15:1487613. doi: 10.3389/fpls.2024.1487613 (PMC11696735; doi:10.3389/fpls.2024.1487613)
Supplement: Supplementary file 1 [file Table1.docx]

Table S1 Primer sequences used for qRT-PCR

| Gene | Sense Primer | Anti-sense Primer |
| --- | --- | --- |
| Actin | AAGTTGCTGGAATCCACGAG | CTCATACGATCAGCAATACC |
| FDFT1 | GAATGGGTGCAGGAATGGCA | GCCAACAAGGCCTGCTACAT |
| TM7SF2 | TATCGCTGCAACGGTTTGCT | AGCAGCTCAAGCCCATTGTC |
| DHCR24-1 | GGATGGCTGATGCCACCAAA | ACCTCCATTTCGCGGTGAAC |
| EBP-2 | AAACCCTACCTCGCCTCCTC | CATGAGTCAGGCCCGTGAAG |
| CAS1 | AGGGAGGCAAGTTTGGGAGT | TCGTGCTCTGCTAGCCTGAT |
| CHS-4 | AGCGACCAAAGCCATCAAGG | ATGAAGCGCTTGGTGGTGAG |
| CHI-1 | TGGCAGGGTAGTCAGGATGG | GCAGCTGCACTCAGGACATT |
| FLS-1 | CCATGTTGCTCGAAGCCTGA | AAGGCCCTCCACCTCTTTGT |
| CYP75B1 | ATTACCGGGACGCTGATCCA | CTTCTCTCCCATCCGACCGT |
| HIDH-1 | TCACCTCCAAGGACGTCACA | GAGGTTGGCTTTGGATGCGA |

Table S2 Classification of differential metabolites.

| Index | Compounds | | Class |
| --- | --- | --- | --- |
| Zbqn002049 | | (2-carboxyethyl)-L-phenylalanine | Amino acids and derivatives |
| Zmmp002946 | | (3-(carboxyamino)-2-methylpropanoyl)phenylalanine | Amino acids and derivatives |
| Zbqn001288 | | (3-hydroxypropanoyl)-L-leucine | Amino acids and derivatives |
| Zmqp004530 | | (6-hydroxyhexahydro-1H-pyrrolizin-1-yl)methyl2-(acetoxymethyl)-2-hydroxy-4-methylpentanoate | Amino acids and derivatives |
| MWS0933 | | 1-Methylhistidine | Amino acids and derivatives |
| Zbqp001937 | | 2,6-diamino-7-methyl-5-oxooctanoic acid | Amino acids and derivatives |
| Zmmp002167 | | 2-((L-tyrosyl)oxy)-5-amino-5-oxopentanoic acid | Amino acids and derivatives |
| Zmmp000606 | | 2-Amino-4-hydroxy-3-methylpentanoic acid | Amino acids and derivatives |
| MWS04491 | | 3,4-Dehydro-DL-proline | Amino acids and derivatives |
| mws0886 | | 3,5-Diiodo-L-tyrosine | Amino acids and derivatives |
| Zbqp000676 | | 3-(triazan-2-yl)propyl L-prolinate | Amino acids and derivatives |
| MWS1039a | | 3-Amino-2-methylpropanoic acid | Amino acids and derivatives |
| MWS04559g | | 3-Hydroxy-L-phenylalanine* | Amino acids and derivatives |
| MWSmce190 | | 4-Hydroxy-L-Isoleucine | Amino acids and derivatives |
| Zbqp002641 | | 4-amino-2-(1-amino-3-(((S)-1-carboxyethyl)amino)-3-oxopropyl)-6-methyl-3-oxoheptanoic acid | Amino acids and derivatives |
| Zbqp002313 | | 4-amino-5-(butylamino)-5-oxopentanoic acid | Amino acids and derivatives |
| Zbqp003444 | | 4-amino-5-oxo-5-(pentylamino)pentanoic acid | Amino acids and derivatives |
| MWSprf117 | | 5-Hydroxy-DL-tryptophan(5-HTP) | Amino acids and derivatives |
| pme2566 | | 5-L-Glutamyl-L-Alanine | Amino acids and derivatives |
| mws0263 | | 5-Oxo-L-Proline* | Amino acids and derivatives |
| MWS0813 | | 5-Oxoproline* | Amino acids and derivatives |
| Lcsp001337 | | Ala-Ile-Ala | Amino acids and derivatives |
| Lcsp001369 | | Ala-Ile-Asp | Amino acids and derivatives |
| Lcsp001437 | | Ala-Leu-Glu | Amino acids and derivatives |
| pme3351 | | Allysine(6-Oxo DL-Norleucine) | Amino acids and derivatives |
| Zmdp000292 | | Arginine methyl ester* | Amino acids and derivatives |
| MWS201391 | | Asn-Ile | Amino acids and derivatives |
| MWS201449 | | Asn-Leu | Amino acids and derivatives |
| MWS201408 | | Asp-Glu | Amino acids and derivatives |
| Zbqp003309 | | Asp-Ile-Leu | Amino acids and derivatives |
| MWSmce706 | | Cyclo(Ala-Gly) | Amino acids and derivatives |
| MWStz083 | | Cyclo(D-Val-L-Pro) | Amino acids and derivatives |
| MWStz091 | | Cyclo(L-Ala-L-Pro) | Amino acids and derivatives |
| Lmhp001430 | | Cyclo(Pro-Glu) | Amino acids and derivatives |
| MWStz255 | | Cyclo(Pro-Phe) | Amino acids and derivatives |
| Lmrj002244 | | Cyclo(Pro-Pro) | Amino acids and derivatives |
| Lmrj002698 | | Cyclo(Pro-Val) | Amino acids and derivatives |
| Lmrj001341 | | Cyclo(Ser-Pro) | Amino acids and derivatives |
| Hmlp001371 | | Cyclo(Tyr-Ala) | Amino acids and derivatives |
| Lmrj002261 | | Cyclo(Val-Ala) | Amino acids and derivatives |
| ML10181668 | | Cycloleucine | Amino acids and derivatives |
| mws4532g | | D-Ornithine | Amino acids and derivatives |
| Rfmb318 | | D-Proline betaine | Amino acids and derivatives |
| MWS1926 | | DL-Leucine* | Amino acids and derivatives |
| mws0224 | | DL-Methionine | Amino acids and derivatives |
| MWS00275g | | DL-O-tyrosine | Amino acids and derivatives |
| Lcsn000385 | | Glu-Asn | Amino acids and derivatives |
| Lcsp001468 | | Glu-Ile-Glu | Amino acids and derivatives |
| Zmmp003258 | | Glu-Pro-Leu | Amino acids and derivatives |
| Zmmp002373 | | Glu-Thr-Leu | Amino acids and derivatives |
| pme1086 | | Glutathione reduced form | Amino acids and derivatives |
| Lcsp002329 | | Gly-Leu-Val | Amino acids and derivatives |
| Lcsp001899 | | Gly-Phe-Glu | Amino acids and derivatives |
| MWS20645 | | Gly-Tyr | Amino acids and derivatives |
| Lcsp000959 | | Gly-Val-Ala | Amino acids and derivatives |
| Lcsp002477 | | Gly-Val-Leu | Amino acids and derivatives |
| MWS4309 | | Glycyl-tryptophan | Amino acids and derivatives |
| MWS4296 | | Glycylphenylalanine* | Amino acids and derivatives |
| Lcsp003035 | | H-Leu-Trp-OH | Amino acids and derivatives |
| pme2853 | | Hexanoyl-L-glycine | Amino acids and derivatives |
| MWS04447 | | Homoproline | Amino acids and derivatives |
| MWS201424 | | Ile-Asn | Amino acids and derivatives |
| Lcsp002583 | | Ile-Glu-Val | Amino acids and derivatives |
| Zbqp003125 | | Ile-Phe | Amino acids and derivatives |
| Lcsp002647 | | Ile-Trp | Amino acids and derivatives |
| pme2074 | | Jasmonoyl-L-Isoleucine | Amino acids and derivatives |
| pme0128 | | L-Alanyl-L-Alanine | Amino acids and derivatives |
| mws4176 | | L-Alanyl-L-Phenylalanine | Amino acids and derivatives |
| mws5037 | | L-Alanyl-L-leucine | Amino acids and derivatives |
| pme2679 | | L-Allo-isoleucine | Amino acids and derivatives |
| mws0260 | | L-Arginine | Amino acids and derivatives |
| mws0001 | | L-Asparagine | Amino acids and derivatives |
| mws0219 | | L-Aspartic Acid | Amino acids and derivatives |
| pmb0464 | | L-Aspartic acid-O-diglucoside | Amino acids and derivatives |
| mws0629 | | L-Aspartyl-L-Phenylalanine | Amino acids and derivatives |
| pme0014 | | L-Glutamic acid | Amino acids and derivatives |
| pmb2857 | | L-Glutamic acid-O-glycoside | Amino acids and derivatives |
| pme0193 | | L-Glutamine | Amino acids and derivatives |
| pmb2855 | | L-Glutamine-O-glycoside | Amino acids and derivatives |
| Lcsp002225 | | L-Glutaminyl-L-tryptophan | Amino acids and derivatives |
| mws5042 | | L-Glycyl-L-phenylalanine* | Amino acids and derivatives |
| pme0124 | | L-Glycyl-L-proline | Amino acids and derivatives |
| mws0254 | | L-Histidine | Amino acids and derivatives |
| pme2890 | | L-Homocystine | Amino acids and derivatives |
| Lmrj002087 | | L-Isoleucyl-L-Aspartate | Amino acids and derivatives |
| Lmhp002031 | | L-Leucyl-L-Leucine | Amino acids and derivatives |
| mws5035 | | L-Leucyl-L-phenylalanine | Amino acids and derivatives |
| pme0026 | | L-Lysine | Amino acids and derivatives |
| pmb0962 | | L-Lysine-Butanoic Acid | Amino acids and derivatives |
| pme2617 | | L-Methionine Sulfoxide | Amino acids and derivatives |
| MWS3047 | | L-Methionine Sulfoximine | Amino acids and derivatives |
| pme1419 | | L-Methionine methyl ester | Amino acids and derivatives |
| mws1587 | | L-Norleucine* | Amino acids and derivatives |
| pme2527 | | L-Ornithine | Amino acids and derivatives |
| pme0021 | | L-Phenylalanine | Amino acids and derivatives |
| pme0006 | | L-Proline | Amino acids and derivatives |
| Lmhp001461 | | L-Prolyl-L-Leucine | Amino acids and derivatives |
| Lmhp001732 | | L-Prolyl-L-Phenylalanine | Amino acids and derivatives |
| pme1712 | | L-Saccharopine | Amino acids and derivatives |
| pme0010 | | L-Serine | Amino acids and derivatives |
| Lmrj001698 | | L-Seryl-L-Isoleucine | Amino acids and derivatives |
| MWS1771 | | L-Tyrosine methyl ester | Amino acids and derivatives |
| mws0250 | | L-Tyrosine* | Amino acids and derivatives |
| mws0256 | | L-Valine | Amino acids and derivatives |
| Lmhp001670 | | L-Valyl-L-Leucine | Amino acids and derivatives |
| Lmhp002001 | | L-Valyl-L-Phenylalanine | Amino acids and derivatives |
| Zmzn000113 | | L-threo-3-Methylaspartate | Amino acids and derivatives |
| Zmdp002216 | | L-γ-Glutamyl-L-leucine | Amino acids and derivatives |
| MWS201437 | | Leu-Asp | Amino acids and derivatives |
| Lcsp003066 | | Leu-Glu-Ile | Amino acids and derivatives |
| Lcsp001901 | | Leu-Ile-Glu | Amino acids and derivatives |
| Lcsp001416 | | Leu-Thr-Ala | Amino acids and derivatives |
| MWS201412 | | Lys-Asp | Amino acids and derivatives |
| MWS201436 | | Lys-Thr | Amino acids and derivatives |
| MWSmce585 | | Methyl 3-aminopropanoate | Amino acids and derivatives |
| pme0109 | | Methyldopa | Amino acids and derivatives |
| MWS04412 | | N(6),N(6)-Dimethyl-L-lysine | Amino acids and derivatives |
| MWS5164 | | N,N'-Dimethylarginine;SDMA* | Amino acids and derivatives |
| Wayn000369 | | N-(1-Deoxy-1-fructosyl)Alanine | Amino acids and derivatives |
| Wayp001214 | | N-(1-Deoxy-1-fructosyl)Arginine | Amino acids and derivatives |
| Wayn000341 | | N-(1-Deoxy-1-fructosyl)Asparagine | Amino acids and derivatives |
| Wayn000815 | | N-(1-Deoxy-1-fructosyl)Glutamic acid | Amino acids and derivatives |
| Wayp000599 | | N-(1-Deoxy-1-fructosyl)Histidine | Amino acids and derivatives |
| Wayn001257 | | N-(1-Deoxy-1-fructosyl)Leucine | Amino acids and derivatives |
| Wayn002083 | | N-(1-Deoxy-1-fructosyl)Phenylalanine | Amino acids and derivatives |
| Wayn000421 | | N-(1-Deoxy-1-fructosyl)Proline | Amino acids and derivatives |
| Wayn002636 | | N-(1-Deoxy-1-fructosyl)Tryptophan | Amino acids and derivatives |
| Wayp001104 | | N-(1-Deoxy-1-fructosyl)Tyrosine | Amino acids and derivatives |
| Wayp001024 | | N-(1-Deoxy-1-fructosyl)Valine | Amino acids and derivatives |
| ZbWp000360 | | N-(1-deoxy-1-xylosyl)valine | Amino acids and derivatives |
| mws0124 | | N-(3-Indolylacetyl)-L-alanine | Amino acids and derivatives |
| Zbqn004590 | | N-(acetyl)phenylalanine | Amino acids and derivatives |
| Zbqn004234 | | N-(malonyl)phenylalanine | Amino acids and derivatives |
| Zbqn004682 | | N-(malonyl)tryptophan | Amino acids and derivatives |
| Zbqn000955 | | N-Acetoxy-glucosyl-alanine | Amino acids and derivatives |
| pme0170 | | N-Acetyl-L-Arginine | Amino acids and derivatives |
| pme2559 | | N-Acetyl-L-Aspartic Acid | Amino acids and derivatives |
| pme0137 | | N-Acetyl-L-Glutamine | Amino acids and derivatives |
| pme0253 | | N-Acetyl-L-leucine | Amino acids and derivatives |
| Zmgn002106 | | N-Acetyl-L-phenylalanine | Amino acids and derivatives |
| pme3382 | | N-Acetyl-L-threonine | Amino acids and derivatives |
| mws0520 | | N-Acetyl-L-tyrosine | Amino acids and derivatives |
| ZbWn002006 | | N-Acetyl-L-valine | Amino acids and derivatives |
| MWSslk042 | | N-Ethylmaleimide (NEM) | Amino acids and derivatives |
| ZbWp003008 | | N-Ethylphenylalanine | Amino acids and derivatives |
| Wcdn000881 | | N-Fructosyl Pyroglutamate | Amino acids and derivatives |
| mws0736 | | N-Glycyl-L-leucine | Amino acids and derivatives |
| Yalp000545 | | N-Methyl-L-proline | Amino acids and derivatives |
| Zmjp000182 | | N-Monomethyl-L-arginine* | Amino acids and derivatives |
| MWS3166 | | N-Palmitoylglycine | Amino acids and derivatives |
| mws0712 | | N-Propionylglycine | Amino acids and derivatives |
| MWS2413 | | N-acetyl-beta-alanine | Amino acids and derivatives |
| Zbqn003748 | | N-malonylleucine | Amino acids and derivatives |
| ZbWp002423 | | N-methylphenylalanine | Amino acids and derivatives |
| Zbqn002318 | | N-succinyl-proline | Amino acids and derivatives |
| pme0122 | | N6-Acetyl-L-lysine | Amino acids and derivatives |
| NK10251888 | | NG,NG-Dimethyl-L-arginine* | Amino acids and derivatives |
| MWS201054 | | O-Acetyl-L-homoserine | Amino acids and derivatives |
| mws1050 | | O-Acetylserine | Amino acids and derivatives |
| MWS20633g | | O-phosphate-L-tyrosine | Amino acids and derivatives |
| MWS0132 | | Oxiglutatione | Amino acids and derivatives |
| Zbqp002379 | | Phe-Gln | Amino acids and derivatives |
| MWS201483 | | Phe-Hyp | Amino acids and derivatives |
| MWS201458 | | Phe-Ile | Amino acids and derivatives |
| MWS201478 | | Phe-Ser | Amino acids and derivatives |
| MWS201442 | | Phe-Thr | Amino acids and derivatives |
| MWS201414 | | Pro-Asn | Amino acids and derivatives |
| mws4516 | | Pyroglutamic acid | Amino acids and derivatives |
| pme1286 | | S-(5'-Adenosy)-L-homocysteine | Amino acids and derivatives |
| mws0582 | | S-(Methyl)glutathione | Amino acids and derivatives |
| Zmdp000972 | | S-Methyl-L-cysteine | Amino acids and derivatives |
| Zmgn001039 | | S-Ribosyl-L-homocysteine | Amino acids and derivatives |
| Lcsp001896 | | Ser-Gly-Phe | Amino acids and derivatives |
| Lcsp003090 | | Ser-Leu-Leu | Amino acids and derivatives |
| MWS201443 | | Ser-Lys | Amino acids and derivatives |
| MWS201381 | | Ser-Trp | Amino acids and derivatives |
| Lcsp001977 | | Thr-Glu-Ile | Amino acids and derivatives |
| Lcsn001170 | | Thr-Leu | Amino acids and derivatives |
| Lcsp002261 | | Thr-Trp | Amino acids and derivatives |
| MWS201394 | | Thr-Tyr | Amino acids and derivatives |
| mws0216 | | Trans-4-Hydroxy-L-proline | Amino acids and derivatives |
| Zmzp000145 | | Trimethyllysine | Amino acids and derivatives |
| MWS201445 | | Val-Pro | Amino acids and derivatives |
| MWS201400 | | Val-Trp | Amino acids and derivatives |
| Zbqp003189 | | Val-Val | Amino acids and derivatives |
| ZbWp000749 | | ethyl-L-arginine | Amino acids and derivatives |
| Zbqp002414 | | h-gamma-Glu-leu-oh | Amino acids and derivatives |
| Zbqn001255 | | propyl-L-alanine | Amino acids and derivatives |
| Zmmp003443 | | γ-Glu-Trp | Amino acids and derivatives |
| Zmdp001647 | | γ-Glutamyl-L-valine | Amino acids and derivatives |
| Wccp002543 | | γ-Glutamylphenylalanine | Amino acids and derivatives |
| Zmdp001857 | | γ-Glutamyltyrosine | Amino acids and derivatives |
| Zmdp001928 | | γ-L-Glutamyl-S-(trans-1-propenyl)-L-cysteine | Amino acids and derivatives |
| MWSHC2028 | | (1S,3S)-1-Methyl-1,2,3,4-tetrahydro-β-carboline-3-carboxylic acid | Alkaloids |
| Wbjp002330 | | 1,3-dihydroxy-2,3,3a,4-tetrahydropyrrolo[2,1-b]quinazolin-9(1H)-one | Alkaloids |
| Zmsp000237 | | 1,4-Dideoxy-1,4-imino-(2-O-β-D-glucosyl)-D-arabinitol | Alkaloids |
| pmp001187 | | 1-(Dihydroxyphenyl)-N2,N3-bis(4-hydroxyphenethyl)-(5-8)-dimethoxy-1,2dihydronaphthalene-2,3-dicarboxamide | Alkaloids |
| Zmsp000758 | | 1-(Hydroxymethyl)hexahydro-1h-pyrrolizin-2-ol | Alkaloids |
| Hmcp003783 | | 1-Acetyl-β-carboline | Alkaloids |
| pme3200 | | 1-Methylguanidine | Alkaloids |
| Wbmp004804 | | 1-O-Demethyltricornine | Alkaloids |
| Yamp002097 | | 1-[2-(Furan-2-yl)-2-oxoethyl]pyrrolidin-2-One | Alkaloids |
| pmb1912 | | 10-Formyltetrahydrofolic Acid | Alkaloids |
| Wbmp010544 | | 10-Hydroxymethyllycaconitine | Alkaloids |
| Lmqp002340 | | 13,13α-Didehydro-9,10-dimethoxy-2,3-(methylenedioxy)-berbine | Alkaloids |
| Qmhp090808 | | 18-Demethylparaensidimerin C | Alkaloids |
| MWSmce625 | | 2,3,5,6-Tetramethylpyrazine; Ligustrazine | Alkaloids |
| NK10246260 | | 2,4-Dihydroxyquinoline | Alkaloids |
| Hmtn001358 | | 2-(D-Glucosyloxy)-4-hydroxybenzeneacetonitrile | Alkaloids |
| Lmtn001630 | | 2-Glucosyloxy-2-phenylacetic acid amide | Alkaloids |
| MWSslk106 | | 2-Phenylethylamine | Alkaloids |
| MWSmce460 | | 2-Piperidone | Alkaloids |
| Lcsp001671 | | 2-oxindole-3-acetic acid | Alkaloids |
| pmp000446 | | 22-O-Demethyl-22-O-glucosyl isocorynoxeine | Alkaloids |
| Lmlp001118 | | 3'-Glucosyl-6,7-dihydroxy-N-methyl-benzyltetrahydroisoquinoline | Alkaloids |
| Hmjp005556 | | 3-(4-Hydroxyphenyl)-N-[2-(4-hydroxyphenyl)ethyl]-2-propenamide* | Alkaloids |
| pme1738 | | 3-Carbamyl-1-methylpyridinium;(1-Methylnicotinamide) | Alkaloids |
| MWS1777 | | 3-Chloroaniline | Alkaloids |
| pmn001692 | | 3-O-Acetylhamayne | Alkaloids |
| Yacp000453 | | 3-hydroxy-1-methylpyrrolidin-2-one* | Alkaloids |
| Zblp001009 | | 3-pyridine-methanol-O-β-D-glucopyranosyl | Alkaloids |
| Hmtp000776 | | 4,5,6-Trihydroxy-2-cyclohexen-1-ylideneacetonitrile | Alkaloids |
| MA10074217 | | 4-Hydroxyquinoline | Alkaloids |
| Lamp000484 | | 4-Methylazetidine-2-Carboxylic acid* | Alkaloids |
| pma3649 | | 5-Aminolevulinic Acid | Alkaloids |
| MWStz073 | | 5-Hydroxy-2-pyrrolidinone | Alkaloids |
| Lmlp002994 | | 5-Hydroxyquinoline* | Alkaloids |
| pmp001198 | | 6-Deoxyfagomine | Alkaloids |
| Wbmp003594 | | 6-acetyldelpheline | Alkaloids |
| Lssp210093 | | 7-hydroxy-N-trans-ferulic tyramine | Alkaloids |
| Lajp006790 | | 8-Methoxy-4-methylquinolin-2(1H)-one | Alkaloids |
| Wccp005403 | | Acetyl-Caranine* | Alkaloids |
| pmb0501 | | Agmatine | Alkaloids |
| YC512112 | | Anabasine-glucoside | Alkaloids |
| MWSmce331 | | Azetidine-2-carboxylic acid | Alkaloids |
| pmb0069 | | Benzamide | Alkaloids |
| MWSmce548 | | Betaine | Alkaloids |
| pme1841 | | Cadaverine | Alkaloids |
| Wbjp004916 | | Cephalanthrin A | Alkaloids |
| pmb0484 | | Choline | Alkaloids |
| Zahp006974 | | Cinnamoyltyramine* | Alkaloids |
| pmb0891 | | Cis-Zeatin-7-N-glucoside | Alkaloids |
| Hmcp002354 | | Crenatine | Alkaloids |
| Qmgp102024 | | Cyclopamine | Alkaloids |
| mws1346 | | DL-2-Aminoadipic acid | Alkaloids |
| MWStz300 | | Dehydrocrenatidine | Alkaloids |
| Lmgp002501 | | Dhurrin-6'-O-Glucoside | Alkaloids |
| pme2433 | | Diethanolamine | Alkaloids |
| Wbmp004067 | | Dihydroberberine* | Alkaloids |
| Lmmp001410 | | Dihydrocaffeoylputrescine | Alkaloids |
| WaJp002519 | | Doryafranin Glucoside | Alkaloids |
| Lmlp004754 | | Ehretioside B | Alkaloids |
| MWSmce204 | | Epiberberine | Alkaloids |
| Xmzn007677 | | Ergotamine | Alkaloids |
| MWStz038 | | Grossamide* | Alkaloids |
| Wbjp004911 | | Hecogenone-rhamnose | Alkaloids |
| Wcdp010864 | | Hexadecanamide | Alkaloids |
| pme2122 | | Histamine | Alkaloids |
| MWSmce448 | | Imidazol-1-yl-acetic acid | Alkaloids |
| mws0103 | | Indole-3-carboxaldehyde | Alkaloids |
| mws1417 | | Indole-3-carboxylic acid* | Alkaloids |
| Lmrn003201 | | Indole-3-lactic acid | Alkaloids |
| mws0102 | | Indole-5-carboxylic acid* | Alkaloids |
| MWSmce542 | | Indoximod | Alkaloids |
| Qmgp102028 | | Isorubijervosine | Alkaloids |
| Hmdp005429 | | Jatrorrhizine | Alkaloids |
| Lmcp000282 | | L-Carnitine | Alkaloids |
| MWSmce340 | | L-Praziquanamine | Alkaloids |
| pme1002 | | L-Tyramine | Alkaloids |
| MWSslk177 | | Lithospermoside | Alkaloids |
| Lskp211279 | | Longitubanine A | Alkaloids |
| mws1383 | | Lumichrome | Alkaloids |
| Hmgp006095 | | Lyciumamide C | Alkaloids |
| Wbsp007290 | | Lycoposerramine E | Alkaloids |
| MWSmce582 | | Methyl L-pyroglutamate | Alkaloids |
| Hmyp002656 | | Methyl dioxindole-3-acetate | Alkaloids |
| pmb0492 | | N',N'',N'''-p-Coumaroyl-cinnamoyl-caffeoyl spermidine | Alkaloids |
| Wayn000370 | | N-(1-Deoxy-1-fructosyl)Piperidic acid | Alkaloids |
| Zahp014087 | | N-(12-methyltetradecyl)propionamide | Alkaloids |
| MWStz070 | | N-(2-Hydroxy-4-methoxyphenyl)acetamide | Alkaloids |
| pmn001727 | | N-(4-O-(Glucosyl)-E-feruloyl)-tyramine | Alkaloids |
| Wagp007639 | | N-(4-hydroxyphenethyl)cinnamamide* | Alkaloids |
| Wagp001741 | | N-(gamma-L-glutamyl)tyramine O-glucoside | Alkaloids |
| mws0677 | | N-Acetyl-5-hydroxytryptamine | Alkaloids |
| MWS3020 | | N-Acetylcadaverine | Alkaloids |
| Zmtn001624 | | N-Acetylisatin | Alkaloids |
| pme2693 | | N-Acetylputrescine | Alkaloids |
| pmp001287 | | N-Benzylmethylene isomethylamine | Alkaloids |
| Zdcp003644 | | N-Caffeoylputrescine | Alkaloids |
| Hmjp006104 | | N-Cis-Feruloyl-3'-O-methyldopamine | Alkaloids |
| MWStz221 | | N-Cis-Feruloyltyramine* | Alkaloids |
| Qmjp110218 | | N-Feruloyl dopamine* | Alkaloids |
| HJAP050 | | N-Feruloyl-3-methoxytyramine* | Alkaloids |
| Wafp003720 | | N-Feruloyloctopamine glucoside | Alkaloids |
| Wafp004987 | | N-Feruloylphenylacetamide | Alkaloids |
| Wafp003258 | | N-Feruloylphenylacetamide glucoside | Alkaloids |
| Lmlp003161 | | N-Feruloylputrescine | Alkaloids |
| Wagp001908 | | N-Feruloylputrescine N-glucoside | Alkaloids |
| Wafp004641 | | N-Feruloyltyramine 4-glucoside | Alkaloids |
| mws1433 | | N-Feruloyltyramine; Moupinamide* | Alkaloids |
| Zahp011321 | | N-Isobutyl Decanamide | Alkaloids |
| Qmdp090606 | | N-Methyltetrahydropalmatine | Alkaloids |
| mws0620 | | N-Methyltryptamine | Alkaloids |
| mws0983 | | N-Oleoylethanolamine | Alkaloids |
| Zahp005843 | | N-Trans-Sinapoyltyramine | Alkaloids |
| Hmjp005089 | | N-[2-(3,4-Dihydroxyphenyl)-2-hydroxyethyl]-3-(4-methoxyphenyl)-2-propenamide* | Alkaloids |
| Smcp000882 | | N-benzoyl-2-aminoethyl-β-D-glucopyranoside | Alkaloids |
| HJKP000649 | | N-benzylformamide* | Alkaloids |
| pmb2893 | | N-p-Coumaroylhydroxyagmatine | Alkaloids |
| Zbsp007466 | | N-trans-cinnamoylphydroxyphenylethylamine* | Alkaloids |
| Lssp210092 | | N-trans-ferulic tyramine* | Alkaloids |
| Zblp005777 | | N-trans-ferulicacidacylp-hydroxyphenylethylamine* | Alkaloids |
| Lsjp211313 | | N-trans-feruloyl-3-methoxytyramine* | Alkaloids |
| pma0702 | | N1,N8-Bis(sinapoyl)spermidine | Alkaloids |
| Lshp011101 | | Narciclasine 4-Glucopyranoside | Alkaloids |
| mws1375 | | Nicotianamine | Alkaloids |
| MWSslk108 | | O-Acetyl-L-carnitine | Alkaloids |
| mws0704 | | O-Phosphorylethanolamine | Alkaloids |
| Zahp012577 | | Octadecadienamide | Alkaloids |
| Lskp211278 | | Oxypinnatanine A | Alkaloids |
| MWSmce078 | | Palmatine | Alkaloids |
| mws1038 | | Pantetheine | Alkaloids |
| Lmcp002933 | | Pilosine | Alkaloids |
| pmp000148 | | Pingbeimine B | Alkaloids |
| Ysjp000315 | | Pipecolic acid | Alkaloids |
| pmb0782 | | Piperidine | Alkaloids |
| Hmdp000626 | | Polygonatine A | Alkaloids |
| MWS20160 | | Prunasin | Alkaloids |
| MWS3270 | | Quinolinic Acid | Alkaloids |
| Zbzp000519 | | Retronecine | Alkaloids |
| Qmgp102027 | | Rubivirine | Alkaloids |
| Lmqp002330 | | Sinomendine* | Alkaloids |
| mws0017 | | Spermidine | Alkaloids |
| HJN011 | | Tataramide A | Alkaloids |
| Wbjp006632 | | Tribulusamide A* | Alkaloids |
| pme2268 | | Trigonelline | Alkaloids |
| mws0005 | | Tryptamine | Alkaloids |
| Zmbp000490 | | Valerine | Alkaloids |
| Qmgp101917 | | Veraflorizine | Alkaloids |
| Qmgp102013 | | Veramanine | Alkaloids |
| Qmgp102117 | | Veratraline B | Alkaloids |
| Qmgp102132 | | Vertaline B | Alkaloids |
| Cmyp004481 | | Yanhusuine | Alkaloids |
| Lmxp000939 | | Zarzissine | Alkaloids |
| Qmgp102035 | | Zygadenine | Alkaloids |
| Hahp000801 | | alanine betaine | Alkaloids |
| Zmwp005562 | | cis-N-p-Coumaroyltyramine* | Alkaloids |
| Zblp005760 | | dihydro-N-feruloyltyramine | Alkaloids |
| Lahp002273 | | furan-2-carbohydrazide | Alkaloids |
| Wbjp001169 | | o-Carboxy-5-hydroxytryptamine | Alkaloids |
| pmb0508 | | p-Coumaroylagmatine | Alkaloids |
| pmb0490 | | p-Coumaroylputrescine | Alkaloids |
| Wbjp005065 | | p-Coumaroyltyramine* | Alkaloids |
| Wbjp002617 | | peganumal A-glucose | Alkaloids |
| Wbjp001317 | | vasicinone | Alkaloids |
| Smnp004333 | | 2''-O-Feruloyl aloesin | Flavonoids |
| Zapp009018 | | 2',6'-dimethoxy-4,4'-di-hydroxychalcone | Flavonoids |
| Hmdp001950 | | 2',7-Dihydroxy-3',4'-dimethoxyisoflavan | Flavonoids |
| Xmgp006913 | | 2,4,2',4'-tetrahydroxy-3'-prenylchalcone | Flavonoids |
| Zjmp102048 | | 2,5,7-trihydroxy-6,8-dimethyl-3-(3',4'-methylenedioxybenzyl)chroman-4-one* | Flavonoids |
| Zjmp102043 | | 2,5,7-trihydroxy-6,8-dimethyl-3-(4'-methoxybenzyl)chroman-4-one | Flavonoids |
| Hmhp006479 | | 2-(2,4-dihydroxybenzyl)-6,8-dihydroxy-7-methyl-3,4-dihydronaphthalen-1(2H)-one | Flavonoids |
| Lmtp003948 | | 3',5',5,7-Tetrahydroxy-4'-methoxyflavanone-3'-O-glucoside | Flavonoids |
| Lmsp004301 | | 3',5,5',7-Tetrahydroxyflavanone-7-O-glucoside* | Flavonoids |
| Yphn011804 | | 3'-O-Methyldiplacol | Flavonoids |
| Jmyn006479 | | 3,5,7-Trihydroxy-6,8-dimethyl-3-(4'-hydroxybenzyl)-chroman-4-one (Polygonatone C) | Flavonoids |
| mws0914 | | 3,5,7-Trihydroxyflavanone (Pinobanksin)* | Flavonoids |
| Zjhp090634 | | 3-(3',4'-dihydroxybenzylidene)-5,7-dihydroxy-6,8-dimethylchroman-4-one | Flavonoids |
| Lamp007163 | | 4'-Demethyleucomin glucoside* | Flavonoids |
| Lamp009984 | | 4'-Demethyleucomin* | Flavonoids |
| pmc1990 | | 4'-Hydroxy-5,7-dimethoxyflavanone | Flavonoids |
| MWSslk146 | | 4'-O-Glucosylvitexin | Flavonoids |
| Lamp009532 | | 4'-demethyl-3,9-dihydroeucomin | Flavonoids |
| pmn001477 | | 4-C-Glucose-1,3,6-trihydroxy-7-methoxyxanthone | Flavonoids |
| pmp000172 | | 5,2'-Dihydroxy-7,8-dimethoxyflavone glycoside | Flavonoids |
| Lajp005753 | | 5,3'-dihydroxy-6,7,4'-trimethoxyflavone-8-O-β-D-glucoside | Flavonoids |
| Zmhn003257 | | 5,7,2'-Trihydroxy-8-methoxyflavone* | Flavonoids |
| Hajp007218 | | 5,7,3',5'-tetrahydroxy-6-methylfavanone* | Flavonoids |
| Hmhn007438 | | 5,7-Dihydroxy-3-(4-Hydroxybenzyl)-4-Chromanone | Flavonoids |
| Zjhp090618 | | 5,7-Dihydroxy-6,8-dimethyl-3-(2',4'-hydroxybenzyl)chroman-4-One* | Flavonoids |
| Jmyp005575 | | 5,7-Dihydroxy-6,8-dimethyl-3-(3'-hydroxy-4'-methoxybenzyl)-chroma4-one* | Flavonoids |
| Jmyn006493 | | 5,7-Dihydroxy-6,8-dimethyl-3-(4'-hydroxybenzyl)-chromone-4-one | Flavonoids |
| Zjhn090628 | | 5,7-Dihydroxy-8-methyl-3-(2',4'-dihydroxybenzyl)-chroman-4-one | Flavonoids |
| Zjhp090603 | | 5,7-dihydroxy-3-(4'-hydroxybenzyl)-chroman-4-one | Flavonoids |
| Zjhp090636 | | 5,7-dihydroxy-3-(4'-hydroxybenzylidene)-chroman-4-one* | Flavonoids |
| Zjhp090611 | | 5,7-dihydroxy-3-[(4-methoxyphenyl)methyl]-6-methyl-2,3-dihydrochromen-4-one* | Flavonoids |
| Zjhp090620 | | 5,7-dihydroxy-6,8-dimethyl-3(R,S)-(3'-hydroxy-4'-methoxybenzyl)-chroman-4-one* | Flavonoids |
| Zjmp102041 | | 5,7-dihydroxy-6,8-dimethyl-3-(4'-hydroxy-3'-methoxybenzyl)chroman-4-one* | Flavonoids |
| Zjhp090635 | | 5,7-dihydroxy-6,8-dimethyl-3-(4'-methoxybenzyl)-chroman-4-one* | Flavonoids |
| Zjhp090633 | | 5,7-dihydroxy-6-methoxyl-8-methyl-3-(2',4'-dihydroxybenzyl)-chroman-4-one | Flavonoids |
| Lalp007415 | | 5,7-dihydroxy-6-methyl-3-(4'-hydroxybenzyl)-4-chromanone* | Flavonoids |
| Zjhp090619 | | 5,7-dihydroxy-8-methyl-3-(2'-hydroxy-4'-methoxybenzyl)-chroman-4-one | Flavonoids |
| Zjhp090605 | | 5,7-dihydroxy-8-methyl-3-(4'-hydroxybenzyl)-chroman-4-one | Flavonoids |
| Zjhp090606 | | 5,7-dihydroxyl-6-methyl-3-(4'-hydroxylbenzyl)-chroman-4-one* | Flavonoids |
| Lajp005898 | | 5,8,3'-trihydroxy-6,7,4'-trimethoxyflavone-8-O-β-D-glucoside | Flavonoids |
| Hmmp004218 | | 5-Hydroxy-3-(2-hydroxybenzyl)-7-methoxychroman-4-one | Flavonoids |
| Zjhp090617 | | 5-hydroxy-3-(2-hydroxy-4-methoxybenzyl)-7-methoxychroman-4-one | Flavonoids |
| Zjhp090623 | | 5-hydroxy-7-methoxy-6,8-dimethyl-3-(2'-hydroxy-4'-methoxybenzyl)-chroman-4-one | Flavonoids |
| Wagp009755 | | 6'-Hydroxy-4,2',3',4'-Tetramethoxychalcone | Flavonoids |
| Zmhp003514 | | 6,7,8-Tetrahydroxy-5-methoxyflavone | Flavonoids |
| Hmhp006830 | | 6,8-Dihydroxy-2-(2-hydroxy-4-methoxybenzyl)-7-methyl-3,4-dihydronaphthalen-1(2H)-one* | Flavonoids |
| Hmhp006887 | | 6,8-Dihydroxy-2-(4-hydroxybenzyl)-5,7-dimethyl-3,4-dihydronaphthalen-1(2H)-one | Flavonoids |
| Lmjp003655 | | 6-C-MethylKaempferol-3-glucoside | Flavonoids |
| Yphn012690 | | 6-Geranylnaringenin; Mimulone; Bonannione A | Flavonoids |
| Lmsn004132 | | 6-Hydroxy-4',5,7-trimethoxyflavanone; Hamiltone A | Flavonoids |
| Lalp010122 | | 7,3'-Dihydroxy-4'-methoxy-8-methylflavan* | Flavonoids |
| Hagn003556 | | 7-Benzyloxy-5-hydroxy-3',4'-methylenedioxyflavonoid | Flavonoids |
| Lmhn004960 | | 7-O-Galloyltricetiflavan | Flavonoids |
| Cmsp008121 | | 7-O-Methylnaringenin | Flavonoids |
| Zjhp090625 | | 7-O-β-D-glucopyranoside-5-hydroxy-3-(4'-hydroxybenzylidene)-chroman-4-one* | Flavonoids |
| Smdp007779 | | 8-Demethylfarrerol | Flavonoids |
| pmp000573 | | Acacetin-7-O-galactoside | Flavonoids |
| pmp001288 | | Acacetin-7-O-rutinoside (Linarin) | Flavonoids |
| Lmtp002474 | | Apigenin-6-C-(2''-glucosyl)arabinoside | Flavonoids |
| pmp000237 | | Apigenin-6-C-(2''-glucuronyl)xyloside | Flavonoids |
| Zbjp003463 | | Apigenin-6-C-(2''-xylosyl)glucoside | Flavonoids |
| mws1434 | | Apigenin-6-C-glucoside (Isovitexin) | Flavonoids |
| pmb0626 | | Apigenin-6-C-glucoside-7-O-Sophoroside | Flavonoids |
| ZBN0018 | | Apigenin-7-O-(2''-O-glucuronyl)glucuronide-4'-O-glucuronide | Flavonoids |
| Lmpp003930 | | Apigenin-7-O-(6''-p-Coumaryl)glucoside | Flavonoids |
| mws0047 | | Apigenin-7-O-neohesperidoside (Rhoifolin) | Flavonoids |
| pme0368 | | Apigenin-7-O-rutinoside (Isorhoifolin)* | Flavonoids |
| Lmtp002642 | | Apigenin-8-C-(2''-glucosyl)arabinoside | Flavonoids |
| Lmhn005110 | | Aracarpene 1 | Flavonoids |
| Lmhn004976 | | Aracarpene 2* | Flavonoids |
| MWSHY0111 | | Calycosin-7-O-glucoside | Flavonoids |
| Zmhn001036 | | Choerospondin | Flavonoids |
| Zbrn006627 | | Chrysoeriol-5-O-glucoside | Flavonoids |
| Zbrn008158 | | Chrysoeriol; 5,7,4'-Trihydroxy-3'-Methoxyflavone* | Flavonoids |
| HJN055 | | Dihydrocharcone-4'-O-glucoside | Flavonoids |
| Lmlp005236 | | Dihydrokaempferol-3-O-glucoside* | Flavonoids |
| Lmmn004625 | | Dihydrokaempferol-7-O-glucoside | Flavonoids |
| mws0058 | | Diosmetin (5,7,3'-Trihydroxy-4'-methoxyflavone)* | Flavonoids |
| Hmhn006723 | | Disporopsin | Flavonoids |
| Zjhp090607 | | Disporopsin/5,7-dihydroxy-3-(2',4'-dihydroxybenzyl)-chroman-4-one | Flavonoids |
| HJAP020 | | Eriodictyol-5,3'-Di-O-glucoside | Flavonoids |
| Lmyn004037 | | Eriodictyol-7-O-(6''-O-galloyl)glucoside | Flavonoids |
| Lmdn003665 | | Farrerol-5,7-di-O-glucoside | Flavonoids |
| MWSN0022 | | Flavokawain C | Flavonoids |
| Lmmp000897 | | Gallocatechin-(4α→8)-gallocatechin | Flavonoids |
| Lmgp004474 | | Genistein-7-O-galactoside-rhamnose* | Flavonoids |
| Zbgp004340 | | Genistein-8-C-glucoside-O-apiosyl | Flavonoids |
| Xmyn008071 | | Gnetifolin B* | Flavonoids |
| Lmmp003271 | | Gossypetin-8-O-glucoside | Flavonoids |
| Zbhp004510 | | Hesperetin-3'-O-glucoside* | Flavonoids |
| pmb0645 | | Hesperetin-6-C-glucoside-7-O-glucoside | Flavonoids |
| Lmzp002365 | | Hesperetin-7-O-glucoside* | Flavonoids |
| pmb0618 | | Hesperetin-8-C-glucoside-3'-O-glucoside | Flavonoids |
| MWSHY0069 | | Hispidulin (5,7,4'-Trihydroxy-6-methoxyflavone)* | Flavonoids |
| Lmnp002584 | | Hispidulin-8-C-(2''-O-glucosyl)glucoside | Flavonoids |
| Lmnp003080 | | Hispidulin-8-C-(2''-O-xylosyl)xyloside | Flavonoids |
| mws1033 | | Homoeriodictyol | Flavonoids |
| Wahp004841 | | Homoeriodictyol-7-O-β-O-glucoside* | Flavonoids |
| Hmcp001598 | | Isorhamnetin-3-O-(2''-O-xylosyl)glucoside | Flavonoids |
| MWSHY0135 | | Isorhamnetin-3-O-rutinoside (Narcissin)* | Flavonoids |
| Lmlp002990 | | Isosaponarin(Isovitexin-4'-O-glucoside) | Flavonoids |
| mws1292 | | Isoschaftoside | Flavonoids |
| Lmyp004081 | | Isovitexin-2''-O-rhamnoside | Flavonoids |
| Smhp004476 | | Isovitexin-2''-O-xyloside | Flavonoids |
| Zbjn008081 | | Kaempferide (3,5,7-Trihydroxy-4'-methoxyflavone)* | Flavonoids |
| mws1068 | | Kaempferol (3,5,7,4'-Tetrahydroxyflavone) | Flavonoids |
| Zmcn003728 | | Kaempferol-3,7-O-diglucoside | Flavonoids |
| mws0919 | | Kaempferol-3-O-rhamnoside (Afzelin)(Kaempferin) | Flavonoids |
| HJAP155 | | Laricitrin-3-O-xyloside | Flavonoids |
| Wagp004984 | | Limocitrol 3-Glucoside | Flavonoids |
| Hmlp007121 | | Loureirin A* | Flavonoids |
| Zmlp003063 | | Luteolin-7-O-(2''-O-rhamnosyl)rutinoside | Flavonoids |
| Hmpp002612 | | Luteolin-7-O-gentiobioside | Flavonoids |
| Zmmp002345 | | Malvidin-3-O-rutinoside-5-O-glucoside | Flavonoids |
| MWSHY0072 | | Methylophiopogonanone B* | Flavonoids |
| pme0376 | | Naringenin (5,7,4'-Trihydroxyflavanone)* | Flavonoids |
| pme2960 | | Naringenin chalcone; 2',4,4',6'-Tetrahydroxychalcone | Flavonoids |
| Lmbn005454 | | Naringenin-5-methyl ether | Flavonoids |
| MWSN0152 | | Naringin Dihydrochalcone | Flavonoids |
| Ybnn007052 | | Norartocarpetin | Flavonoids |
| Cmxp003975 | | Okanin-4'-O-glucoside(Marein)* | Flavonoids |
| Zjmp102007 | | Ophiopogonanone E | Flavonoids |
| Lmtp003079 | | Peonidin-3-O-rutinoside | Flavonoids |
| Lacn005311 | | Phellatin | Flavonoids |
| Lacn004858 | | Phellavin | Flavonoids |
| pme1201 | | Phloretin | Flavonoids |
| mws2118 | | Phloretin-2'-O-glucoside (Phlorizin) | Flavonoids |
| Zjhp090608 | | Polygonatone D | Flavonoids |
| Wbtn007469 | | Protogenkwanone* | Flavonoids |
| Lcyp000677 | | Prunetin-4'-O-glucoside(Prunitrin)* | Flavonoids |
| Lmcp005645 | | Quercetin-3,4'-Dimethyl Ether | Flavonoids |
| pmp000596 | | Quercetin-3-O-(2''-O-galactosyl)glucoside | Flavonoids |
| Lmmp003091 | | Quercetin-3-O-(4''-O-glucosyl)rhamnoside | Flavonoids |
| Lmsp004166 | | Quercetin-3-O-glucoside-7-O-rhamnoside* | Flavonoids |
| Lmjp002461 | | Quercetin-3-O-neohesperidoside* | Flavonoids |
| pmn001583 | | Quercetin-3-O-robinobioside | Flavonoids |
| mws0059 | | Quercetin-3-O-rutinoside (Rutin) | Flavonoids |
| MWSHY0162 | | Quercetin-3-O-sophoroside (Baimaside) | Flavonoids |
| Smgp004575 | | Quercetin-5-O-β-D-glucoside | Flavonoids |
| Lacn005442 | | Rhamnosyl Phellamurin | Flavonoids |
| MWSN0167 | | Schaftoside | Flavonoids |
| Lmmp002463 | | Sexangularetin-3-O-glucoside-7-O-rhamnoside* | Flavonoids |
| Zbsn007580 | | Syringetin; 3,5,7,4'-Tetrahydroxy-3',5'-dimethoxyflavone | Flavonoids |
| HJN051 | | Tamarixetin-3-O-rutinoside | Flavonoids |
| Wbtn005728 | | Tetrahydroswertianolin | Flavonoids |
| pmb3041 | | Tricin-7-O-saccharic acid | Flavonoids |
| mws1172 | | Trifolirhizin (Maackiain-3-O-glucoside) | Flavonoids |
| Zbsn003878 | | Vicenin-3 | Flavonoids |
| Zmjp003291 | | Vitexin-2''-O-galactoside | Flavonoids |
| Zbsp003798 | | Vitexin-2''-O-glucoside | Flavonoids |
| pme3227 | | Vitexin-2''-O-rhamnoside | Flavonoids |
| Lalp009810 | | dracol | Flavonoids |
| Zjmp102042 | | iso-5,7-dihydroxy-6,8-dimethyl-3-(4'-hydroxy-3'-methoxybenzyl)chroman-4-one* | Flavonoids |
| Zjmp102003 | | iso-ophiopogonanone B* | Flavonoids |
| Zjmp102019 | | iso-ophiopogonone D | Flavonoids |
| Zjmp102012 | | ophiopogonanone H* | Flavonoids |
| Lacn005143 | | xylosyl Phellamurin | Flavonoids |
| Hmhn002787 | | (1'R,3R,5R,8'S)-Dihydrophaseic acid-O-β-D-glucoside | Phenolic acids |
| Hmbp006861 | | 1,2-O-Diferuloylglycerol | Phenolic acids |
| Lmsn004322 | | 1,6-Di-O-caffeoyl-β-D-glucose* | Phenolic acids |
| Zbjp005858 | | 1-(3,4-dihydroxy-S-methoxypheny)-7-(4-hydroxyphenyl)hept-1-ene-3,5-diol | Phenolic acids |
| Zbfn002690 | | 1-(4-Hydroxybenzoyl)Glucose; 25545-07-7 | Phenolic acids |
| Wasn002933 | | 1-O-(3,4,5-Trimethoxybenzoyl)-B-D-Glucopyranoside | Phenolic acids |
| Lmtn000940 | | 1-O-(3,4-Dihydroxy-5-methoxy-benzoyl)-glucoside | Phenolic acids |
| pmn001420 | | 1-O-Caffeoyl-β-D-glucose* | Phenolic acids |
| Lmsn004323 | | 1-O-Galloyl-2-O-Feruloyl-β-D-glucose* | Phenolic acids |
| Lmsn004092 | | 1-O-Galloyl-3-O-Feruloyl-β-D-glucose* | Phenolic acids |
| Lmsn003582 | | 1-O-Galloyl-4-O-p-Coumaroyl-β-D-glucose | Phenolic acids |
| Lmsn003409 | | 1-O-Galloyl-6-O-p-Coumaroyl-β-D-glucose | Phenolic acids |
| Lmfp001509 | | 1-O-Galloyl-rhamnose | Phenolic acids |
| pmb2871 | | 1-O-Gentisoyl-β-D-glucoside | Phenolic acids |
| Zmhn001926 | | 1-O-Salicyloyl-β-D-glucose | Phenolic acids |
| HJN003 | | 1-O-Sinapoyl-β-D-glucose | Phenolic acids |
| Lmtn002565 | | 1-O-Vanilloyl-D-Glucose | Phenolic acids |
| Lmsn003111 | | 1-O-p-Coumaroyl-β-D-glucose | Phenolic acids |
| pmb3068 | | 1-O-p-Coumaroylquinic acid | Phenolic acids |
| Jmwn002117 | | 2-(3,4-dihydroxyphenyl)ethanediol 1-O-β-D-glucopyranoside* | Phenolic acids |
| pme3083 | | 2-(Formylamino)benzoic acid | Phenolic acids |
| Lmhn001477 | | 2-Caffeoyl-L-tartaric acid (Caftaric acid) | Phenolic acids |
| Lmrn001951 | | 2-Hydroxy-3-(4-Hydroxyphenyl)Propanoic Acid* | Phenolic acids |
| Lmrn003000 | | 2-Hydroxy-3-phenylpropanoic acid | Phenolic acids |
| NK10252689 | | 2-Methoxy-5-nitrophenol | Phenolic acids |
| ML10179289 | | 2-Phenylethanol | Phenolic acids |
| Lmbp000728 | | 2-Phenyloxirane | Phenolic acids |
| Lakn003294 | | 2-β-D-Glucopyranosyloxy-5-hydroxyphenylacetic acidmethylester* | Phenolic acids |
| Wabn003563 | | 3'-O-Beta-D-Glucopyranosyl plumbagic acid | Phenolic acids |
| pmb2620 | | 3,4-Dimethoxycinnamic acid | Phenolic acids |
| pmn001525 | | 3,5-Digalloylshikimic acid | Phenolic acids |
| Lahp003063 | | 3,5-Dihydroxyphenyl1-O-(6-O-Galloyl-β-D-Glucopyranoside) | Phenolic acids |
| Lmjn003562 | | 3,6'-Diferuloylsucrose | Phenolic acids |
| Lmsn004970 | | 3,6-Di-O-caffeoyl glucose* | Phenolic acids |
| mws0467 | | 3-(4-Hydroxyphenyl)-propionic acid | Phenolic acids |
| MWSHC20102 | | 3-Hydroxy-1-(4-Hydroxy-3-Methoxyphenyl)Propan-1-One | Phenolic acids |
| Lafp002722 | | 3-Hydroxy-1-(4-hydroxy-3,5-dimethoxy-phenyl)-propan-1-one glucoside | Phenolic acids |
| pmn001690 | | 3-Hydroxy-4-isopropylbenzylalcohol-3-O-glucoside | Phenolic acids |
| pmn001712 | | 3-Hydroxy-4-isopropylbenzylalcohol-3-O-sophoroside | Phenolic acids |
| MWSslk066 | | 3-Hydroxy-4-methoxybenzoic acid; Isovanillic Acid | Phenolic acids |
| pmn001421 | | 3-O-p-Coumaroylquinic acid | Phenolic acids |
| Jmwn002494 | | 3-[4-(β-D-glucopyranoside)-phenylacrylic]-acid | Phenolic acids |
| HJN012 | | 4,7,9,9'-Tetrahydroxy-3,3'-dimethoxy-8-O-4'-neolignan | Phenolic acids |
| mws1336 | | 4-Aminobenzoic acid | Phenolic acids |
| pmb2497 | | 4-Hydroxy-3-methoxymandelate | Phenolic acids |
| mws0749 | | 4-Hydroxybenzoic acid | Phenolic acids |
| MWS5206 | | 4-Hydroxyphenyllactic Acid* | Phenolic acids |
| Lmbn004847 | | 4-Methoxyphenylpropionic acid | Phenolic acids |
| pme2828 | | 4-Nitrophenol | Phenolic acids |
| pmn001537 | | 4-O-Digalloyl-3,5-di-O-galloylquinic acid | Phenolic acids |
| Zmhn002227 | | 4-O-Glucosyl-sinapate | Phenolic acids |
| Lmzn001582 | | 5'-Glucosyloxyjasmanic acid | Phenolic acids |
| Hmtn001120 | | 5-(2-Hydroxyethyl)-2-O-glucosylphenol | Phenolic acids |
| Lmmn003663 | | 5-Glucosyloxy-2-Hydroxybenzoic acid methyl ester | Phenolic acids |
| pmb3074 | | 5-O-p-Coumaroylquinic acid | Phenolic acids |
| Hmbn002692 | | 6'-O-Feruloyl-D-sucrose | Phenolic acids |
| Lmsn003628 | | 6'-O-Sinapoylsucrose* | Phenolic acids |
| Ymjm000116 | | 6-O-Acetylarbutin | Phenolic acids |
| Zmhn001793 | | 6-O-Caffeoyl-D-glucose* | Phenolic acids |
| Lman003972 | | 6-O-Caffeoylarbutin* | Phenolic acids |
| Zmxn004029 | | 7,8-Dihydro-Buddlenol B(erythro) | Phenolic acids |
| Zmxn003874 | | 7,8-Dihydro-Buddlenol B(threo) | Phenolic acids |
| Cmsn000894 | | 7-O-Galloyl-D-sedoheptulose | Phenolic acids |
| Lasp003707 | | Acropyrone | Phenolic acids |
| Smln008909 | | Aloebarbendol | Phenolic acids |
| Zmdn003144 | | Aloesone-7-O-β-D-glucoside | Phenolic acids |
| Lmtn002233 | | Androsin | Phenolic acids |
| pmb2654 | | Anthranilate-1-O-Sophoroside | Phenolic acids |
| Labn003910 | | Benzyl β-primeveroside* | Phenolic acids |
| Cmjn004337 | | Benzyl-(2''-O-xylosyl)glucoside* | Phenolic acids |
| mws2120 | | Brevifolin carboxylic acid | Phenolic acids |
| MWSHC2023 | | Caffeic Acid 4-O-Glucoside | Phenolic acids |
| Labn003679 | | Caffeyl alcohol 4-O-β-D-glucopyranoside | Phenolic acids |
| mws1714 | | Chicoric Acid | Phenolic acids |
| mws0178 | | Chlorogenic acid (3-O-Caffeoylquinic acid)* | Phenolic acids |
| mws2213 | | Cinnamic acid | Phenolic acids |
| Lmhn003223 | | Cinnamoyltartaric acid | Phenolic acids |
| mws0906 | | Coniferin | Phenolic acids |
| mws0093 | | Coniferyl alcohol | Phenolic acids |
| mws2108 | | Cryptochlorogenic acid (4-O-Caffeoylquinic acid)* | Phenolic acids |
| Hmsn002272 | | Demethyl coniferin | Phenolic acids |
| Wbmp011762 | | Dibutylphthalate | Phenolic acids |
| Lmlp001436 | | Dihydrocaffeic acid | Phenolic acids |
| Lmmn000774 | | Dihydrocaffeoylglucose* | Phenolic acids |
| MWS20182 | | Dihydroferulic Acid | Phenolic acids |
| Wafn002827 | | Dihydroferulic acid glucoside | Phenolic acids |
| Lmmp010562 | | Diisooctyl Phthalate | Phenolic acids |
| mws0014 | | Ferulic acid* | Phenolic acids |
| Hmmn002544 | | Ferulic acid-4-O-glucoside | Phenolic acids |
| Lmhn003801 | | Feruloylsinapoyltartaric acid | Phenolic acids |
| pmn001671 | | Furanofructosyl-α-D-(6-mustard acyl)glucoside* | Phenolic acids |
| Labn004481 | | Gentisic acid-[6''-(3-hydroxy-3-methylglutaryl)]-glucoside | Phenolic acids |
| pmb3107 | | Glucosyringic Acid | Phenolic acids |
| MWSprf147 | | Glucovanillin | Phenolic acids |
| WaYn003084 | | Guaiacylglycerol 8-O-(1''-O-Glucosyl)Vanillic Acid Ether | Phenolic acids |
| Lmcn101912 | | Hellicoside | Phenolic acids |
| WaYn002313 | | Homosyringic Acid 4'-O-Glucoside | Phenolic acids |
| Wafn003070 | | Hydroxyferulic acid glucoside | Phenolic acids |
| pme0422 | | Isoferulic Acid* | Phenolic acids |
| WaYn002949 | | Isosalicin | Phenolic acids |
| Lmmn001294 | | Koaburaside* | Phenolic acids |
| Li512113 | | Maleoyl-caffeoylquinic acid | Phenolic acids |
| Hmtn001288 | | Methyl 2,4-dihydroxyphenylacetate* | Phenolic acids |
| Lmcn002982 | | Methyl 3-O-Methyl Gallate | Phenolic acids |
| Wcgn003981 | | Methyl 3-O-glucosyl-4-hydroxybenzoate | Phenolic acids |
| Zmgn004894 | | Methyl 4-hydroxybenzoate | Phenolic acids |
| Lmqp008175 | | Methyl Cinnamate | Phenolic acids |
| Lmdn003756 | | Methyl caffeate | Phenolic acids |
| Zblp002750 | | Methyl-3-(3-hydroxyphenyl)Propionate | Phenolic acids |
| pmb2819 | | O-Caffeoyl maltotriose | Phenolic acids |
| Wasn002902 | | O-p-Coumaroylgalactaric acid | Phenolic acids |
| MWS1848 | | Phenyl acetate | Phenolic acids |
| Hmhn003067 | | Phenylpropionic acid-O-β-D-glucopyranoside | Phenolic acids |
| Lmtn002109 | | Pleoside | Phenolic acids |
| Wasn002449 | | Protocatechuic acid 1-O-Rutinoside | Phenolic acids |
| Lmdn002671 | | Purpureaside C | Phenolic acids |
| Smhn003633 | | Pyrafortunoside A | Phenolic acids |
| Hmbn003347 | | Regaloside F | Phenolic acids |
| Cmbn003996 | | Regaloside G | Phenolic acids |
| Cmbn003192 | | Regaloside K | Phenolic acids |
| Labn002291 | | Rhamnosyl-gentisic acid-5-O-β-D-glucoside | Phenolic acids |
| MWSmce274 | | Salicyl Alcohol | Phenolic acids |
| pmb3142 | | Salicylic acid-2-O-glucoside | Phenolic acids |
| Ymjm000148 | | Salidroside | Phenolic acids |
| pmn001468 | | Sibiricose A3 | Phenolic acids |
| pmn001459 | | Sibiricose A6 | Phenolic acids |
| mws4085 | | Sinapic acid | Phenolic acids |
| pme3443 | | Sinapinaldehyde | Phenolic acids |
| mws0853 | | Sinapyl alcohol | Phenolic acids |
| mws0027 | | Syringic acid | Phenolic acids |
| mws0011 | | Syringin | Phenolic acids |
| pmb0751 | | Trans-5-O-(p-Coumaroyl)shikimate | Phenolic acids |
| mws0028 | | Vanillic acid | Phenolic acids |
| MWSmce632 | | Vanillin acetate | Phenolic acids |
| Zmtn001661 | | Vanilloloside | Phenolic acids |
| Hmgn001278 | | Verbasoside | Phenolic acids |
| Labn003405 | | Xylosyl tachioside | Phenolic acids |
| Labn002716 | | galloyl acetyl glucoside | Phenolic acids |
| Zmhn002301 | | p-Coumaric acid-4-O-glucoside | Phenolic acids |
| Ymjm000147 | | p-Hydroxyphenyl 6-O-(E)-caffeoyl-β-D-allopyranoside* | Phenolic acids |
| Lmbn002648 | | α-Hydroxycinnamic Acid* | Phenolic acids |
| Hmgn002038 | | β-Oxoacteoside | Phenolic acids |
| Waln010743 | | 1-(2,3-dihydroxypropoxy)-3-(((2-(dimethylamino)ethoxy)(hydroxy)phosphoryl)oxy)propan-2-yl (11Z,14Z)-octadeca-11,14-dienoate | Lipids |
| Waln009920 | | 1-(2,3-dihydroxypropoxy)-3-(((2-(dimethylamino)ethoxy)(hydroxy)phosphoryl)oxy)propan-2-yl (8E,11Z,14Z)-octadeca-8,11,14-trienoate | Lipids |
| Waln010449 | | 1-(2,3-dihydroxypropoxy)-3-(((2-(dimethylamino)ethoxy)(hydroxy)phosphoryl)oxy)propan-2-yl (E)-hexadec-9-enoate | Lipids |
| Waln011704 | | 1-(2,3-dihydroxypropoxy)-3-(((2-(dimethylamino)ethoxy)(hydroxy)phosphoryl)oxy)propan-2-yl (Z)-14-Octadecenoic Acid | Lipids |
| WaYn011606 | | 1-(2,3-dihydroxypropoxy)-3-(((2-(dimethylamino)ethoxy)(hydroxy)phosphoryl)oxy)propan-2-yl heptadecanoate | Lipids |
| Waln011524 | | 1-(2,3-dihydroxypropoxy)-3-(((2-(dimethylamino)ethoxy)(hydroxy)phosphoryl)oxy)propan-2-yl palmitate* | Lipids |
| Zbfn007626 | | 1-Linoleoyl-2-Lysophosphatidic Acid Monobutylamine Ester | Lipids |
| Lmhp112042 | | 1-Linoleoylglycerol* | Lipids |
| Wcdp010162 | | 1-Monolinolenoyl-Rac-Glycerol* | Lipids |
| Zbfn008434 | | 1-O-Linoleoyl-3-O-galactopyranosyl-L-glycerol | Lipids |
| Lmhp011562 | | 1-α-Linolenoyl-glycerol* | Lipids |
| Lmbn005487 | | 12,13-DHOME; (9Z)-12,13-Dihydroxyoctadec-9-enoic acid | Lipids |
| pmb2799 | | 12,13-Epoxy-9-Octadecenoic Acid | Lipids |
| Lmbn005369 | | 13(S)-HODE;13(S)-Hydroxyoctadeca-9Z,11E-dienoic acid* | Lipids |
| MWS80007 | | 13-Hydroperoxy-9Z,11E-octadecadienoic acid* | Lipids |
| MWS2430 | | 13-methylmyristic acid | Lipids |
| pmb2789 | | 13S-Hydroperoxy-6Z,9Z,11E-octadecatrienoic acid | Lipids |
| pmb2804 | | 13S-Hydroperoxy-9Z,11E-octadecadienoic acid | Lipids |
| Walp007738 | | 14-Amino-15-hydroxy-11-methylpentadecanoic acid | Lipids |
| Zmyn004676 | | 17-Hydroxylinolenic acid | Lipids |
| WaYn010475 | | 2-(2,3-dihydroxypropoxy)-3-(((2-(dimethylamino)ethoxy)(hydroxy)phosphoryl)oxy)propan-2-yl (E)-hexadec-9-enoate | Lipids |
| WaYn011395 | | 2-(2,3-dihydroxypropoxy)-3-(((2-(dimethylamino)ethoxy)(hydroxy)phosphoryl)oxy)propan-2-yl (Z)-14-Octadecenoic Acid | Lipids |
| Waln011009 | | 2-(2,3-dihydroxypropoxy)-3-(((2-(dimethylamino)ethoxy)(hydroxy)phosphoryl)oxy)propyl (11Z,14Z)-octadeca-11,14-dienoate | Lipids |
| Waln010192 | | 2-(2,3-dihydroxypropoxy)-3-(((2-(dimethylamino)ethoxy)(hydroxy)phosphoryl)oxy)propyl (8E,11Z,14Z)-octadeca-8,11,14-trienoate | Lipids |
| Waln011222 | | 2-(2,3-dihydroxypropoxy)-3-(((2-(dimethylamino)ethoxy)(hydroxy)phosphoryl)oxy)propyl palmitate* | Lipids |
| Walp004752 | | 2-Aminododecane-1,4-diol | Lipids |
| WaYp006462 | | 2-Aminohexadecane-1,5,15-triol | Lipids |
| Walp004906 | | 2-Aminotetradecane-1,11,13-triol | Lipids |
| Walp006011 | | 2-Aminotetradecane-1,4-diol | Lipids |
| WaYp005128 | | 2-Aminotetradecane-1,5,13-triol | Lipids |
| Zmjn003398 | | 2-Dodecenedioic acid | Lipids |
| Lmmn004951 | | 2-Hydroxy-4-methyl-3-undecanoyloxypentanoic acid methyl ester* | Lipids |
| Lmhp012042 | | 2-Linoleoylglycerol* | Lipids |
| Lmxp006440 | | 2-Palmitoylglycerol* | Lipids |
| Zmyn005384 | | 2R-Hydroxyoctadecanoic Acid* | Lipids |
| Zmyn004732 | | 2R-hydroxy-9Z,12Z,15Z-octadecatrienoic acid | Lipids |
| ZbYn010300 | | 3'-Palmitoyl sucrose | Lipids |
| ZbYn009262 | | 3'-linoleoyl sucrose* | Lipids |
| ML10195036 | | 3-Dehydrosphinganine | Lipids |
| Zmyn005252 | | 3-Hydroxy-palmitic acid methyl ester | Lipids |
| ZbYn009419 | | 3-Oleoyl sucrose* | Lipids |
| Lmbp005578 | | 4-Hydroxy-8-sphingenine | Lipids |
| pmb2221 | | 4-Hydroxysphinganine; Phytosphingosine | Lipids |
| Lmbn004685 | | 5S,8R-DiHODE; (5S,8R,9Z,12Z)-5,8-Dihydroxyoctadeca-9,12-dienoate | Lipids |
| Lmln007483 | | 6,9,10-Trihydroxyoctadec-7-enoic acid; Sanleng acid* | Lipids |
| Lmbn005287 | | 7S,8S-DiHODE; (9Z,12Z)-(7S,8S)-Dihydroxyoctadeca-9,12-dienoic acid* | Lipids |
| Wcsn010524 | | 8,11-Heptadecadienoic acid | Lipids |
| Lmbn005662 | | 9(10)-EpOME;(9R,10S)-(12Z)-9,10-Epoxyoctadecenoic acid | Lipids |
| Hmqn003054 | | 9,10,11-Trihydroxy-12-octadecenoic acid | Lipids |
| pmn001694 | | 9,10,13-Trihydroxy-11-Octadecenoic Acid | Lipids |
| Lmbn004240 | | 9,10-Dihydroxy-12,13-epoxyoctadecanoic acid | Lipids |
| Lmbn003970 | | 9,12,13-TriHOME; 9(S),12(S),13(S)-Trihydroxy-10(E)-octadecenoic acid | Lipids |
| pmn001691 | | 9,12,13-Trihydroxy-10,15-octadecadienoic acid | Lipids |
| pmb1574 | | 9,12-Octadecadien-6-Ynoic Acid | Lipids |
| Lmyn007883 | | 9,16-Dihydroxypalmitic acid | Lipids |
| pmb2791 | | 9-Hydroperoxy-10E,12,15Z-octadecatrienoic acid | Lipids |
| MWS80006 | | 9-Hydroperoxy-9Z,11E-Octadecadienoic Acid | Lipids |
| Zmyn004449 | | 9-Hydroxy-12-oxo-10(E),15(Z)-octadecadienoic acid | Lipids |
| pmn001689 | | 9-Hydroxy-12-oxo-15(Z)-octadecenoic acid* | Lipids |
| Rfmb087 | | 9-Hydroxy-13-oxo-10-octadecenoic Acid | Lipids |
| WaYn010298 | | 9-Hydroxyoctadeca-6,10,12,15-Tetraenoic Acid | Lipids |
| Wcdp007645 | | 9-Oxo-10,12-Octadecadienoic Acid | Lipids |
| pmb2787 | | 9-Oxo-10E,12Z-octadecadienoic acid* | Lipids |
| Hmqp005411 | | 9-Oxo-12Z-Octadecenoic acid | Lipids |
| Zmjn004133 | | 9S-Hydroperoxy-10E,12Z-octadecadienoic acid | Lipids |
| Rfmb091 | | 9S-Hydroxy-10E,12Z-octadecadienoic acid* | Lipids |
| mws0120 | | Choline Alfoscerate | Lipids |
| Lmbn005923 | | Crepenynic acid | Lipids |
| MWS4295 | | DL-2-hydroxystearic acid* | Lipids |
| pmb2640 | | Dodecanoic acid (Lauric acid) | Lipids |
| pmn001610 | | Eicosadienoic acid* | Lipids |
| mws0396 | | Elaidic Acid* | Lipids |
| Lmyn005812 | | Gingerglycolipid A | Lipids |
| Lmyn006011 | | Gingerglycolipid B | Lipids |
| Lmfp009701 | | Glycerol 9,11,13-octadecatrienoyl ester* | Lipids |
| Walp013004 | | Glycidyl Linoleate | Lipids |
| MWSslk132 | | Hexadecanedioic acid | Lipids |
| Zadn009075 | | Hydroperoxylinoleic acid* | Lipids |
| Lmbn007891 | | Hydroxy ricinoleic acid | Lipids |
| pmd0130 | | LysoPC 14:0 | Lipids |
| Lmhp009129 | | LysoPC 15:0(2n isomer)* | Lipids |
| pmb2319 | | LysoPC 15:0* | Lipids |
| pmb2260 | | LysoPC 15:1 | Lipids |
| pmb0855 | | LysoPC 16:0 | Lipids |
| pmd0132 | | LysoPC 16:0(2n isomer) | Lipids |
| Lmhp008833 | | LysoPC 16:1(2n isomer) | Lipids |
| pmb0863 | | LysoPC 16:2(2n isomer)* | Lipids |
| pma1303 | | LysoPC 16:2* | Lipids |
| Lmhp010515 | | LysoPC 17:0(2n isomer)* | Lipids |
| pmb2406 | | LysoPC 17:0* | Lipids |
| Lmhp009590 | | LysoPC 17:1 | Lipids |
| Lmhp008718 | | LysoPC 17:2 | Lipids |
| mws0126 | | LysoPC 18:0 | Lipids |
| pmd0136 | | LysoPC 18:0(2n isomer) | Lipids |
| pmp001281 | | LysoPC 18:1 | Lipids |
| Lmhp010190 | | LysoPC 18:1(2n isomer) | Lipids |
| pmp001273 | | LysoPC 18:2 | Lipids |
| pmp001251 | | LysoPC 18:2(2n isomer) | Lipids |
| pmb0854 | | LysoPC 18:3 | Lipids |
| pmb0865 | | LysoPC 18:3(2n isomer) | Lipids |
| Lmhp007840 | | LysoPC 19:2 | Lipids |
| Lmhp007598 | | LysoPC 19:2(2n isomer) | Lipids |
| Lmhp011549 | | LysoPC 20:1 | Lipids |
| pmd0147 | | LysoPC 20:2 | Lipids |
| pmd0146 | | LysoPC 20:2(2n isomer) | Lipids |
| Lmhp008337 | | LysoPE 14:0(2n isomer)* | Lipids |
| pmb0864 | | LysoPE 14:0* | Lipids |
| Lmhp008885 | | LysoPE 15:0(2n isomer)* | Lipids |
| Lmhp009187 | | LysoPE 15:0* | Lipids |
| Lmhp008440 | | LysoPE 15:1 | Lipids |
| pmb0876 | | LysoPE 16:0 | Lipids |
| pmd0160 | | LysoPE 16:0(2n isomer) | Lipids |
| Lmhp008763 | | LysoPE 16:1(2n isomer)* | Lipids |
| Lmhp009034 | | LysoPE 16:1* | Lipids |
| Lmhp010162 | | LysoPE 17:0 | Lipids |
| pmb0883 | | LysoPE 18:0 | Lipids |
| pmb0856 | | LysoPE 18:1(2n isomer)* | Lipids |
| mws0289 | | LysoPE 18:1* | Lipids |
| pmb0881 | | LysoPE 18:2 | Lipids |
| pmb0874 | | LysoPE 18:2(2n isomer) | Lipids |
| Lmhp008801 | | LysoPE 18:3 | Lipids |
| Lmhp008589 | | LysoPE 18:3(2n isomer) | Lipids |
| Lmhp010757 | | LysoPE 20:2 | Lipids |
| Lmhp010514 | | LysoPE 20:2(2n isomer) | Lipids |
| Yalp008047 | | Methyl 12-phenyldodecanoate | Lipids |
| MWSmce689 | | Methyl linolenate | Lipids |
| Lmxn015337 | | Methyl palmitate | Lipids |
| Lmsp010763 | | Monolinolenin* | Lipids |
| pmp001284 | | Monopalmitin* | Lipids |
| Wcdp009119 | | Octadeca-9,12,15-trienoic acid | Lipids |
| pme2827 | | Palmitaldehyde | Lipids |
| mws0359 | | Pentadecanoic Acid | Lipids |
| pmb0889 | | Punicic acid (9Z,11E,13Z-octadecatrienoic acid) | Lipids |
| Hmqn004825 | | Rabdosia acid A* | Lipids |
| MWS5231 | | Tridecanedioic acid | Lipids |
| MWS1900 | | Undecanedioic acid | Lipids |
| Wafn011571 | | alpha-Hydroxylinoleic acid* | Lipids |
| Lcsp012679 | | linolenoylethanolamine | Lipids |
| Lcsp013328 | | linoleoyl ethanolamine | Lipids |
| mws0367 | | α-Linolenic Acid* | Lipids |
| mws0366 | | γ-Linolenic Acid* | Lipids |
| Lmqn000432 | | 1-(sn-Glycero-3-phospho)-1D-myo-inositol | Others |
| Wmmp000181 | | 1´-O-Benzoylsucrose | Others |
| pmn001394 | | 2,3,5,4'-Tetrahydroxystilbene-2-O-glucoside | Others |
| Zmyn000268 | | 2,3-Dihydroxypropanal | Others |
| Hasp010605 | | 2,3-dihydroxypropyl (9Z,12Z,15Z)-octadeca-9,12,15-trienoate | Others |
| Lmbn001981 | | 2,5-Dihydroxybenzaldehyde | Others |
| Lmbn005172 | | 2,6-Dimethoxybenzaldehyde | Others |
| pmf0348 | | 2,6-Dimethyl-7-octene-2,3,6-triol | Others |
| pmp001267 | | 2-(Dodecylamino)-3-phenyl-1-propanol | Others |
| Wasn000528 | | 2-O-α-D-Glucopyranosyl-L-ascorbic acid | Others |
| Lssp210167 | | 2α,5α,14β-Triacetoxy-10β-O-(B-D glucopyranosy)taxa-4(20),11-diene | Others |
| Cmsp002787 | | 3,4'-Dihydroxy-3',5'-dimethoxypropiophenone | Others |
| Zbfn003643 | | 3,4,5-Trimethoxyphenol-β-D-apiofuranosyl-(1→6)-O-β-D-glucopyranoside | Others |
| Yaap003029 | | 3,4,5-trimethoxyphenyl acetate | Others |
| ZbWp001588 | | 3-(((S)-1-carboxy-2-phenylethyl)amino)-2,5-dihydroxycyclopentane-1-carboxylic acid | Others |
| Lhhp102901 | | 3-(α,4-Dihydroxy-3-methoxybenzyl)-4-(hydroxy-3-methoxybenzyl) | Others |
| Lmbn000198 | | 3-Dehydro-L-Threonic Acid | Others |
| Lchp000221 | | 3-Ethylidene-7-hydroxy-6-methoxyphthalide | Others |
| Lmyp003951 | | 3-Hydroxy-1-(4-hydroxy-3,5-dimethoxyphenyl)propan-1-one | Others |
| Wagp005297 | | 3-Hydroxy-beta-ionol 3-Glucoside | Others |
| Hmjp000461 | | 3-O-Glucoside-3-hydroxy-y-butyrolactone | Others |
| Zmgn000447 | | 3-Phospho-D-glyceric acid | Others |
| Lmjp101403 | | 3ξ-(1ξ-Hydroxyethyl)-7-hydroxy-1-isobenzofuranone | Others |
| MWSmce466 | | 4-Hydroxyacetophenone | Others |
| mws0628 | | 4-Hydroxybenzaldehyde | Others |
| Wasn000977 | | 4-O-galactopyranosylxylose | Others |
| pme2596 | | 4-Pyridoxic acid | Others |
| Zahn007990 | | 5-hydroxy-3,4-dimethyl-5-pentylfuran-2(5H)-one | Others |
| zjbp110819 | | 6,7-dimethoxy-2-[2-phenylethyl]chromone | Others |
| Latp003160 | | 6-Hydroxymellein | Others |
| Wasn000495 | | 6-O-alpha-L-arabinopyranosyl-D-glucopyranose | Others |
| pmf0319 | | Acetosyringone | Others |
| MWSslk235 | | Allitol | Others |
| MWSmce446 | | Cimifugin-7-O-glucoside | Others |
| Sazp005604 | | Comazaphilone E | Others |
| Lskp211395 | | Corchoionoside C | Others |
| Lsmp121707 | | Cuneataside E | Others |
| MWSmce676 | | D-Arabinose* | Others |
| mws1164 | | D-Fructose* | Others |
| Zmpn000199 | | D-Galactaric acid | Others |
| pmf0139 | | D-Galactose* | Others |
| Zmyn000110 | | D-Glucosamine 1-phosphate | Others |
| Zmyn000083 | | D-Glucose 1,6-bisphosphate | Others |
| mws0866 | | D-Glucose 6-phosphate* | Others |
| mws4170 | | D-Glucose* | Others |
| MA10039641 | | D-Lactose* | Others |
| Lmsn000381 | | D-Maltose* | Others |
| mws1155 | | D-Mannitol* | Others |
| pmf0138 | | D-Mannose* | Others |
| mws1337 | | D-Pantothenic Acid | Others |
| mws2104 | | D-Pinitol | Others |
| pme3163 | | D-Sedoheptuiose 7-phosphate | Others |
| mws0214 | | D-Sorbitol | Others |
| pme0519 | | D-Sucrose* | Others |
| mws0889 | | D-Threonic Acid | Others |
| pma0134 | | D-Threose | Others |
| mws0264 | | D-Trehalose* | Others |
| mws0344 | | D-Xylonic acid | Others |
| MWSmce682 | | DL-Xylose* | Others |
| pmb2653 | | DMelezitose O-rhamnoside | Others |
| Lmsn000954 | | Dambonitol | Others |
| Lmxn000380 | | Digalactosylglycerol | Others |
| Zmzn000078 | | Dihydroxyacetone phosphate | Others |
| Wafn003485 | | Dihydroxyoctanoic acid glucoside | Others |
| pme2237 | | Dulcitol* | Others |
| Zmbp000449 | | Epigoitrin | Others |
| Lfmp123024 | | Erigeside C | Others |
| pmn001380 | | Eucommiol | Others |
| Lhhp120823 | | Eugenyl formate | Others |
| mws1080 | | Galactinol | Others |
| Wasn006585 | | Geranyl 3-O-xylopyranosyl-glucopyranoside | Others |
| Lmmp000647 | | Glucan | Others |
| pmb3081 | | Glucaric acid-1-Phosphate | Others |
| pme0534 | | Gluconic acid | Others |
| Wafn003182 | | Glucopyranose 6-Hydroxydecanoate | Others |
| Wasn002394 | | Glucosyl 2,6-dimethyl-8-oxoocta-2,6-dienoic acid | Others |
| Wasn002508 | | Glucosyl 7-methyl-3-methyleneoctane-1,2,6,7-tetraol | Others |
| Lhhp120816 | | Hydroxyeugenol | Others |
| Hmln003754 | | Icariside E5 | Others |
| Wasn001007 | | Isoascorbic acid 2-O-glucoside | Others |
| mws5038 | | Isomaltulose* | Others |
| MWSmce040 | | Isovanillin | Others |
| Hmfn000531 | | L-Ascorbic acid (Vitamin C) | Others |
| MWSmce165 | | L-Fucitol | Others |
| MWSmce576 | | L-Glucose* | Others |
| Lhmp110119 | | Magnostellin D | Others |
| MWS1983 | | Maltitol | Others |
| MWS0442 | | Maltotriose | Others |
| MWSmce486 | | Manninotriose | Others |
| mws1333 | | Melibiose | Others |
| pme2755 | | N-Acetyl-D-glucosamine | Others |
| mws4174 | | N-Acetyl-D-mannosamine | Others |
| mws0133 | | Nicotinamide | Others |
| pma3101 | | Nicotinate D-ribonucleoside | Others |
| pme0490 | | Nicotinic acid (Vitamin B3) | Others |
| Wcdp010708 | | Octadeca-2,9,12,15-tetraen-1-ol | Others |
| Zbbp004112 | | Platanionoside C | Others |
| Zbbp005287 | | Platanionoside E | Others |
| Jmyn006217 | | Polygonatone B | Others |
| MWSHC2067 | | Protocatechualdehyde | Others |
| Lcyn000686 | | Prunustomentosanan A | Others |
| Lhmp110110 | | Pyramidatin F | Others |
| Zmjp000624 | | Pyridoxal | Others |
| pme1383 | | Pyridoxine | Others |
| pmb0789 | | Pyridoxine-5'-O-glucoside | Others |
| mws0232 | | Riboflavin (Vitamin B2) | Others |
| pmn001423 | | Roseoside | Others |
| Wmcp000010 | | Sammangaoside A | Others |
| Zmsn003194 | | Secologaoside | Others |
| mws1089 | | Sucrose-6-phosphate | Others |
| mws1350 | | Syringaldehyde; 4-Hydroxy-3,5-Dimethoxybenzaldehyde | Others |
| mws2523 | | Trehalose 6-phosphate | Others |
| mws0458 | | Vanillin; 4-Hydroxy-3-Methoxybenzaldehyde | Others |
| WaYn002408 | | [(1R,2R)-cyclohexane-1,2-diol]Beta-D-Galactopyranoside | Others |
| Wcdp000970 | | acetophenone | Others |
| HX1340 | | epiloliolide | Others |
| Yxxn002325 | | icariside D1 | Others |
| Wmmn000125 | | mudanpioside E | Others |
| Zamn00402 | | sorbose* | Others |
| Lmfp000418 | | α-D-glucosyl-(1→1')-3'-amino-3'-deoxy-β-D-glucoside | Others |
| Zjbp081705 | | 1,2-dihydroxy-Diosgenin-Glc | Steroids |
| Zjhp082612 | | 14-Dehydroxy-neoprazerigenin A-Glc | Steroids |
| Zjhp082623 | | 14-Dehydroxy-neoprazerigenin A-Glc-Glc | Steroids |
| Zjhp083010 | | 14-Dehydroxy-neoprazerigenin A-Glc-Glc-Glc-Ara-Ara | Steroids |
| Zjhp082684 | | 14-Dehydroxy-neoprazerigenin A-Glc-Glc-Glc-Glc | Steroids |
| Zjhp082665 | | 14-Dehydroxy-neoprazerigenin A-Glc-Glc-Glc-Xyl | Steroids |
| Zjcp102016 | | 17-Hydroxyspirost-5-en-3-yl-2-O-[6-deoxymannosyl(1→2)]glucoside (Polyphyllin VI) | Steroids |
| Zjzp081807 | | 2-Hydroxy diosgenin-Glc | Steroids |
| Zjzp081819 | | 2-Hydroxy diosgenin-Glc-Glc | Steroids |
| Zjzp081866 | | 2-Hydroxy diosgenin-Glc-Glc-Glc-Glc | Steroids |
| Zjmp101931 | | 25(R)-ruscogenin-Glc-Glc | Steroids |
| Zjcp102112 | | 26-O-Glucosyl-3,20,26-trihydroxyfurostan-5,22-diene-3-O-rhamnosyl(1→4)rhamnosyl(1→4)rhamnosyl(1→4)glucoside (Padelaoside C)* | Steroids |
| Zjxp101245 | | 26-O-Glucosyl-furost-25(27)-ene-3,22,26-triol-3-O-glucosyl(1→2)[glucosyl(1→3)]-glucosyl(1→4)-galactoside | Steroids |
| Ladp003715 | | 26-O-β-D-glucopyranose Glucosyl-(25R)-△5(6)-ene-16,22-diketone-furostane-3β,26-diol-3-O-α-L-rhamnosyl-(1→4)-O-α-L-rhamnosyl-(1→4)-[O-α-L-rhamnosyl-(1→2)]-O-β-D-pyranGlucoside* | Steroids |
| Jmyn004251 | | 3,17,22,26-Tetrahydroxy-furost-5-ene-3-O-glucosyl(1→2)glucosyl(1→4)galactoside-26-O-glucoside (Pratioside B) | Steroids |
| Zjcp102111 | | 3,20,26-Trihydroxy-furostan-5,22-diene-3-O-rhamnosyl-(1→2)-[rhamnosyl(1→4)rhamnosyl(1→4)]glucoside-26-O-glucoside (Smilaxchinoside B) | Steroids |
| Zjhp082650 | | 3,24-Dihydroxy-spirostan-5-en-12-one-3-O-glucosyl(1→2)glucosyl(1→4)galactoside (Kingianoside I) | Steroids |
| Hmdp003064 | | 3,24-Dihydroxy-spirostan-5-en-12-one-3-O-glucosyl(1→4)galactoside (Kingianoside H) | Steroids |
| Qabp004993 | | 3-Epineoruscogenin-glucose-glucose-glucose-glucose-glucoside | Steroids |
| Zjhp083018 | | 3-Hydroxy-cholest-5,14,16-trien-22-one-3-O-glucosyl(1→2)[xylosyl(1→3)]glucosyl(1→4)galactoside-26-O-glucoside (Polygonatumoside A) | Steroids |
| Zjhp082607 | | 3-Hydroxyspirost-5-en-12-one (Gentrogenin)* | Steroids |
| Zjhp082606 | | 3-Hydroxyspirost-5-en-12-one* | Steroids |
| Layp005375 | | 3-O-{O-β-D-glucopyranosyl-(1→2)-O-[β-D-glucopyra-nosyl-(1→3)]-O-β-D-glucopyranosyl-(1→4)-β-D-galacto-pyranoside} | Steroids |
| Zjbp081703 | | 3-hydroxy-5,20-diene-26 furostanol-Glc | Steroids |
| Zjbp081707 | | 6-one-sarsasapogenin-Glc | Steroids |
| Qadp004838 | | Aspidistrin | Steroids |
| Qabp004961 | | Dehydroxy Epiruscogenin-glucose-glucose-glucose-glucose-glucoside* | Steroids |
| Qabp005310 | | Dehydroxy Epiruscogenin-glucose-glucose-glucoside | Steroids |
| Qabp008552 | | Dehydroxy Epiruscogenin-glucose-glucoside | Steroids |
| Zjhp082602 | | Diosgenin | Steroids |
| Lhlp102620 | | Diosgenin pentaglucoside* | Steroids |
| MWSmce399 | | Diosgenin-3-O-glucoside (Trillin) | Steroids |
| Zjhp082616 | | Diosgenin-Gal* | Steroids |
| Zjhp082628 | | Diosgenin-Gal-Glc | Steroids |
| Zjhp082613 | | Diosgenin-Glc | Steroids |
| Zjhp082624 | | Diosgenin-Glc-Glc* | Steroids |
| Zjzp081862 | | Diosgenin-Glc-Glc-Glc-Glc | Steroids |
| Zjzp081882 | | Diosgenin-Glc-Glc-Glc-Glc-Glc | Steroids |
| Zjzp081871 | | Diosgenin-Glc-Glc-Glc-Glc-Xyl* | Steroids |
| Jmyp005224 | | Furost-5,20(22)-diene-3,26-diol-26-O-glucoside | Steroids |
| Jmyn004445 | | Furost-5-ene-3,22,26-triol-3-O-glucosyl(1→2)[xylosyl(1→3)]glucosyl(1→4)galactoside-26-O-glucoside (Timosaponin H1) | Steroids |
| Cmdp004421 | | Gentrogenin-3-O-glucosyl(1→4)fucoside (Kingianoside B) | Steroids |
| Zjhp082617 | | Gentrogenin-Glc | Steroids |
| Layp002986 | | Glucosyl-3-O-(O-β-D-glucopyranosyl-(1→2)-O-[β-D-xylopyranosyl-(1→3)]-O-β-D-glucopyranosyl-(1→4)-β-D-galactopyranoside) | Steroids |
| Layp003502 | | Glucosyl-hecogenin 3-O-{O-β-D-glucopyranosyl-(1→2)-O-[β-D-xylopyranosyl-(1→3)]-O-β-D-glucopyranosyl-(1→4)-β-D-galactopyranoside}* | Steroids |
| Lhlp102623 | | Hydroxydiosgenin glucose xylose glucoside* | Steroids |
| Zjhp082618 | | Isochiapagenin-Glc | Steroids |
| Zjhp083026 | | Isochiapagenin-Glc-Glc-Glc-Glc-Glc | Steroids |
| Qayp003699 | | Manogenin-glucose-glucose-arabinose-glucose-glucoside | Steroids |
| Zjcp102008 | | Parisvietnaside C/chonglouoside SL-19 | Steroids |
| Zmcp006626 | | Pennogenin-3-O-glucoside | Steroids |
| pmp001035 | | Sarsasapogenin-3-O-galactoside (Timosaponin A-I) | Steroids |
| Zjzp081883 | | Sarsasapogenin-Glc-Glc-Glc-Glc-Glc | Steroids |
| Zjzp081873 | | Sarsasapogenin-Glc-Glc-Glc-Glc-Xyl | Steroids |
| Zjxp101210 | | Spirost-3,12-diol-3-O-glucosyl(1→2)galactoside | Steroids |
| Lsjp211306 | | Spirost-4-en-3-one (Diosgenone) | Steroids |
| Jmyp005686 | | Spirost-5(6)en-3-ol-3-O-glucosyl(1→2)[xylosyl(1→3)]-glucosyl(1→4)galactoside | Steroids |
| Zjhp082677 | | Spirost-5-en-12-one-3-O-glucosyl(1→2)[xylosyl(1→3)]glucosyl(1→4)galactoside | Steroids |
| Hmdp002882 | | Spirost-5-en-12-one-3-O-glucosyl(1→2)glucosyl(1→4)galactoside (Pratioside D1) | Steroids |
| Zmcp010827 | | Spirost-5-en-3-ol (Diosgenin) | Steroids |
| Zjhp082688 | | Spirost-5-en-3-ol-3-O-glucosyl(1→2)[glucosyl(1→3)]glucosyl(1→4)galactoside* | Steroids |
| Hmdp003423 | | Spirost-5-en-3-ol-3-O-glucosyl(1→2)glucosyl(l→4)galactoside (Neosibiricoside D) | Steroids |
| Jmyp004233 | | Spirost-5-en-3-ol-3-O-glucosyl(1→4)galactoside* | Steroids |
| Jmyp003897 | | Spirost-5-ene-3,14-diol-3-O-glucosyl(1→2)[glucosyl(1→3)]glucosyl(1→4)galactoside | Steroids |
| Jmyn006303 | | Spirost-5-ene-3,14-diol-3-O-glucosyl(1→2)[xylosyl(1→3)]glucosyl(1→4)galactoside | Steroids |
| Zjhp082648 | | Spirost-5-ene-3,14-diol-3-O-glucosyl(1→2)glucosyl(1→4)galactoside (Polygoside A; Polygoside B) | Steroids |
| Jmyp005196 | | Spirosta-5,14-dien-3-ol-3-O-{glucosyl(1→2)[xylosyl(1→3)]glucosyl(1→4)}-galactoside | Steroids |
| Zjzp081802 | | Spirostan-3-ol (Isosarsapogenin) | Steroids |
| Zjxp101204 | | Spirostane-25(27)-en-3,12-diol-3-O-glucosyl(1→2)galactoside (Macrostemonoside S) | Steroids |
| Lazp004368 | | Triglucosyl 3-epiruscogenin-3-O-β-D-glucopyranoside | Steroids |
| Hmdp003401 | | Trillin-6'-O-glucoside | Steroids |
| Hmdp003288 | | Trillin-6'-O-sophorotrioside* | Steroids |
| Lazp004399 | | Tupichinin B | Steroids |
| Qadp004840 | | Typaspidoside L* | Steroids |
| Zjhp082626 | | Yamogenin-Glc-Glc* | Steroids |
| Layp003047 | | Yayoisaponin B | Steroids |
| Zjhp082686 | | iso-14-Dehydroxy-neoprazerigenin A-Glc-Glc-Glc-Glc | Steroids |
| Zjhp082668 | | iso-14-Dehydroxy-neoprazerigenin A-Glc-Xyl-Glc-Glc | Steroids |
| Zjzp081808 | | iso-2-Hydroxy diosgenin-Glc | Steroids |
| Zjhp082615 | | iso-Diosgenin-Gal | Steroids |
| Zjhp082614 | | iso-Diosgenin-Glc | Steroids |
| Zjhp082625 | | iso-Diosgenin-Glc-Glc* | Steroids |
| Zjhp083005 | | iso-Pennogenin-Glc-Glc-Glc-acetyl Glc | Steroids |
| Zjhp082627 | | iso-Yamogenin-Glc-Glc | Steroids |
| Zjmp101909 | | isodiosgenin-Glc-Glc | Steroids |
| Layp006398 | | pennogenin 3-O-α-L-rhamnopyranosyl-(1→4)-α-L-rhamnopyranosyl-(1→4)[O-α-L-rhamnopyranosyl-(1→2)]-β-D-glucopyranoside | Steroids |
| mws4052 | | 1-Aminocyclopropane-1-carboxylic acid | Organic acids |
| Lmbn000612 | | 1-Pyrroline-4-hydroxy-2-carboxylic acid | Organic acids |
| Lcsn006884 | | 2,2'-(3-methylcyclohexane-1,1-diyl)diacetic acid | Organic acids |
| Wmzn000227 | | 2,2-Dimethylsuccinic acid | Organic acids |
| pme0278 | | 2,6-Diaminooimelic acid | Organic acids |
| Lmbn001609 | | 2-Acetyl-2-Hydroxybutanoic Acid | Organic acids |
| Lmbn001288 | | 2-Hydroxy-2-methyl-3-oxobutanoic acid* | Organic acids |
| Lmrn002746 | | 2-Hydroxy-4-methylpentanoic acid | Organic acids |
| Lmmn003323 | | 2-Hydroxyhexadecanoic acid | Organic acids |
| mws0341 | | 2-Hydroxyisocaproic acid | Organic acids |
| Lcsn006335 | | 2-Hydroxymyristic acid | Organic acids |
| pmb3101 | | 2-Isopropylmalic Acid | Organic acids |
| Lmhn004446 | | 2-Methylene-3-(6-hydroxyhexyl)-butanedioic acid | Organic acids |
| mws0473 | | 2-Methylsuccinic acid* | Organic acids |
| pme1216 | | 2-Picolinic acid | Organic acids |
| Zmgn001448 | | 2-Propylmalic Acid* | Organic acids |
| Lmbn002072 | | 2-Propylsuccinic acid* | Organic acids |
| Zbqp000579 | | 2-amino-3-(1H-pyrazol-1-yl)propanoic acid | Organic acids |
| Wasn003197 | | 3-(Beta-D-Glucopyranosyloxy)-5-Hydroxyhexanoic Acid Methyl Ester | Organic acids |
| mws1039 | | 3-Aminoisobutyric acid | Organic acids |
| Zmpn000638 | | 3-Guanidinopropionic acid | Organic acids |
| mws0576 | | 3-Hydroxybutyric acid | Organic acids |
| Lmbn001754 | | 3-Isopropylmalic Acid* | Organic acids |
| Lmbn000216 | | 3-Methylmalic acid | Organic acids |
| ZbBn002068 | | 3-methyl-ShikimicAcid | Organic acids |
| Zmtn001464 | | 4,8-Dihydroxyquinoline-2-carboxylic acid | Organic acids |
| pme0295 | | 4-Acetamidobutyric acid | Organic acids |
| mws0567 | | 4-Guanidinobutyric acid | Organic acids |
| Lmbn001364 | | 4-Hydroxy-2-Oxopentanoic Acid* | Organic acids |
| Lmbn001467 | | 5-Acetamidopentanoic Acid | Organic acids |
| pme0120 | | 5-Aminovaleric acid | Organic acids |
| pme0274 | | 6-Aminocaproic acid | Organic acids |
| mws0972 | | 6-Hydroxyhexanoic acid | Organic acids |
| Lmbn003524 | | 9-Oxononanoic acid | Organic acids |
| pme2923 | | Acetoxyacetic acid | Organic acids |
| MWS0155 | | Adenylocuccinic Acid | Organic acids |
| pme3096 | | Aminomalonic acid | Organic acids |
| mws0237 | | Azelaic acid | Organic acids |
| MWSmce210 | | Chelidonic acid | Organic acids |
| pme2550 | | Cis-Aconitic acid | Organic acids |
| mws0425 | | Citraconic acid | Organic acids |
| mws0281 | | Citric Acid | Organic acids |
| WaYn000716 | | Citric Acid diglucoside | Organic acids |
| Wayn000884 | | Citric acid glucoside | Organic acids |
| MWS1709 | | D-Malic acid* | Organic acids |
| MWSmce183 | | D-Mandelic acid | Organic acids |
| MWS0274 | | DL-3-Phenyllactic acid | Organic acids |
| pme3186 | | DL-Glyceraldehyde-3-phosphate | Organic acids |
| mws0267 | | DL-Glyceric Acid | Organic acids |
| Lmmn000806 | | Dimethylmalonic acid* | Organic acids |
| mws0376 | | Fumaric acid | Organic acids |
| Lmjp002931 | | Fusaric acid | Organic acids |
| Wasn001627 | | Glucosyl 2,3-Dihydroxy-2-Methylbutanoic Acid | Organic acids |
| Wasn003258 | | Glucosyl 2-Hydroxy-4-Methylpentanoic Acid | Organic acids |
| pme0243 | | Glutaric acid* | Organic acids |
| Wayn001528 | | Homocitrate | Organic acids |
| MWS1882 | | Iminodiacetic acid | Organic acids |
| Zmyn000453 | | Isocitric Acid | Organic acids |
| Zmgn000217 | | Itaconic acid | Organic acids |
| Lcsn000415 | | Keto-Deoxy-Nonulonic acid | Organic acids |
| MWS0811 | | L-Pipecolic Acid | Organic acids |
| mws0262 | | L-Tartaric acid | Organic acids |
| pme2362 | | Mandelic acid | Organic acids |
| Lmyn002403 | | Mandelic acid-β-glucoside | Organic acids |
| mws0470 | | Methylmalonic acid* | Organic acids |
| pme3154 | | Mevalonic acid | Organic acids |
| Lmmn002164 | | Monomethyl succinate* | Organic acids |
| Lmyn000160 | | Mucic acid Dimethyl Ester | Organic acids |
| pme3207 | | Muconic acid | Organic acids |
| pmf0096 | | Oxalic acid | Organic acids |
| Zmjn001813 | | Pimelic acid* | Organic acids |
| mws0601 | | Pyrrole-2-carboxylic acid | Organic acids |
| mws0277 | | Quinic Acid | Organic acids |
| pme0266 | | Sebacic acid | Organic acids |
| ZbBn000907 | | Shikimic Acid-3-Phosphate | Organic acids |
| mws0242 | | Suberic Acid | Organic acids |
| mws0192 | | Succinic acid* | Organic acids |
| Lmgn007652 | | Tianshic acid | Organic acids |
| MWS4414 | | trans-2-Butene-1,4-dicarboxylic Acid | Organic acids |
| pme2380 | | α-Ketoglutaric acid | Organic acids |
| mws0147 | | β-Hydroxyisovaleric acid | Organic acids |
| HX1356 | | (7R,8S)-9-acetyl-dehydrodiconiferyl alcohol | Lignans and Coumarins |
| HX1371 | | (7S,8R)-dihydrodehydrodiconferyl alcohol | Lignans and Coumarins |
| pmn001375 | | 1-Hydroxypinoresinol-1-O-Glucoside | Lignans and Coumarins |
| MWSmce439 | | 3,4-Dihydrocoumarin | Lignans and Coumarins |
| pme2996 | | 4-Hydroxycoumarin | Lignans and Coumarins |
| Hmgn002833 | | 4-Ketopinoresinol | Lignans and Coumarins |
| Yalp002838 | | 4-Methoxy-5-(hydroxymethyl)-2H-1-benzopyran-2-one | Lignans and Coumarins |
| Lmmn002260 | | 5'-Methoxyisolariciresinol-9'-O-glucoside | Lignans and Coumarins |
| Hmln002355 | | 5'-Methoxymatairesinoside* | Lignans and Coumarins |
| Cmpp003619 | | 6,7-Dihydroxy-4-methylcoumarin | Lignans and Coumarins |
| MWSslk183 | | 7,8-Dihydroxy-4-phenylcoumarin | Lignans and Coumarins |
| MWSslk194 | | 7-Hydroxy-4-methyl-8-nitrocoumarin | Lignans and Coumarins |
| HX1341 | | 7-hydroxy-2H-1-benzopyran-2-one | Lignans and Coumarins |
| Xmbn007315 | | Chestnutlignansoide | Lignans and Coumarins |
| Cmsp003083 | | Dehydrodiconiferyl alcohol-4-O-glucoside* | Lignans and Coumarins |
| Lmsp003655 | | Dehydrodiconiferyl alcohol-gamma'-O-glucoside* | Lignans and Coumarins |
| Lcyn000106 | | Dichotomoside A | Lignans and Coumarins |
| Zmxp005324 | | Dihydrodehydrodiconiferyl alcohol | Lignans and Coumarins |
| Lcgn000236 | | Dihydrodehydrodiconiferyl alcohol-4,9-di-O-β-D-glucopyranoside | Lignans and Coumarins |
| Zmcp001750 | | Eleutheroside B1* | Lignans and Coumarins |
| mws1603 | | Eleutheroside E | Lignans and Coumarins |
| MWSHC20189 | | Epipinoresinol* | Lignans and Coumarins |
| Lazn006735 | | Erythro-Guaiacylglycerol-β-O-4'-dehydrodisinapyl Ether | Lignans and Coumarins |
| Lazn003953 | | Erythro-Guaiacylglycerol-β-dihydroconiferyl Ether glucoside | Lignans and Coumarins |
| pmn001371 | | Eucommin A* | Lignans and Coumarins |
| WaYn006966 | | Ficusesquilignan B | Lignans and Coumarins |
| Lmbp003208 | | Fraxidin-8-O-glucoside* | Lignans and Coumarins |
| mws1639 | | Isofraxidin | Lignans and Coumarins |
| Walp003242 | | Isofraxidin 7-O-glucosylglucoside | Lignans and Coumarins |
| Lmjp003090 | | Isofraxidin-7-O-glucoside | Lignans and Coumarins |
| Lhbp111734 | | Isolariciresinol | Lignans and Coumarins |
| Lmmn002274 | | Isolariciresinol-9'-O-glucoside | Lignans and Coumarins |
| Cmsp005051 | | Isolariciresinol-9-O-glucoside | Lignans and Coumarins |
| Zmdn003762 | | Isosyringinoside | Lignans and Coumarins |
| Lmnn004130 | | Lappaol C | Lignans and Coumarins |
| Hmcn002743 | | Lirioresinol A | Lignans and Coumarins |
| Cmmn005231 | | Massoniresinol; Vladinol A | Lignans and Coumarins |
| Hmln001931 | | Matairesinol-4,4'-O-diglucoside | Lignans and Coumarins |
| pmn001372 | | Medioresinol-4,4'-di-O-glucoside | Lignans and Coumarins |
| Lazn003560 | | Methoxy-erythro-Guaiacyl glycerol β-threo-syringyl glycerol ether | Lignans and Coumarins |
| Lmmn004685 | | Nectandrin B | Lignans and Coumarins |
| Hmln101664 | | Nortrachelogenin-4'-O-gentiobioside | Lignans and Coumarins |
| Hmln002100 | | Nortrachelogenin-4-O-glucoside | Lignans and Coumarins |
| pmn001368 | | Olivil-4,4'-Di-O-glucoside | Lignans and Coumarins |
| Lmwp102713 | | Peucedanol | Lignans and Coumarins |
| mws0097 | | Pinoresinol* | Lignans and Coumarins |
| pmn001378 | | Pinoresinol-4-O-glucoside | Lignans and Coumarins |
| mws0096 | | Piperitol | Lignans and Coumarins |
| MWSHC20155 | | Scopoletin (7-Hydroxy-6-methoxycoumarin) | Lignans and Coumarins |
| Lskp211262 | | Secoisolariciresinol | Lignans and Coumarins |
| HJN054 | | Secoisolariciresinol-9'-O-xyloside | Lignans and Coumarins |
| Zmln002252 | | Sideretin (5,7,8-Trihydroxy-6-methoxycoumarin) | Lignans and Coumarins |
| Lhmp121005 | | Symplocosin* | Lignans and Coumarins |
| MWS20152 | | Syringaresinol | Lignans and Coumarins |
| Rfmb26201 | | Syringaresinol-4'-O-(6''-acetyl)glucoside | Lignans and Coumarins |
| Yamn004825 | | Syringaresinol-4'-O-glucoside | Lignans and Coumarins |
| MWSHC2047 | | Syringaresinol-4'-O-glucoside; Acanthoside B | Lignans and Coumarins |
| Cmqn003939 | | Syringaresinol-4,4'-O-bis-β-D-glucoside; Liriodendrin | Lignans and Coumarins |
| Hmln003169 | | Trachelogenin | Lignans and Coumarins |
| Hmln002161 | | Trachelogenin-4'-O-gentiobioside | Lignans and Coumarins |
| Lcgn000239 | | dihydrodehydrodiconiferyl alcohol-9-O-β-D-glucopyranoside | Lignans and Coumarins |
| HX1372 | | guaiacylglycerol-β-coniferyl ether | Lignans and Coumarins |
| Zmcp102703 | | saikolignanoside D* | Lignans and Coumarins |
| Hahn002315 | | syringaresinol-4-O-β-D-glucopyranoside | Lignans and Coumarins |
| mws0847 | | 1-Methyladenine | Nucleotides and derivatives |
| pme3961 | | 2'-Deoxyadenosine* | Nucleotides and derivatives |
| pme3184 | | 2'-Deoxyadenosine-5'-monophosphate | Nucleotides and derivatives |
| pme1194 | | 2'-Deoxycytidine | Nucleotides and derivatives |
| pme1184 | | 2'-Deoxyguanosine | Nucleotides and derivatives |
| pme2776 | | 2'-Deoxyinosine | Nucleotides and derivatives |
| pme3882 | | 2'-Deoxyuridine | Nucleotides and derivatives |
| Hmhp001812 | | 2'-O-Methyladenosine | Nucleotides and derivatives |
| pme3967 | | 2-(Dimethylamino)guanosine* | Nucleotides and derivatives |
| pmb2507 | | 2-Deoxyribose-1-phosphate* | Nucleotides and derivatives |
| mws0863 | | 2-Deoxyribose-5'-phosphate* | Nucleotides and derivatives |
| pme1266 | | 3-Methylxanthine | Nucleotides and derivatives |
| Zmmp002106 | | 4-methyl-1,5,2,3-dioxadiazinan-2-amine | Nucleotides and derivatives |
| pme1474 | | 5'-Deoxy-5'-(methylthio)adenosine | Nucleotides and derivatives |
| MWSmce116 | | 5'-Deoxyadenosine* | Nucleotides and derivatives |
| Lmqp000329 | | 5-Aminoimidazole ribonucleotide | Nucleotides and derivatives |
| MWS2910 | | 5-Methyl-2'-Deoxycytidine | Nucleotides and derivatives |
| mws0572 | | 5-Methylcytosine | Nucleotides and derivatives |
| pme1187 | | 5-Methyluridine | Nucleotides and derivatives |
| MWS4525 | | 6-O-methylguanine | Nucleotides and derivatives |
| MWS2984 | | 8-Azaguanine | Nucleotides and derivatives |
| mws1060 | | 9-(Arabinosyl)hypoxanthine | Nucleotides and derivatives |
| Wcjp001256 | | 9-Alpha-Ribofuranosyladenine* | Nucleotides and derivatives |
| Zmsp001773 | | 9-Arabinosyladenine* | Nucleotides and derivatives |
| Zmjn001030 | | Adenosine 2'-Phosphate | Nucleotides and derivatives |
| pmb0981 | | Adenosine 5'-monophosphate | Nucleotides and derivatives |
| pme0230 | | Adenosine* | Nucleotides and derivatives |
| mws1715 | | Cordycepin (3'-Deoxyadenosine)* | Nucleotides and derivatives |
| Zmyp000878 | | Crotonoside; 2-Hydroxyadenosine | Nucleotides and derivatives |
| mws0884 | | Cyclic 3',5'-Adenylic acid | Nucleotides and derivatives |
| pme3732 | | Cytidine | Nucleotides and derivatives |
| mws0255 | | Cytosine | Nucleotides and derivatives |
| pme1109 | | Guanine | Nucleotides and derivatives |
| pme1178 | | Guanosine | Nucleotides and derivatives |
| mws0609 | | Guanosine 3',5'-cyclic monophosphate | Nucleotides and derivatives |
| pmb0998 | | Guanosine 5'-monophosphate | Nucleotides and derivatives |
| pme0033 | | Hypoxanthine | Nucleotides and derivatives |
| pmb0532 | | Inosine 5'-monophosphate | Nucleotides and derivatives |
| pme0183 | | Isoguanine | Nucleotides and derivatives |
| pmb0964 | | Isopentenyladenine-7-N-glucoside | Nucleotides and derivatives |
| mws0984 | | L-Sepiapterin | Nucleotides and derivatives |
| Wdtp002974 | | N-(p-hydroxybenzyl) adenosine | Nucleotides and derivatives |
| Lmcp002302 | | N6-(2-Hydroxyethyl)adenosine* | Nucleotides and derivatives |
| Wcfp002357 | | N6-Threonylcarbamoyladenosine | Nucleotides and derivatives |
| MWS4354 | | N6-methyladenosine | Nucleotides and derivatives |
| pmb0197 | | N7-Methylguanosine | Nucleotides and derivatives |
| pmb0530 | | Nicotinic acid adenine dinucleotide | Nucleotides and derivatives |
| pme0264 | | Thymidine | Nucleotides and derivatives |
| mws0251 | | Thymine | Nucleotides and derivatives |
| mws0248 | | Uridine | Nucleotides and derivatives |
| pme3007 | | Uridine 5'-diphosphate | Nucleotides and derivatives |
| pmb2922 | | Uridine 5'-diphospho-D-glucose | Nucleotides and derivatives |
| Lmqn000780 | | Uridine 5'-diphospho-N-acetylglucosamine | Nucleotides and derivatives |
| pme3188 | | Uridine 5'-monophosphate | Nucleotides and derivatives |
| Zmjp000966 | | Vidarabine | Nucleotides and derivatives |
| pme0256 | | Xanthine | Nucleotides and derivatives |
| mws0668 | | Xanthosine | Nucleotides and derivatives |
| mws0976 | | β-Pseudouridine | Nucleotides and derivatives |
| Cmbn004440 | | 10-O-[(E)-Caffeoyl]-geniposidic acid | Terpenoids |
| MWSHC20196 | | 10α-Hydroxycadin-4-en-15-al | Terpenoids |
| Zmpn008194 | | 2,3-Dihydroxyurs-12-en-28-oic acid (Corosolic acid)* | Terpenoids |
| Saln003605 | | 3'-O-Feruloyl Swertiamarin | Terpenoids |
| Zjxp110301 | | 3-Hydroxy-3,7,11-trimethyldodeca-1,6E,10-trien-9-yl isobutyrate | Terpenoids |
| Qmyp101137 | | 4-[3-(β-D-Glucopyranosyloxy)butylidene]-3,5,5-trimethyl-2-cyclohexen-1-one | Terpenoids |
| pmp000703 | | 6'-O-D-Glucosylsweroside | Terpenoids |
| Hmcn000773 | | 6'-O-Glucosylaucubin | Terpenoids |
| Lmqp003432 | | 6,9-Dihydroxy-7-megastigmen-3-one | Terpenoids |
| Lafp004732 | | 7-Hydroxy-costol-glucoside | Terpenoids |
| Lafp005024 | | 7-Hydroxy-costol-malonyl glucoside | Terpenoids |
| Lhlp120627 | | Carvylacetate | Terpenoids |
| pmn001381 | | Eucommioside | Terpenoids |
| Lmdp002381 | | Genipin | Terpenoids |
| mws1565 | | Geniposide | Terpenoids |
| Cmlp002967 | | Gentiolactone | Terpenoids |
| pmp000970 | | Hispanolone | Terpenoids |
| Zmdn005723 | | Isoligustroside | Terpenoids |
| Cmmn013275 | | Isopimaric acid* | Terpenoids |
| pmp001048 | | Ixoroside | Terpenoids |
| MWSslk208 | | Kaurenoic Acid* | Terpenoids |
| Cmmn013378 | | Levopimaric acid* | Terpenoids |
| Lmjp004816 | | Loliolide | Terpenoids |
| Hmcn001415 | | Majoroside | Terpenoids |
| Lmbn014696 | | Pimaric acid* | Terpenoids |
| Xmdn002358 | | Rehmapicroside | Terpenoids |
| mws1526 | | Sweroside | Terpenoids |
| Hmlp013407 | | β-Eudesmol | Terpenoids |
| Smln004290 | | Aloesaponarin I | Quinones |
| Wmhn007355 | | Citreorosein | Quinones |
| Yhhn000821 | | kwanzoquinone C | Quinones |
| Wayn004109 | | 3'-O-Methylellagic acid 4-O-beta-D-glucoside | Tannins |
| Zbln006102 | | 3,3'-O-Dimethylellagic Acid | Tannins |

Table S3 The 38 identified differentially abundant steroid compounds.

| Index | Compounds | Class |
| --- | --- | --- |
| Lazp004368 | Triglucosyl 3-epiruscogenin-3-O-β-D-glucopyranoside | Steroidal saponins |
| Zjzp081873 | Sarsasapogenin-Glc-Glc-Glc-Glc-Xyl | Steroidal saponins |
| Jmyn004445 | Furost-5-ene-3,22,26-triol-3-O-glucosyl(1→2)[xylosyl(1→3)]glucosyl(1→4)galactoside-26-O-glucoside (Timosaponin H1) | Steroidal saponins |
| Zjxp101210 | Spirost-3,12-diol-3-O-glucosyl(1→2)galactoside | Steroidal saponins |
| Jmyn006303 | Spirost-5-ene-3,14-diol-3-O-glucosyl(1→2)[xylosyl(1→3)]glucosyl(1→4)galactoside | Steroidal saponins |
| Jmyp003897 | Spirost-5-ene-3,14-diol-3-O-glucosyl(1→2)[glucosyl(1→3)]glucosyl(1→4)galactoside | Steroidal saponins |
| Zjhp082648 | Spirost-5-ene-3,14-diol-3-O-glucosyl(1→2)glucosyl(1→4)galactoside (Polygoside A; Polygoside B) | Steroidal saponins |
| Zjhp082688 | Spirost-5-en-3-ol-3-O-glucosyl(1→2)[glucosyl(1→3)]glucosyl(1→4)galactoside* | Steroidal saponins |
| Layp006398 | pennogenin 3-O-α-L-rhamnopyranosyl-(1→4)-α-L-rhamnopyranosyl-(1→4)[O-α-L-rhamnopyranosyl-(1→2)]-β-D-glucopyranoside | Steroidal saponins |
| Zmcp006626 | Pennogenin-3-O-glucoside | Steroidal saponins |
| Zjhp082618 | Isochiapagenin-Glc | Steroidal saponins |
| Zjhp083005 | iso-Pennogenin-Glc-Glc-Glc-acetyl Glc | Steroidal saponins |
| Zjhp083010 | 14-Dehydroxy-neoprazerigenin A-Glc-Glc-Glc-Ara-Ara | Steroidal saponins |
| Zjbp081705 | 1,2-dihydroxy-Diosgenin-Glc | Steroidal saponins |
| Zjhp082612 | 14-Dehydroxy-neoprazerigenin A-Glc | Steroidal saponins |
| Zjhp082665 | 14-Dehydroxy-neoprazerigenin A-Glc-Glc-Glc-Xyl | Steroidal saponins |
| Zjhp082668 | iso-14-Dehydroxy-neoprazerigenin A-Glc-Xyl-Glc-Glc | Steroidal saponins |
| Zjzp081819 | 2-Hydroxy diosgenin-Glc-Glc | Steroidal saponins |
| Zjzp081866 | 2-Hydroxy diosgenin-Glc-Glc-Glc-Glc | Steroidal saponins |
| Layp003502 | Glucosyl-hecogenin 3-O-{O-β-D-glucopyranosyl-(1→2)-O-[β-D-xylopyranosyl-(1→3)]-O-β-D-glucopyranosyl-(1→4)-β-D-galactopyranoside}* | Steroidal saponins |
| Lhlp102623 | Hydroxydiosgenin glucose xylose glucoside* | Steroidal saponins |
| Zjhp082628 | Diosgenin-Gal-Glc | Steroidal saponins |
| Zjzp081862 | Diosgenin-Glc-Glc-Glc-Glc | Steroidal saponins |
| Zjzp081871 | Diosgenin-Glc-Glc-Glc-Glc-Xyl* | Steroidal saponins |
| Hmdp003288 | Trillin-6'-O-sophorotrioside* | Steroidal saponins |
| Hmdp003401 | Trillin-6'-O-glucoside | Steroidal saponins |
| Zjhp082615 | iso-Diosgenin-Gal | Steroidal saponins |
| Zjhp082614 | iso-Diosgenin-Glc | Steroidal saponins |
| Ladp003715 | 26-O-β-D-glucopyranose Glucosyl-(25R)-△5(6)-ene-16,22-diketone-furostane-3β,26-diol-3-O-α-L-rhamnosyl-(1→4)-O-α-L-rhamnosyl-(1→4)-[O-α-L-rhamnosyl-(1→2)]-O-β-D-pyranGlucoside* | Steroidal saponins |
| Zjcp102112 | 26-O-Glucosyl-3,20,26-trihydroxyfurostan-5,22-diene-3-O-rhamnosyl(1→4)rhamnosyl(1→4)rhamnosyl(1→4)glucoside (Padelaoside C)* | Steroidal saponins |
| Zjcp102111 | 3,20,26-Trihydroxy-furostan-5,22-diene-3-O-rhamnosyl-(1→2)-[rhamnosyl(1→4)rhamnosyl(1→4)]glucoside-26-O-glucoside (Smilaxchinoside B) | Steroidal saponins |
| Zjhp083018 | 3-Hydroxy-cholest-5,14,16-trien-22-one-3-O-glucosyl(1→2)[xylosyl(1→3)]glucosyl(1→4)galactoside-26-O-glucoside (Polygonatumoside A) | Steroidal saponins |
| Zjcp102008 | Parisvietnaside C/chonglouoside SL-19 | Steroidal saponins |
| Qadp004840 | Typaspidoside L* | Steroidal saponins |
| Jmyp005224 | Furost-5,20(22)-diene-3,26-diol-26-O-glucoside | Steroidal saponins |
| Qadp004838 | Aspidistrin | Steroidal saponins |
| Lsjp211306 | Spirost-4-en-3-one (Diosgenone) | Steroid |
| Zjzp081802 | Spirostan-3-ol (Isosarsapogenin) | Steroid |

Table S4 The 56 identified differentially abundant flavonoid compounds.

| Index | Compounds | Class |
| --- | --- | --- |
| Hmhp006479 | 2-(2,4-dihydroxybenzyl)-6,8-dihydroxy-7-methyl-3,4-dihydronaphthalen-1(2H)-one | Other Flavonoids |
| Lmtp003948 | 3',5',5,7-Tetrahydroxy-4'-methoxyflavanone-3'-O-glucoside | Flavones |
| Lmsp004301 | 3',5,5',7-Tetrahydroxyflavanone-7-O-glucoside* | Flavanones |
| Jmyn006479 | 3,5,7-Trihydroxy-6,8-dimethyl-3-(4'-hydroxybenzyl)-chroman-4-one (Polygonatone C) | Other Flavonoids |
| mws0914 | 3,5,7-Trihydroxyflavanone (Pinobanksin)* | Flavanonols |
| Lamp007163 | 4'-Demethyleucomin glucoside* | Other Flavonoids |
| pmn001477 | 4-C-Glucose-1,3,6-trihydroxy-7-methoxyxanthone | Other Flavonoids |
| pmp000172 | 5,2'-Dihydroxy-7,8-dimethoxyflavone glycoside | Flavones |
| Lajp005753 | 5,3'-dihydroxy-6,7,4'-trimethoxyflavone-8-O-β-D-glucoside | Flavones |
| Zjhp090618 | 5,7-Dihydroxy-6,8-dimethyl-3-(2',4'-hydroxybenzyl)chroman-4-One* | Other Flavonoids |
| Jmyp005575 | 5,7-Dihydroxy-6,8-dimethyl-3-(3'-hydroxy-4'-methoxybenzyl)-chroma4-one* | Other Flavonoids |
| Jmyn006493 | 5,7-Dihydroxy-6,8-dimethyl-3-(4'-hydroxybenzyl)-chromone-4-one | Other Flavonoids |
| Zjhn090628 | 5,7-Dihydroxy-8-methyl-3-(2',4'-dihydroxybenzyl)-chroman-4-one | Other Flavonoids |
| Zjhp090620 | 5,7-dihydroxy-6,8-dimethyl-3(R,S)-(3'-hydroxy-4'-methoxybenzyl)-chroman-4-one* | Other Flavonoids |
| Zjmp102041 | 5,7-dihydroxy-6,8-dimethyl-3-(4'-hydroxy-3'-methoxybenzyl)chroman-4-one* | Other Flavonoids |
| Zjhp090633 | 5,7-dihydroxy-6-methoxyl-8-methyl-3-(2',4'-dihydroxybenzyl)-chroman-4-one | Other Flavonoids |
| Lajp005898 | 5,8,3'-trihydroxy-6,7,4'-trimethoxyflavone-8-O-β-D-glucoside | Flavones |
| Zjhp090617 | 5-hydroxy-3-(2-hydroxy-4-methoxybenzyl)-7-methoxychroman-4-one | Other Flavonoids |
| Zjhp090623 | 5-hydroxy-7-methoxy-6,8-dimethyl-3-(2'-hydroxy-4'-methoxybenzyl)-chroman-4-one | Other Flavonoids |
| Hmhp006830 | 6,8-Dihydroxy-2-(2-hydroxy-4-methoxybenzyl)-7-methyl-3,4-dihydronaphthalen-1(2H)-one* | Other Flavonoids |
| Hmhp006887 | 6,8-Dihydroxy-2-(4-hydroxybenzyl)-5,7-dimethyl-3,4-dihydronaphthalen-1(2H)-one | Other Flavonoids |
| Hagn003556 | 7-Benzyloxy-5-hydroxy-3',4'-methylenedioxyflavonoid | Flavones |
| Zjhp090625 | 7-O-β-D-glucopyranoside-5-hydroxy-3-(4'-hydroxybenzylidene)-chroman-4-one* | Other Flavonoids |
| Smdp007779 | 8-Demethylfarrerol | Flavanones |
| pmp000573 | Acacetin-7-O-galactoside | Flavones |
| pmp001288 | Acacetin-7-O-rutinoside (Linarin) | Flavones |
| pmb0626 | Apigenin-6-C-glucoside-7-O-Sophoroside | Flavones |
| ZBN0018 | Apigenin-7-O-(2''-O-glucuronyl)glucuronide-4'-O-glucuronide | Flavones |
| Zmhn001036 | Choerospondin | Flavanones |
| HJN055 | Dihydrocharcone-4'-O-glucoside | Chalcones |
| Lmlp005236 | Dihydrokaempferol-3-O-glucoside* | Flavanonols |
| Lmdn003665 | Farrerol-5,7-di-O-glucoside | Flavanones |
| Lmmp000897 | Gallocatechin-(4α→8)-gallocatechin | Flavanols |
| Lmmp003271 | Gossypetin-8-O-glucoside | Flavonols |
| pmb0645 | Hesperetin-6-C-glucoside-7-O-glucoside | Flavonols |
| Lmzp002365 | Hesperetin-7-O-glucoside* | Flavanones |
| pmb0618 | Hesperetin-8-C-glucoside-3'-O-glucoside | Flavonols |
| Lmnp002584 | Hispidulin-8-C-(2''-O-glucosyl)glucoside | Flavones |
| mws1033 | Homoeriodictyol | Flavanones |
| Wahp004841 | Homoeriodictyol-7-O-β-O-glucoside* | Flavanones |
| Zmcn003728 | Kaempferol-3,7-O-diglucoside | Flavonols |
| mws0919 | Kaempferol-3-O-rhamnoside (Afzelin)(Kaempferin) | Flavonols |
| Wagp004984 | Limocitrol 3-Glucoside | Flavones |
| Hmpp002612 | Luteolin-7-O-gentiobioside | Flavones |
| pme0376 | Naringenin (5,7,4'-Trihydroxyflavanone)* | Flavanones |
| pme2960 | Naringenin chalcone; 2',4,4',6'-Tetrahydroxychalcone | Chalcones |
| Cmxp003975 | Okanin-4'-O-glucoside(Marein)* | Chalcones |
| Zjmp102007 | Ophiopogonanone E | Other Flavonoids |
| pme1201 | Phloretin | Chalcones |
| mws2118 | Phloretin-2'-O-glucoside (Phlorizin) | Chalcones |
| Zjhp090608 | Polygonatone D | Other Flavonoids |
| Lcyp000677 | Prunetin-4'-O-glucoside(Prunitrin)* | Isoflavones |
| pmp000596 | Quercetin-3-O-(2''-O-galactosyl)glucoside | Flavonols |
| MWSHY0162 | Quercetin-3-O-sophoroside (Baimaside) | Flavonols |
| mws1172 | Trifolirhizin (Maackiain-3-O-glucoside) | Isoflavones |
| Zjmp102042 | iso-5,7-dihydroxy-6,8-dimethyl-3-(4'-hydroxy-3'-methoxybenzyl)chroman-4-one* | Other Flavonoids |

Table S5 Relative expression of proteins involved in the metabolism of steroid saponins.

| ID | Name | Relative expression | | | |
| --- | --- | --- | --- | --- | --- |
|  |  | WI | SP | SU | AU |
| Cluster-23337.2.p1 | FDFT1 | 0.18610546 | 0.333877998 | 0.302267124 | 0.258564323 |
| Cluster-19988.0.p1 | TM7SF2 | 0 | 0.245868386 | 0 | 0.04374219 |
| Cluster-13541.0.p1 | DHCR24-1 | 1.24762196 | 1.470485762 | 2.495410222 | 1.61698996 |
| Cluster-17039.0.p1 | DHCR24-2 | 0.334874895 | 0.578602568 | 0.507830376 | 0.663330164 |
| Cluster-4768.0.p1 | EBP-1 | 0.291286963 | 0.255802845 | 0.400850387 | 0.42649565 |
| Cluster-6060.0.p1 | EBP-2 | 0.828878419 | 1.155938519 | 1.447311157 | 1.100950891 |
| Cluster-22268.0.p1 | DHCR7 | 0.710162767 | 1.148630193 | 0.305826832 | 1.034194573 |
| Cluster-10954.0.p1 | CAS1 | 0.379432514 | 0.840185615 | 0.635926692 | 0.637238487 |
| Cluster-11356.0.p1 | 3BETAHSDD-1 | 0.655323968 | 0.546141858 | 0.685303995 | 0.821958894 |
| Cluster-17670.0.p1 | 3BETAHSDD-2 | 1.039369363 | 1.254807553 | 0.749493497 | 0.974040136 |
| Cluster-22930.0.p1 | SMT2-1 | 0.597289277 | 0 | 0.421350548 | 0.197775857 |
| Cluster-22930.1.p1 | SMT2-2 | 1.636154025 | 1.473276402 | 1.923841173 | 2.40288338 |

Table S6 Relative expression of proteins involved in the metabolism of flavonoids.

| ID | Name | Relative expression | | | |
| --- | --- | --- | --- | --- | --- |
|  |  | WI | SP | SU | AU |
| Cluster-17471.0.p1 | CHS-1 | 0 | 0.266448649 | 0.054079112 | 0 |
| Cluster-23495.0.p1 | CHS-2 | 0 | 0.183117218 | 0.027999124 | 0 |
| Cluster-24206.0.p1 | CHS-3 | 0.533993567 | 1.86248193 | 2.772077057 | 1.180243107 |
| Cluster-24206.1.p1 | CHS-4 | 0.449863444 | 2.421686602 | 2.109212189 | 0.691440056 |
| Cluster-14846.0.p1 | CCOMT | 0.666622041 | 0.831932062 | 1.669335377 | 0.800705018 |
| Cluster-3825.0.p1 | HCT-1 | 1.211348303 | 1.281321794 | 0.614635623 | 0.708542046 |
| Cluster-4390.0.p1 | HCT-2 | 0 | 0.474487655 | 0.414730235 | 0.235845117 |
| Cluster-8451.0.p1 | HCT-3 | 1.739735073 | 2.957027372 | 2.485545844 | 2.503867145 |
| Cluster-9805.0.p1 | PGT1-1 | 0.788427504 | 0.609490885 | 0.362806913 | 0.344225573 |
| Cluster-17235.0.p1 | PGT1-2 | 3.745764735 | 3.545735637 | 7.675759784 | 5.562320805 |
| Cluster-17235.1.p1 | PGT1-3 | 1.557982773 | 1.033246867 | 0.882094521 | 1.019084794 |
| Cluster-20623.0.p1 | PGT1-4 | 0.875098603 | 0.669595454 | 0.565217078 | 0.603129395 |
| Cluster-22954.0.p1 | CHI-1 | 2.962146435 | 3.338612486 | 5.946823296 | 5.351548516 |
| Cluster-24200.0.p1 | CHI-2 | 2.696083917 | 5.682403889 | 5.925621646 | 4.456798478 |
| Cluster-7598.0.p1 | CHI-3 | 0 | 0 | 0.31431813 | 0.124552078 |
| Cluster-1329.0.p2 | CHI-4 | 0 | 0.070756498 | 0.086627665 | 0.041202842 |
| Cluster-7981.0.p1 | FLS-1 | 1.620515437 | 1.028888796 | 0.9458518 | 1.037969421 |
| Cluster-9733.0.p1 | FLS-2 | 0.575998389 | 0.683311769 | 0.300954606 | 0.489730533 |
| Cluster-22620.0.p1 | CYP75B1 | 3.353825654 | 2.42467382 | 1.893397544 | 2.142857907 |
| Cluster-848.0.p1 | HIDH-1 | 7.275340037 | 6.386048646 | 6.250310519 | 4.71678476 |
| Cluster-15683.0.p1 | HIDH-2 | 2.281987871 | 1.347021623 | 0.447094688 | 0.557914273 |
| Cluster-21691.1.p1 | HIDH-3 | 8.646247443 | 2.403699641 | 3.523048665 | 3.286440491 |
| Cluster-19934.3.p1 | IF7MAT-1 | 5.598240188 | 3.075481718 | 3.397425317 | 4.870234722 |
| Cluster-19934.1.p1 | IF7MAT-2 | 0.28384583 | 0 | 0.038172204 | 0.155729515 |
| Cluster-17297.0.p1 | I'2H-1 | 0.154612952 | 0 | 0.200915679 | 0.165082296 |
| Cluster-18386.1.p2 | I'2H-2 | 0.048924339 | 0.443091693 | 0.150355767 | 0.112796476 |
| Cluster-16877.0.p1 | PTS-1 | 3.047964209 | 2.673716725 | 4.787309237 | 6.207867643 |
| Cluster-18150.3.p1 | PTS-2 | 0.898768195 | 4.09919101 | 0.520577482 | 0.399615092 |
| Cluster-22653.0.p1 | UGT73C6 | 0.967606504 | 0.518661228 | 0.439219895 | 0.371157256 |
