# Supplementary material for: Integrated metabolomics and proteomics analysis reveals the accumulation mechanism of bioactive components in Polygonatum odoratum
Source: Front Plant Sci. 2024 Dec 20;15:1487613. doi: 10.3389/fpls.2024.1487613 (PMC11696735; doi:10.3389/fpls.2024.1487613)
Supplement: Supplementary file 2 [file DataSheet1.docx]

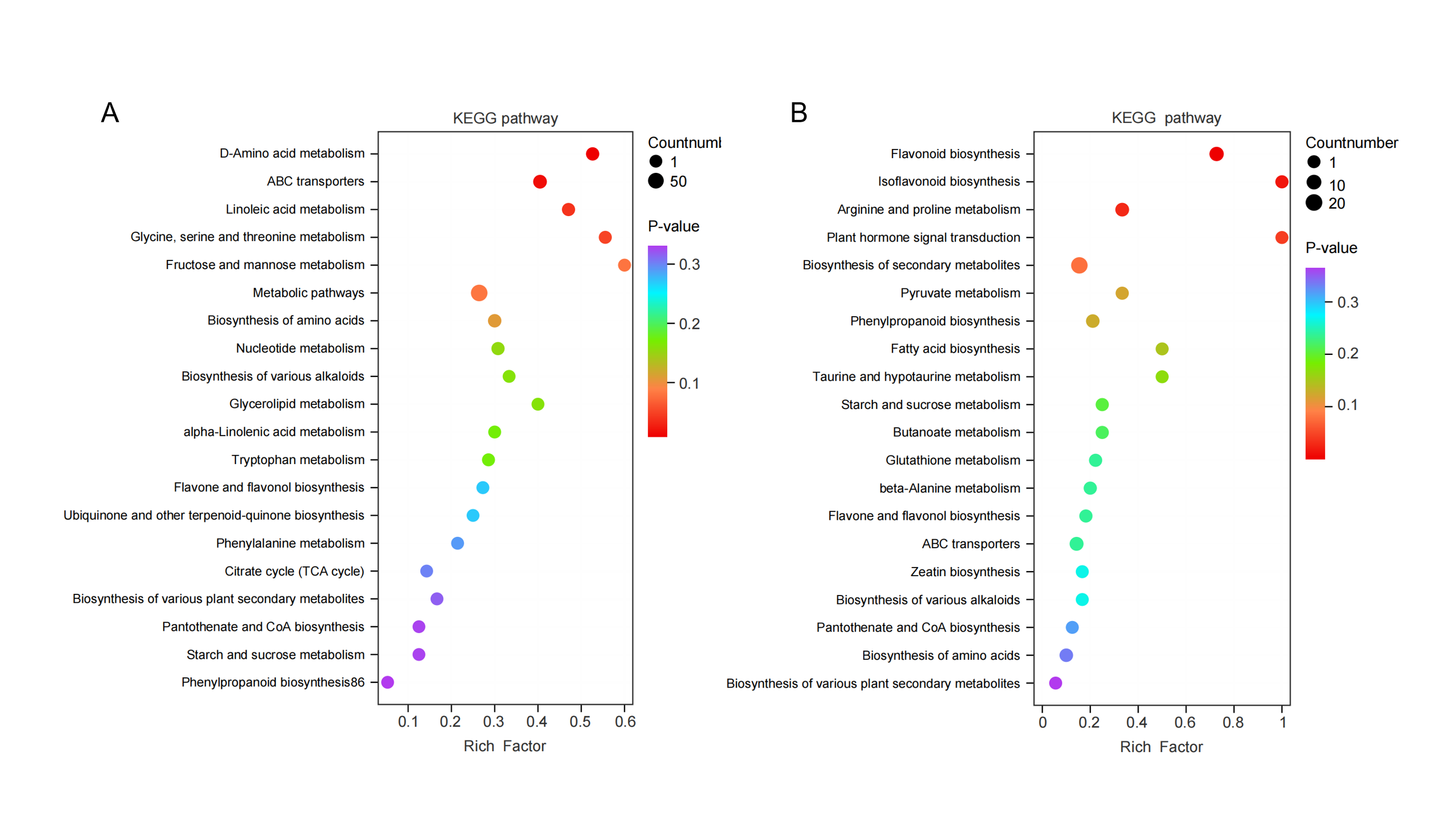


Fig. S1 Changes of metabolite expression. **(A)** KEGG pathway enrichment analysis of subcluster 1 metabolites. **(B)** KEGG pathway enrichment analysis of subcluster 3 metabolites.


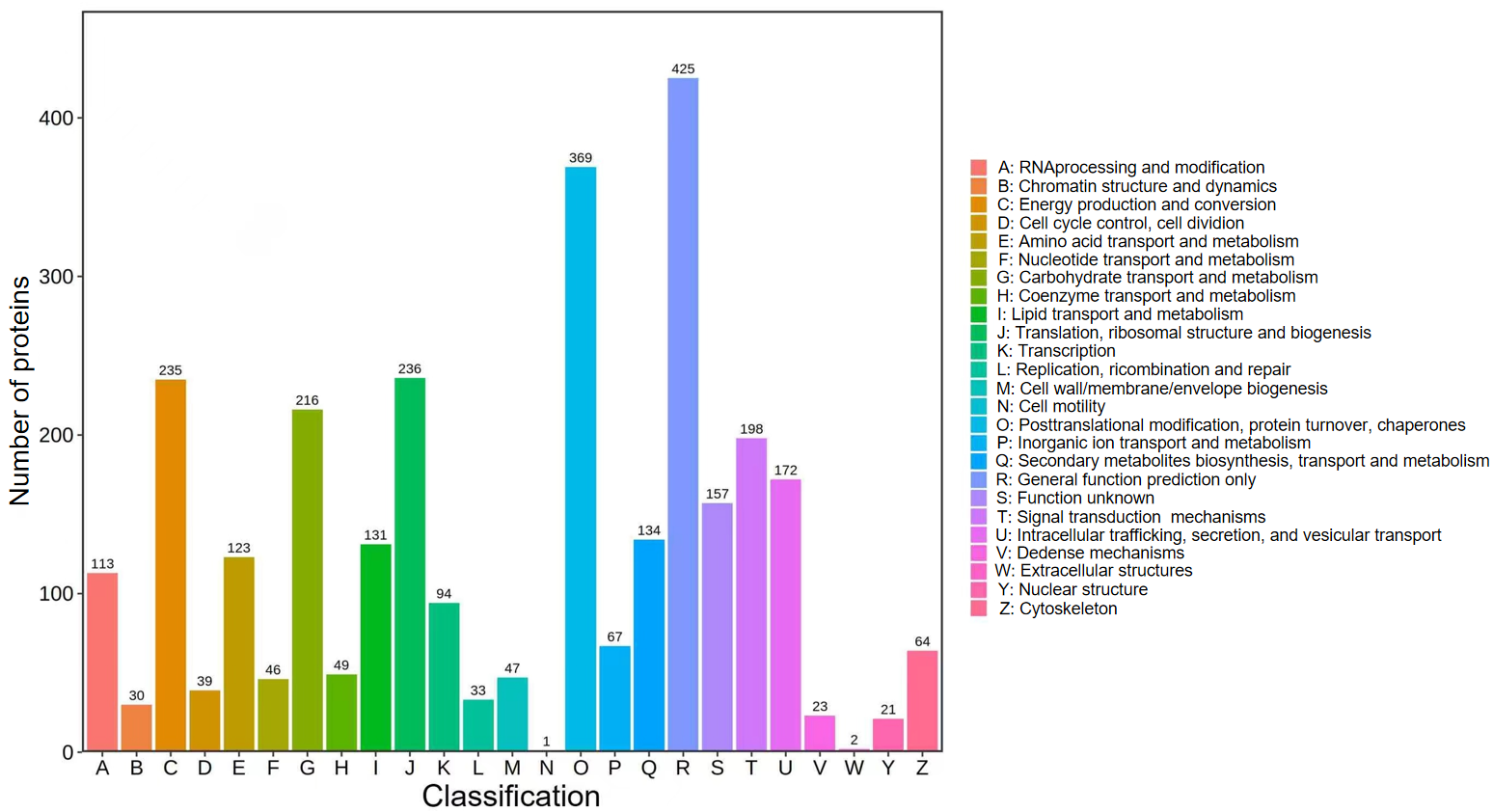


Fig. S2 COG functional annotation of differentially expressed proteins. The X-axis represents protein classification, and the y-axis represents quantity.


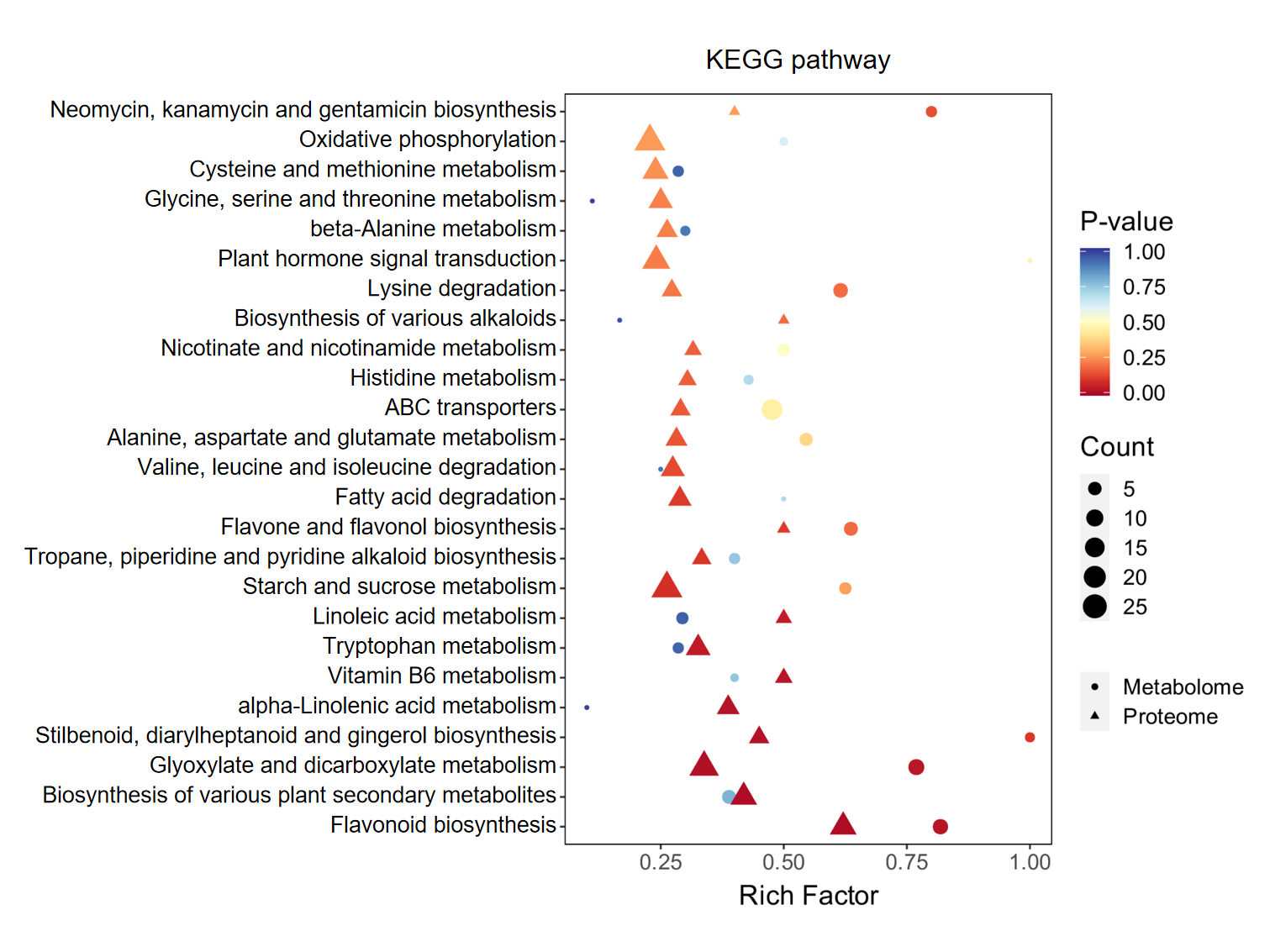


Fig. S3 The top 25 KEGG pathways enriched with metabolites and proteins (ranked by P-value).


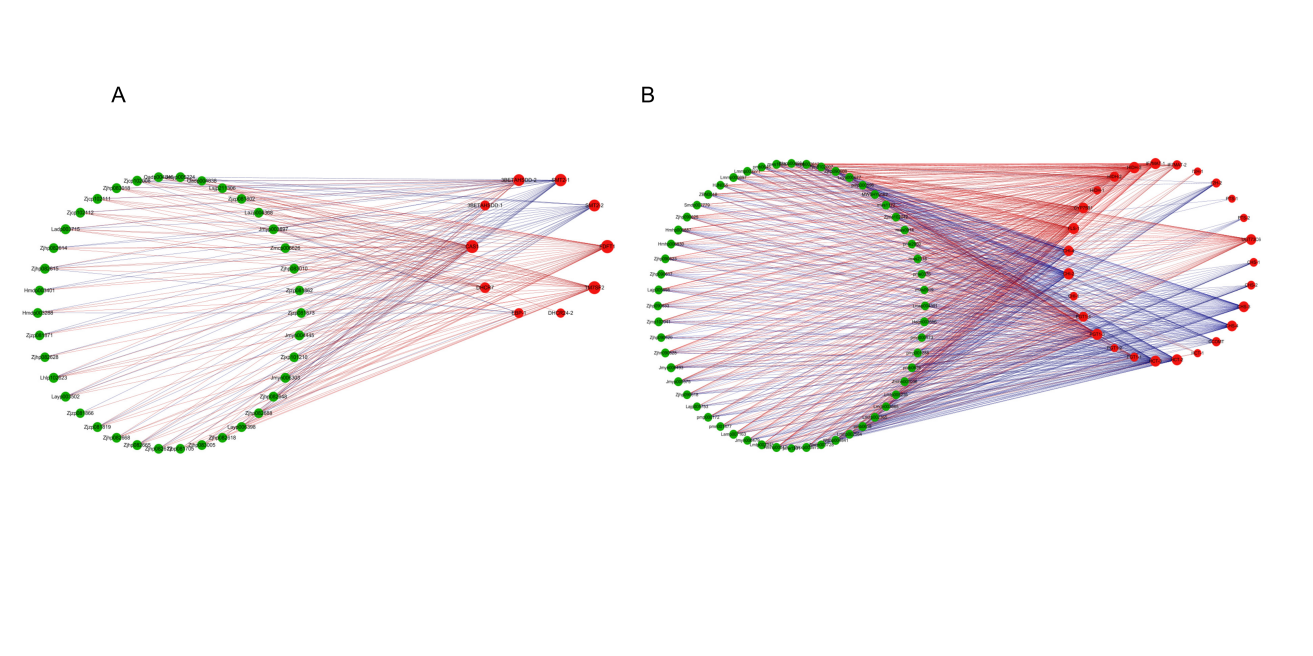


Fig. S4 **(A)** Pearson correlation analysis of steroid saponins and differentially expressed proteins. **(B)** Pearson correlation analysis of flavonoids and differentially expressed proteins. The red circle represents the protein name, and the green circle represents the steroid saponin ID. The red line represents positive correlation, the blue line represents negative correlation, and the size of the circle represents the strength of the correlation.


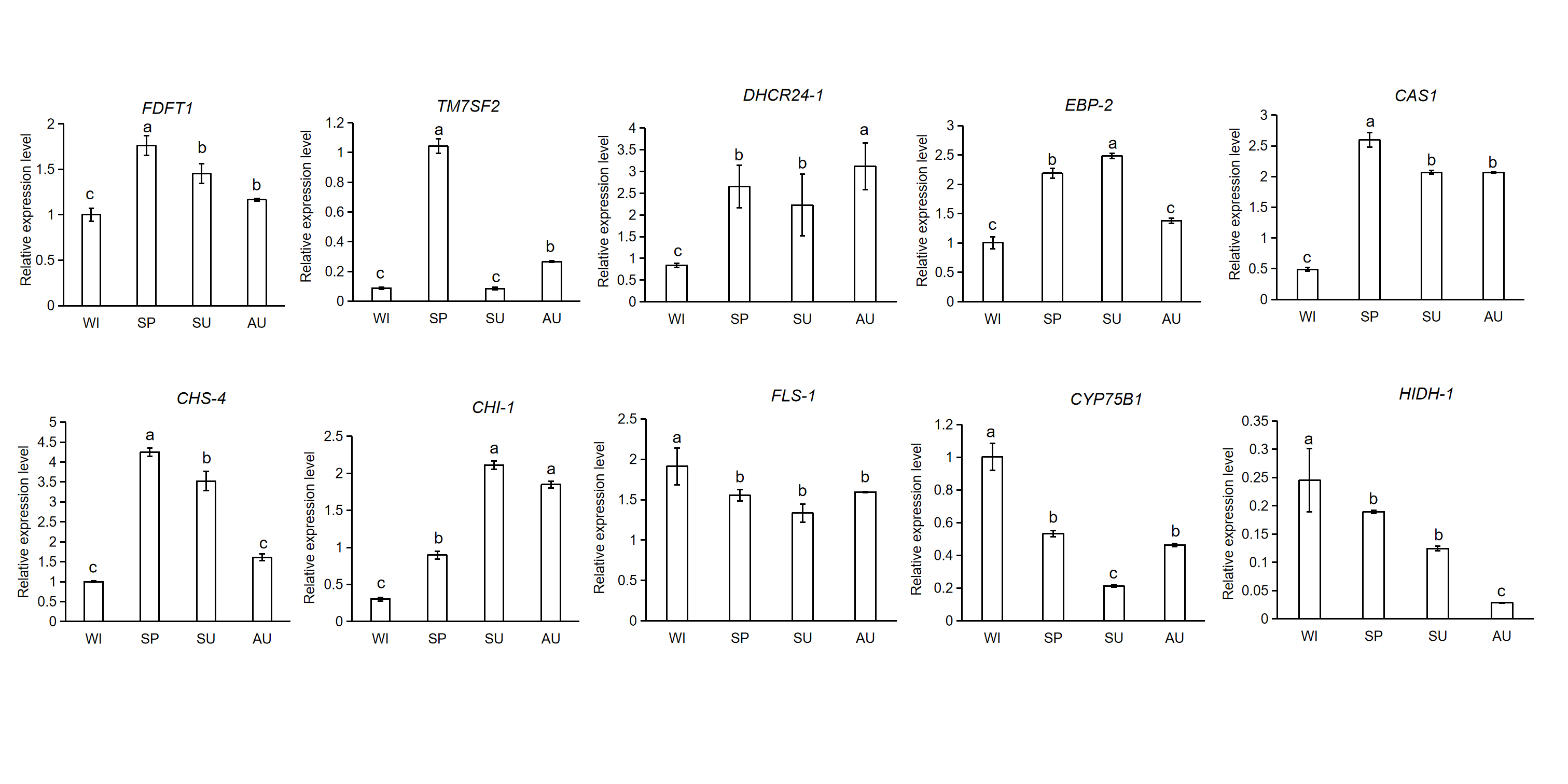


Figure S5. Real time fluorescence quantitative analysis of gene expression analysis.qRT-PCR analysis of several genes involved in the biosynthesis of steroidal saponins and flavonoids. *FDFT1*, farnesyl-diphosphate farnesyltransferase; *TM7SF2*, Δ14-sterol reductase; *DHCR24*, Δ24-sterol reductase; *EBP*, cholestenol Δ-isomerase; *CAS1*, cycloartenol synthase; *CHS*, chalcone synthase; *CHI*, chalcone isomerase; *FLS*, flavonol synthase; *CYP75B1*, flavonoid 3'-monooxygenase; *HIDH*, 2-hydroxyisoflavanone dehydratase.
